# Complex Evolution of Light-Dependent Protochlorophyllide Oxidoreductases in Aerobic Anoxygenic Phototrophs: Origin, Phylogeny, and Function

**DOI:** 10.1093/molbev/msaa234

**Published:** 2020-09-15

**Authors:** Olga Chernomor, Lena Peters, Judith Schneidewind, Anita Loeschcke, Esther Knieps-Grünhagen, Fabian Schmitz, Eric von Lieres, Roger Jan Kutta, Vera Svensson, Karl-Erich Jaeger, Thomas Drepper, Arndt von Haeseler, Ulrich Krauss

**Affiliations:** 1 Center for Integrative Bioinformatics Vienna, Max Perutz Labs, University of Vienna, Medical University of Vienna, Vienna, Austria; 2 Institut für Molekulare Enzymtechnologie, Heinrich-Heine-Universität Düsseldorf, Forschungszentrum Jülich GmbH, Jülich, Germany; 3 Institute of Bio- and Geosciences IBG-1: Biotechnology, Forschungszentrum Jülich GmbH, Jülich, Germany; 4 Institut für Physikalische und Theoretische Chemie, Universität Regensburg, Regensburg, Germany; 5 Faculty of Computer Science, University of Vienna, Vienna, Austria

**Keywords:** light-driven enzyme, chlorophyll biosynthesis, evolution, aerobic anoxygenic phototrophic bacteria, photosynthesis

## Abstract

Light-dependent protochlorophyllide oxidoreductase (LPOR) and dark-operative protochlorophyllide oxidoreductase are evolutionary and structurally distinct enzymes that are essential for the synthesis of (bacterio)chlorophyll, the primary pigment needed for both anoxygenic and oxygenic photosynthesis. In contrast to the long-held hypothesis that LPORs are only present in oxygenic phototrophs, we recently identified a functional LPOR in the aerobic anoxygenic phototrophic bacterium (AAPB) *Dinoroseobacter shibae* and attributed its presence to a single horizontal gene transfer event from cyanobacteria. Here, we provide evidence for the more widespread presence of genuine LPOR enzymes in AAPBs. An exhaustive bioinformatics search identified 36 putative LPORs outside of oxygenic phototrophic bacteria (cyanobacteria) with the majority being AAPBs. Using in vitro and in vivo assays, we show that the large majority of the tested AAPB enzymes are genuine LPORs. Solution structural analyses, performed for two of the AAPB LPORs, revealed a globally conserved structure when compared with a well-characterized cyanobacterial LPOR. Phylogenetic analyses suggest that LPORs were transferred not only from cyanobacteria but also subsequently between proteobacteria and from proteobacteria to *Gemmatimonadetes*. Our study thus provides another interesting example for the complex evolutionary processes that govern the evolution of bacteria, involving multiple horizontal gene transfer events that likely occurred at different time points and involved different donors.

## Introduction

It is widely accepted that photosynthesis, the process by which photosynthetic organisms convert light energy into chemical energy, has evolved early on in Earth’s history (Blankenship 2010; Hohmann-Marriott and Blankenship 2011). There are two types of photosynthesis: oxygenic and anoxygenic. In oxygenic photosynthesis, performed by cyanobacteria and plants, light energy is used for the oxidation of water, thereby releasing oxygen, electrons, and protons. In contrast, in anoxygenic photosynthesis, performed by anoxygenic phototrophic bacteria (APBs), for example, hydrogen sulfide, hydrogen or other organic substrates are used as electron donors, while the process does not generate oxygen (Hanada 2016). Anoxygenic photosynthesis hereby likely predates oxygenic photosynthesis (Hohmann-Marriott and Blankenship 2011), with the latter being assumed to have first evolved in an ancestor of cyanobacteria (Blankenship 2010; Hohmann-Marriott and Blankenship 2011; Cardona et al. 2015). Although the timing and mechanism by which oxygenic photosynthesis arose is still debated (Soo et al. 2017; Martin et al. 2018; Cardona 2019), its emergence undoubtedly led to the oxygenation of the primordial atmosphere, thereby laying the foundations for life on Earth as we know it today.

At the foundation of photosynthesis, light‐absorbing pigments such as chlorophylls (Chls) and carotenoids, as part of photosynthetic reaction centers (RCs), enable the harvesting of light energy (Blankenship 2010). Chls hereby represent the main class of light‐harvesting pigments essential for all phototrophic organisms of the bacterial and eukaryotic domains. It is therefore not surprising that the evolution of Chl biosynthesis, which is a branch in the synthesis pathway of modified tetrapyrroles (Bryant et al. 2020), is linked to the evolution of photosynthesis.

A key step in the complex biosynthesis pathway of Chls and bacteriochlorophylls (Bchls) is the reduction of the C17=C18 double bond of the protochlorophyllide (Pchlide) D-ring to yield chlorophyllide (Chlide) (Beale 1999; Blankenship 2010). During evolution of (bacterio)chlorophyll biosynthesis two structurally distinct enzyme systems have emerged capable of catalyzing this reaction, namely dark-operative protochlorophyllide oxidoreductases (DPORs) and light-dependent protochlorophyllide oxidoreductases (LPORs) (Suzuki and Bauer 1995; Schoefs and Franck 2003; Yang and Cheng 2004; Reinbothe et al. 2010). For a long time, it was widely accepted that APBs contain only DPORs (Suzuki and Bauer 1995; Schoefs and Franck 2003; Yang and Cheng 2004), whereas plants, with the exception of gymnosperms, contain only LPORs (Yang and Cheng 2004; Sousa et al. 2013). The majority of cyanobacteria, ferns, mosses, gymnosperms, and algae have both enzyme systems (Fujita 1996). DPORs are multisubunit protein complexes consisting of three subunits (called BchN, BchB, BchL in APBs and ChlN, ChlB, ChlL in oxygenic phototrophs), which contain iron–sulfur clusters, and are therefore sensitive to oxygen (Muraki et al. 2010). They convert Pchlide in an ATP‐dependent process independent of light (Bröcker et al. 2010). In contrast, LPORs are oxygen-insensitive, single‐component NADPH‐dependent enzymes of the short-chain dehydrogenase (SDR) family of enzymes (Townley et al. 2001), which convert Pchlide in a strictly light‐dependent process (Schoefs and Franck 2003). DPORs, as the evolutionary older enzymes, likely evolved in the anoxygenic environment of the early Earth and share a common ancestor with nitrogenase-like enzymes (Muraki et al. 2010; Hu and Ribbe 2015). In contrast, LPORs are considered as evolutionary younger enzymes, which were speculated to have evolved in cyanobacteria at about the time of the great oxygenation event (Yamazaki et al. 2006). This hypothesis is primarily based on their oxygen insensitivity and their presumed absence in APBs (Yamazaki et al. 2006).

The recent discovery of a functional LPOR in the aerobic anoxygenic phototrophic α-proteobacterium *Dinoroseobacter shibae* DFL12^T^ (*Ds*LPOR; Kaschner et al. 2014) challenged this hypothesis. Aerobic anoxygenic phototrophic bacteria (AAPBs) are a ubiquitous group of marine microbes, related to facultative anaerobic purple non-sulfur bacteria (Biebl et al. 2005). In contrast to classical APBs like *Rhodobacter* sp., AAPBs can perform anoxygenic photosynthesis in the presence of atmospheric oxygen (Yurkov and Beatty 1998). It seems therefore a reasonable adaption for AAPBs to possess an LPOR enzyme system to enhance Bchl synthesis under aerobic conditions. In another recent study (Kasalicky et al. 2017), additional six LPORs in nonoxygenic photosynthetic bacteria were reported. However, their functionality was not investigated.

Here, we show that LPORs are more common among AAPBs than originally assumed. We identify 36 LPORs outside of oxygenic phototrophic genera, verify activity for 10 out of 11 tested AAPB LPORs, provide biochemical and solution structural data of six and two AAPB LPORs, respectively, and discuss evolutionary processes that have led to their wide distribution outside of oxygenic phototrophic bacteria.

## Results and Discussion

### Identification of LPORs Outside of Oxygenic Phototrophs

**Fig. 1.**
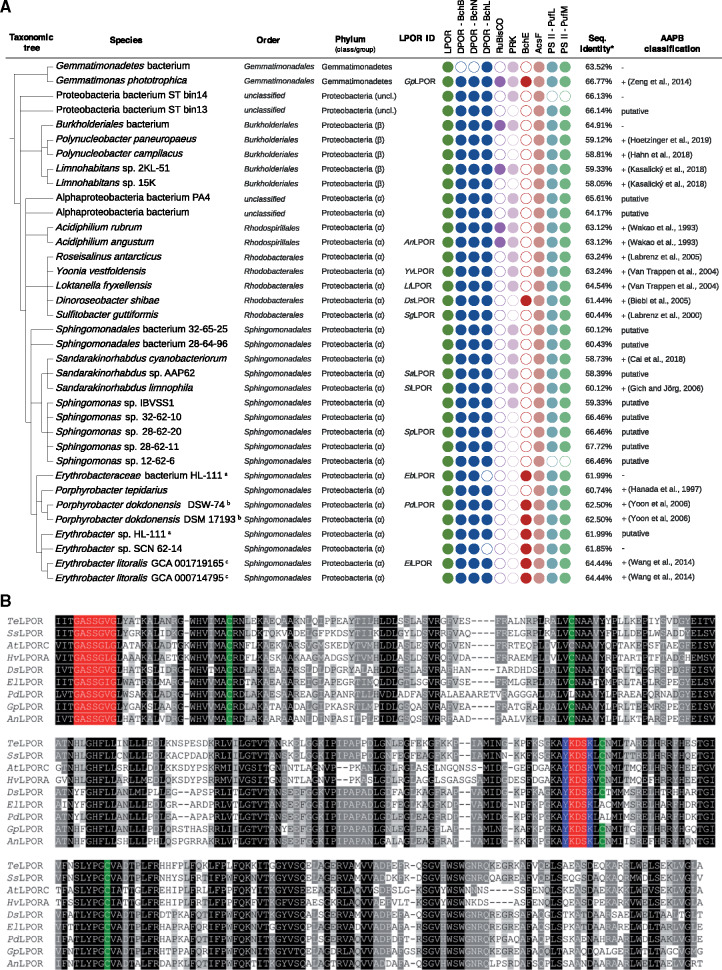
(*A*) Taxonomic distribution of the putative LPORs of nonoxygenic phototrophic origin, AAPB marker gene presence/absence analysis and AAPB classification. ^a,b,c^Two identical LPORs each. Bacteria, which were previously reported to possess LPOR, are *Gemmatimonas phototrophica, Limnohabitans* sp. 15K*, Yoonia vestfoldensis, Dinoroseobacter shibae, Sulfitobacter guttiformis, Porphyrobacter dokdonensis*, and *Erythrobacter litoralis.* The LPOR ID identifies the LPOR candidates for which functional tests have been performed. Presence (filled circles) and absence (empty circles) of marker genes verified for: LPOR, DPOR (BchB, BchN, BchL subunits), RuBisCO (large chain), PRK, oxygen-independent (BchE) and oxygen-dependent (AcsF) magnesium-protoporphyrin IX monomethyl ester oxidative cyclase; and subunit M (PufM) and L (PufL) of pheophytin-quinone-based type II RC. Neither RC type I PsaA/PsaB nor RC type II subunits PsbA/PsbD were identified in the genomes of the considered bacteria. Therefore, they are not shown in (*A*). The AAPB classification is according to the previous studies (references are provided) or according to the genetic marker analysis presented in the current study (denoted by “putative,” as it has to be confirmed by lab experiments). The minus sign denotes species, which could not be assigned to AAPBs based on the presence–absence pattern of gene markers. *The sequence similarity refers to BlastP percent identity to LPOR sequence of *Synechocystis* sp. PCC 6803. (*B*) Multiple sequence alignment of selected LPORs of oxygenic (*Te*LPOR: LPOR of *Thermosynechococcus elongatus* BP-1; *Ss*LPOR: LPOR of *Synechocystis* sp. PCC6803; *At*LPORC: LPOR C of *Arabidopsis thaliana*, HvLPORA: LPOR A of *Hordeum vulgare*) and nonoxygenic (sequences identified by LPOR ID, see panel *A*) phototrophic origin. Highlighted positions are: the conserved NADPH‐binding motif (shown in red) (Buhr et al. 2008), four conserved cysteine residues supposed to be involved in substrate binding (in green) (Menon et al. 2010), and the catalytic Tyr and Lys residues (in blue) (Menon et al. 2009).

Prompted by our recent identification of a genuine LPOR in the AAPB *D. shibae* (*Ds*LPOR) (Kaschner et al. 2014), we comprehensively analyzed 116,919 bacterial genomes covering all bacterial phyla (available in GenBank; Clark et al. 2016); as of July 17, 2018) for the presence of LPORs. Using the HMMER software and an LPOR hidden Markov model (HMM; see Materials and Methods and supplementary table S2, Supplementary Material online) we identified 36 LPORs outside of oxygenic phototrophs, confirming nine LPORs previously found in *D. shibae*, *Gemmatimonas phototrophica*, *Limnohabitans* sp. 15K, *Yoonia vestfoldensis*, *Sulfitobacter guttiformis*, *Porphyrobacter dokdonensis* (strains DSM 17193 and DSW-74), and *Erythrobacter litoralis* DSM 8509 (two LPORs in genomes: GCA_001719165.1 and GCA_000714795.1). The 27 newly found putative LPORs occur in: 20 α-proteobacteria, four β-proteobacteria, two unclassified proteobacteria, and one in *Gemmatimonadetes* (fig. 1*A*; supplementary table S1, Supplementary Material online).

To determine whether bacteria carrying putative LPORs are AAPBs, we scanned the corresponding genomes for the presence/absence of various genes (using HMMER as described above for LPOR; supplementary table S2, Supplementary Material online) typically associated with aerobic anoxygenic photosynthesis in the genomes, being well aware that the results of the bioinformatics analysis depend on the quality of the genomes provided. We scanned for the presence of three DPOR subunits BchL, BchN, and BchB proteins as markers of anoxygenic photosynthesis, whereas the absence of the large chain subunit of the ribulose-1,5-bisphosphat-carboxylase/-oxygenase (RuBisCO) and phosphoribulokinase (PRK) was used as a sign for AAPBs (Brinkmann et al. 2018). We also checked the presence of the oxygen-independent (BchE) and oxygen-dependent (AcsF) magnesium-protoporphyrin IX monomethyl ester oxidative cyclase enzymes as markers for aerobic and semiaerobic chlorophototrophs (Boldareva-Nuianzina et al. 2013; Zeng et al. 2014). Finally, we analyzed the genomes for the presence of photosynthetic RCs: FeS-based type I (PsaA and PsaB) and pheophytin-quinone-based type II (including subfamilies for the subunits M [PufM], L [PufL], PsbA/D1, and PsbD/D2). RC type I is typical for green-sulfur bacteria (Chlorobi), phototrophic Firmicutes, and phototrophic Acidobacteria, whereas RC type II is typical for non-sulfur green (Chloroflexi) and purple bacteria (phototrophic proteobacteria) (Overmann and Garcia-Pichel 2013; Zeng et al. 2014). Presence/absence patterns are shown in figure 1*A* and supplementary table S1, Supplementary Material online.

We call a bacterium (putative) AAPB, if it possesses the three DPOR subunits, RC type II and AcsF (and/or BchE), and lacks RuBisCO (and/or PRK). For 19 out of 36 bacteria carrying a putative LPOR, it was experimentally shown that they are AAPBs (fig. 1*A*) (Wakao et al. 1993; Hanada et al. 1997; Labrenz et al. 2000; Van Trappen et al. 2004; Biebl et al. 2005; Labrenz et al. 2005; Gich and Overmann 2006; Yoon et al. 2006; Wang et al. 2014; Zeng et al. 2014, 2015; Kasalicky et al. 2017; Cai et al. 2018; Hahn et al. 2018; Hoetzinger et al. 2019). Sixteen of 19 experimentally verified AAPBs fulfill the above rule (fig. 1*A*), which means that the rule is conservative in the sense that we may miss some AAPBs. In summary, the majority of bacteria (31 out of 36) were assigned to AAPBs (see also supplementary section 1.1, Supplementary Material online). For simplicity, in the following we call all LPORs from figure 1*A* AAPB LPORs. With regard to sequence similarity (fig. 1*B*; full alignment: supplementary fig. S1, Supplementary Material online), all sequence features important for LPOR function (Townley et al. 2001; Buhr et al. 2008; Menon et al. 2009) are conserved between known and putative LPORs from plants, cyanobacteria, and AAPBs.

### AAPB LPORs Show Light-Dependent Activity

**Fig. 2.**
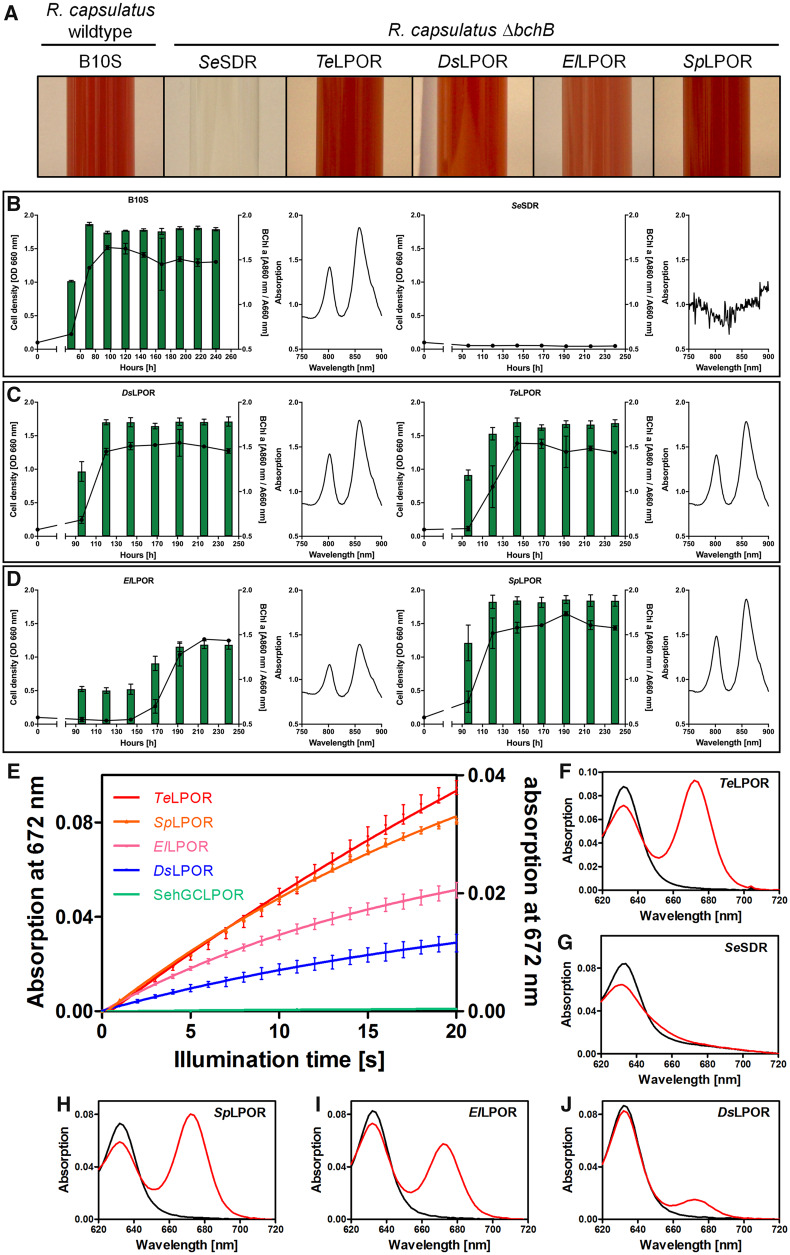
In vivo (*A*–*D*) and in vitro (*E*–*J*) functional tests to investigate light-dependent Pchlide conversion by (AAPB) LPORs. (*A*) Growth phenotype of *Rhodobacter capsulatus* wild-type strain and the Δ*bchB* (DPOR deficient) strain complemented with *Se*SDR as a negative control as well as known (*Te*LPOR, *Ds*LPOR) and exemplary putative LPORs (*El*LPOR, *Sp*LPOR). (*B*–*D*) Growth curves (black lines, OD_660nm_) and BChl *a* accumulation, measured as normalized in vivo absorption at 860 nm (green bars, OD_860nm_/OD_660nm_) over time of cultivation, as well as absorption spectra of cellular BChl *a* (normalized to the cell density, 240 h cultivation time). Assays were carried out for the same set of LPOR enzymes as shown in panel (*A*). (*E*) Exemplary absorption changes at 672 nm with illumination time, indicating Pchlide to Chlide turnover by known LPORs (*Te*LPOR, *Ds*LPOR) and selected putative AAPB LPORs (*Sp*LPOR, *El*LPOR). As the different enzymes showed variably high activities, the data are shown on two differently scaled *Y*-axes (*Te*LPOR, *Sp*LPOR, *El*LPOR, left ordinate; *Ds*LPOR, *Se*SDR, right ordinate). Error bars represent the standard deviation of the mean derived from three independent measurements. (*F*–*J*) Exemplary absorption spectra prior (black, pure Pchlide spectrum with Q_y_ band at λ  ≈  630 nm) and after 20 s of blue-light illumination (red, reduced Pchlide absorption and corresponding formed Chlide absorption with Q_y_ band at λ  ≈  672 nm) obtained using purified LPORs as indicated.

For functional characterization, we selected 11 putative AAPB LPORs from different taxonomic families (fig. 1 and supplementary table S1, Supplementary Material online; identified by LPOR ID). We hereby selected putative AAPB LPORs that did not derive from metagenomes and were included in the RefSeq database (hence having a low probability of representing an artifact). Additionally, we included: the AAPB LPOR from *D. shibae* (*Ds*LPOR) (Kaschner et al. 2014); two cyanobacterial LPORs—*Synechocystis* sp. PCC6803 (*Ss*LPOR) and *Thermosynechococcus elongatus* BP-1 (*Te*LPOR); as well as two plant LPORs—*Hordeum vulgare* (POR A; *Hv*LPORA) and *Arabidopsis thaliana* (POR C; *At*LPORC) (Heyes et al. 2000; Pattanayak and Tripathy 2002; McFarlane et al. 2005; Buhr et al. 2008) (supplementary table S1, Supplementary Material online). An actinobacterial SDR from *Saccharopolyspora erythraea* (*Se*SDR), which shares 33.4% identical positions with *Te*LPOR (7% gaps), was analyzed as negative control. LPOR function was investigated by in vitro assays using either crude cell extracts or purified protein and by an in vivo complementation assay using a *Rhodobacter capsulatus* strain which carried a deletion in the *bchB* gene encoding for one of the three DPOR subunits (Kaschner et al. 2014) (see also supplementary section 1.2, Supplementary Material online). We consider an LPOR as functional, if it was active in at least one of the assays. The results of exemplary in vitro and in vivo activity tests are shown in figure 2 (for all results, see supplementary figs. S2 and S3 [in vitro], figs. S4 and S5 [in vivo], and table S4, Supplementary Material online). The negative control, *Se*SDR was inactive in both assays (in vivo and in vitro). As reported previously (Kaschner et al. 2014), *Ds*LPOR was active in both assays. As positive controls, *Te*LPOR and *At*LPORC showed high and intermediate activity in both assays, whereas for *Ss*LPOR and *Hv*LPOR, low, unsteady or no activity was observed (for details about semiquantitative ranking, see supplementary table S4, Supplementary Material online). Intriguingly, 10 out of 11 tested putative AAPB LPORs showed activity in at least one of the two assays. Seven AAPB LPORs (*Eb*LPOR, *El*LPOR, *Sp*LPOR, *Pd*LPOR, *Lf*LPOR, *Yv*LPOR, and *An*LPOR) were active in both assays. *Sa*LPOR showed high activity in vivo but was inactive in vitro. *Sl*LPOR and *Gp*LPOR were active in vitro but inactive in vivo. *Sg*LPOR was the only putative AAPB LPOR without signs of activity. This observation can most likely be attributed to aggregation of the improperly folded enzyme in both *Escherichia coli* and *R. capsulatus*, as functionally important residues are conserved between *Sg*LPOR and the other AAPB LPORs (supplementary fig. S3, Supplementary Material online). In summary, LPOR functionality could be verified for 10 out of 11 tested AAPB LPORs, thus clearly demonstrating that functional LPORs are prevalent among AAPBs. Future work should include functional tests for β-proteobacterial LPORs of the order *Burkholderiales* (fig. 1*A*), which due to their later identification could not be tested as part of this study.

### Biochemical Properties of AAPB, Cyanobacteria, and Plant LPORs

**Fig. 3.**
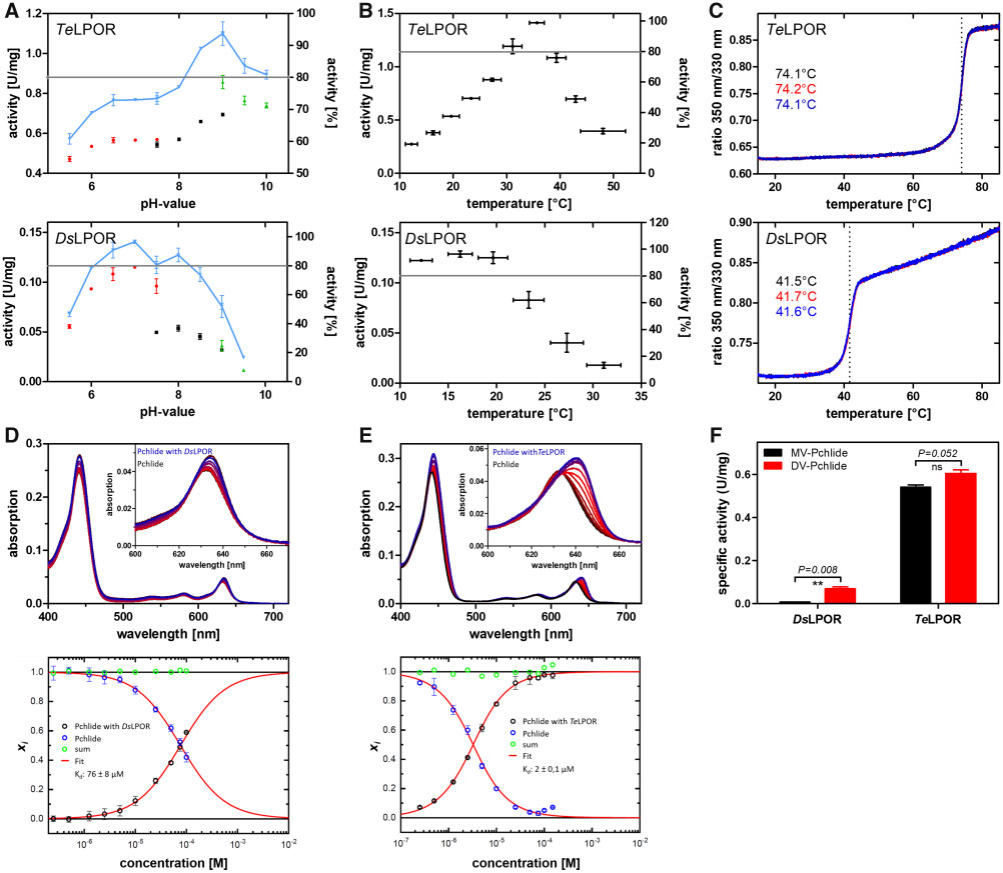
Exemplary biochemical characterization of *Ds*LPOR and *Te*LPOR, with regard to pH optima (*A*), temperature optima (*B*), temperature-dependent unfolding (*C*), *K*_d_ of the ternary LPOR/NADPH/Pchlide complex (*D*, *E*), and MV- and DV-Pchlide substrate acceptance (*F*). (*A*) Three suitable buffer systems covering different pH values were used: sodium phosphate buffer, red data points; Tris buffer, blue data points; glycine buffer, green data points. (*B*) Temperature optima were determined in Tris buffers whose pH was adjusted at the target temperature. The gray line marks the arbitrary 80% activity threshold that was set to derive the pH_optimum_ range and the *T*_optimum_ range for the respective enzyme (see table 1). (*C*) Temperature-dependent unfolding was monitored by DSF. Three independent measurements per enzyme are shown, and the obtained melting temperature (*T*_M_) is given. (*D*) The *K*_d_ of the ternary LPOR/NADPH/Pchlide holoprotein complex was determined by quantifying (lower panels in *D* and *E*) the red-shift of the Pchlide Q_y_ band due to complex formation (upper panels in *D* and *E*; free Pchlide in black; Pchlide+LPOR concentration series, shades of red; Pchlide+highest LPOR concentration, blue). (*F*) MV/DV-Pchlide preference determined as specific activity with 3.5 ± 0.15 µM MV- (black bars) and DV-Pchlide (red bars) as substrate. Error bars correspond to the standard deviation of the mean of three independent measurements. Statistical significance (two-tailed, paired *t*-test, *P* ≤ 0.05).

We next biochemically characterized six putative AAPB LPORs (*El*LPOR, *Eb*LPOR, *Pd*LPOR, *Gp*LPOR, *Sp*LPOR, and *Lf*LPOR), *Ds*LPOR, as well as the cyanobacterial *Te*LPOR and plant *At*LPORC. Unfortunately, we were unable to heterologously produce and purify other plant and cyanobacterial LPORs (*Hv*LPORA and *Ss*LPOR). All LPORs, which could be purified in sufficient quantity and purity, were characterized with regard to pH- and temperature-optima, temperature stability (temperature-dependent unfolding), dissociation constant *K*_d_ of Pchlide binding to the ternary LPOR/NADPH/Pchlide complex (see Materials and Methods for details), the influence of the reducing agent dithiotreitol (DTT) on the *K*_d_, and the preference for either monovinyl (MV)- and divinyl (DV)-Pchlide substrates. The latter aspect was tested because reactive thiol groups of cysteines have been implicated in either Pchlide binding or catalysis (Heyes et al. 2000). An exemplary overview of the respective data for the *Te*LPOR and *Ds*LPOR enzymes is shown in figure 3 (for all results see supplementary section 1.3; supplementary figs. S6–S10 and S13, Supplementary Material online). The complete set of characteristics is summarized in table 1.

**Table 1.** Comparison of Biochemical Properties of Selected AAPB LPORs, *Te*LPOR, and *At*LPORC.

	*El*LPOR	*Eb*LPOR	*Pd*LPOR	*Gp*LPOR	*Sp*LPOR	*Lf*LPOR	*Ds*LPOR	*Te*LPOR	*At*LPORC
Activity (U/mg)	0.41 ± 0.04	0.69 ± 0.01	0.48 ± 0.04	0.11 ± 0.001	0.58 ± 0.02	0.10 ± 0.01	0.09 ± 0.02	0.65 ± 0.02	0.02 ± 0.002
pH_optimum_ (pH)	8.5	9.0	8.0	8.0	8.0	9.0	7.0	8.5	8.5
pH_optimum_ range (pH)	2.5	2.5	2.5	2.5	2.5	3.0	2.0	1.5	1.5
*T*_optimum_ (°C)	23.3 ± 1.6	19.6 ± 1.5	19.6 ± 1.5	16.2 ± 1.3	23.3 ± 1.6	23.3 ± 1.6	16.2 ± 1.3	35.7 ± 1.4	27.3 ± 1.7
*T*_optimum_ range (°C)	7.1	3.7	7.1	7.4	3.7	7.7	7.4	4.6	3.8
*T*_M_ (°C)	46.3 ± 0.09	51.6 ± 0.04	54.4 ± 0.06	40.1 ± 0.09	34.7 ± 0.05	35.6 ± 0.07	41.6 ± 0.07	74.1 ± 0.06	35.1 ± 0.49
*K*_d_ (+DTT) (µM)	41 ± 1	44 ± 7	41 ± 2	134 ± 9	7 ± 2	5.7 ± 0.4	105 ± 7	2.0 ± 0.1	1.6 ± 0.2
*K*_d_ (−DTT) (µM)	56 ± 2	47 ± 3	38 ± 2	84 ± 20	7 ± 1	5.9 ± 0.9	76 ± 8	2.0 ± 0.1	1.6 ± 0.2
Activity MV (U/mg)	0.31 ± 0.01	0.22 ± 0.02	0.32 ± 0.01	0.12 ± 0.01	0.17 ± 0.01	0.38 ± 0.01	0.01 ± 0.001	0.54 ± 0.01	0.25 ± 0.01
Activity DV (U/mg)	0.10 ± 0.01	0.65 ± 0.01	0.16 ± 0.01	0.10 ± 0.01	0.27 ± 0.01	0.66 ± 0.02	0.07 ± 0.009	0.61 ± 0.02	0.28 ± 0.01
Fold-difference^a^	3.0	3.0	2.1	1.2	1.6	1.7	9.8	1.1	1.1

a Fold-difference between the activity determined for MV- and DV-Pchlide.

The plant and cyanobacterial enzymes showed activities between 0.02 U mg^−1^ (*At*LPORC) and 0.65 U mg^−1^ (*Te*LPOR), whereas the activity of the AAPB LPORs similarly varied between 0.09 U mg^−1^ (*Ds*LPOR) and 0.69 U mg^−1^ (*Eb*LPOR). The same holds for their pH-activity optimum, with all LPORs showing similar optima between pH = 7.0 and 9.0, and their thermostability (see supplementary sections 1.3.1 and 1.3.2, Supplementary Material online), where all LPORs, with the exception of the thermophilic *Te*LPOR enzyme, possessed melting temperatures between ∼35 and ∼55 °C.

For the pH- and temperature-optimum range, AAPB LPORs, with a few exceptions, showed a broader 80% optimum range compared with the plant and cyanobacterial LPORs (table 1, supplementary figs. S6 and S7 and tables S5 and S6, supplementary sections 1.3.1 and 1.3.2, Supplementary Material online). For the *K*_d_ of the ternary NADPH/Pchlide/LPOR complex, all AAPB LPORs possess higher *K*_d_ values (between ∼6 and ∼130 µM) as compared with the cyanobacterial *Te*LPOR and the plant *At*LPORC (∼2 µM for both enzymes) (table 1, supplementary figs. S9 and S10; supplementary section 1.3.3, Supplementary Material online). For both *Te*LPOR and *At*LPORC as well as for *Sp*LPOR and *Lf*LPOR, no influence of DTT on the *K*_d_ was observed (table 1, supplementary table S8, Supplementary Material online, compare *K*_d_ (+DTT/−DTT), supplementary figs. S9 and S10, Supplementary Material online). However, other AAPB LPORs either showed higher (*Gp*LPOR, *Ds*LPOR) or lower (*El*LPOR, *Eb*LPOR) *K*_d_ values in the presence of DTT.

With regard to their substrate preference for MV- or DV-Pchlide, the plant and cyanobacterial LPOR enzymes showed no preference in terms of the specific activity determined for MV- or DV-Pchlide as substrate. We consider a variant to show a preference if the fold-difference between the respective activities is >1.5. In contrast, all AAPB LPORs, with the exception of *Gp*LPOR, displayed clear preferences, with *El*LPOR and *Pd*LPOR favoring MV- over DV-Pchlide, whereas *Eb*LPOR, *Sp*LPOR, *Lf*LPOR, and *Ds*LPOR seem to favor DV-Pchlide (table 1, supplementary table S9 and fig. S13, Supplementary Material online). A detailed discussion of those observations can be found in supplementary section 1.3.4, Supplementary Material online.

In summary, AAPB LPORs are similar to cyanobacterial and plant LPORs with respect to catalytic activity, pH-activity optimum, and thermostability. However, there are notable differences in pH- and temperature-optimum ranges (broader ranges for AAPB LPORs), *K*_d_ of the ternary NADPH/Pchlide/LPOR complex (higher values for AAPB LPORs) and variation of *K*_d_ in the presence of DTT, compared with no apparent differences for cyanobacterial and plant LPORs. Moreover, in contrast to *At*LPORC and *Te*LPOR, AAPB LPORs showed a substrate preference for MV- or DV-Pchlide. Interestingly, some of the analyzed LPORs showed higher (*El*LPOR, *Pd*LPOR, *Sp*LPOR) or lower (*At*LPOR, *Lf*LPOR) specific activities, when their activity was measured using a mixture of MV-/DV-Pchlide as compared with the same measurement performed with the two separate substrates (see table 1; compare specific activity and activity MV and activity DV). This might hint at activating or inhibitory effects caused by the MV/DV substrate mixture, but exploring these intriguing features in more detail is outside the scope of the current work and merit further study. Analyzing more cyanobacterial and plant LPORs will provide more information about the variation in their biochemical properties across different species and could solidify the findings that the observed variation in AAPB LPORs is indeed intrinsic to these enzymes.

### AAPB LPORs and Cyanobacterial LPORs Possess a Conserved Structure

To identify whether AABP LPORs show a globally conserved structure when compared with cyanobacterial LPORs, we elucidated the solution structure of the apo form (protein without NADPH and Pchlide) of two AAPB LPORs (*Ds*LPOR and *El*LPOR) in comparison to the cyanobacterial *Te*LPOR (Schneidewind et al. 2019) enzyme by using small-angle X-ray scattering (SAXS). SAXS allows the structural characterization of biomacromolecules such as proteins in solution. In contrast to X-ray diffraction experiments performed on protein crystals, SAXS does not provide information on atomic coordinates. It is hence described as a low-resolution technique that is capable of providing high-precision information with respect to size and shape of the studied molecule (Neylon 2008; Jacques and Trewhella 2010). SAXS hereby also provides information about the oligomeric state of the studied protein and allows the computational reconstruction of their low-resolution shape as ab initio models.

**Fig. 4.**
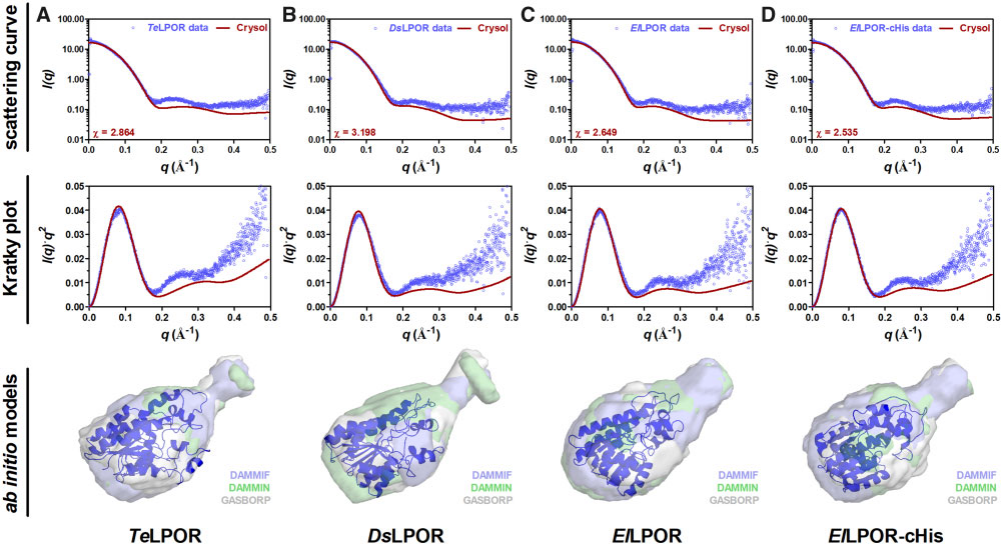
SAXS analyses of proteobacterial and cyanobacterial LPORs in solution. SAXS data (scattering curve and Kratky plot) as well as the corresponding ab initio models are shown for (*A*) *Te*LPOR, (*B*) *Ds*LPOR, (*C*) *El*LPOR, and (*D*) *El*LPOR-cHis. Experimental SAXS data are shown as blue circles superimposed with the theoretical scattering curves (red line) calculated from the respective homology model using CRYSOL (Svergun et al. 1995). Ab initio models computed by DAMMIF (Franke and Svergun 2009) (blue), DAMMIN (Svergun 1999) (green), and GASBORP (Svergun et al. 2001) (white) are shown as transparent surface superimposed with the homology model generated for the respective LPOR (in cartoon representation).

All studied LPOR samples contained an N-terminal, 20 amino acids long, His-tag. To rule out that the flexible N-terminal His-tag contributes to the obtained ab initio models, we also included an *El*LPOR sample, which possessed an eight-amino acid-long His-tag at the C-terminus instead of the N-terminus (*El*LPOR-cHis). The corresponding molecular masses estimated from the SAXS data (table 2) are in good agreement with the theoretical molecular masses, indicating that all studied apo LPORs are monomeric, as also previously shown for *Te*LPOR (Schneidewind et al. 2019). To gain a better understanding of their solution structure, we compared our SAXS data with different *Ds*LPOR, *El*LPOR, and *Te*LPOR homology models (see supplementary section 1.5, Supplementary Material online, for detailed discussion) (fig. 4, upper and middle panels). In addition, the obtained homology models were superimposed to the corresponding ab initio models, which represent the basic shape of the molecule directly computed from the SAXS data (fig. 4, lower panels; see Materials and Methods for details). As previously shown for *Te*LPOR (Schneidewind et al. 2019), the overall shape of the molecule resembles a bowling-pin appearing to consist of a larger and smaller subdomain (fig. 4, lower panels) that well accommodate the corresponding LPOR homology models. Taken together, the presented SAXS analyses provide a glimpse on the structural organization of AAPB LPORs and suggest that the newly discovered AAPB LPOR enzymes and prototypical cyanobacterial LPORs possess a conserved global structure.

**Table 2.** Molecular Mass and Radius of Gyration of the Studied LPOR Proteins Determined from SAXS.

	Molecular Mass (kDa)			*Rg* (A)	
Protein	**Porod Volume** ^a^	**Ab initio** ^b^	**Theoretical** ^c^	**Guinier** ^d^	**Crysol** ^e^
*Ds*LPOR	43.176	39.970	37.265	23.86	21.56
*El*LPOR	41.264	36.278	37.847	23.39	21.35
*El*LPOR-cHis	37.705	36.288	36.749	22.38	20.85
*Te*LPOR	38.664	31.082	38.014	22.99	20.71

a Determined from the Porod volumes as described in the Materials and Methods section.

b Calculated from the average excluded volume of the averaged filtered model calculated by the program DAMMIN.

c Determined from the amino acid sequence using the Protparam (www.web.expasy.org/protparam/) web service; last accessed September 17, 2020.

d Determined using the Guinier approximation employing AUTORG.

e Determined from the respective homology model using CRYSOL.

### Phylogeny and Evolution of the LPOR System

Phylogenetic studies to elucidate the evolution of LPOR among plants and cyanobacteria suggested that plant LPORs were obtained by endosymbiotic gene transfer from cyanobacteria (Yang and Cheng 2004; Sousa et al. 2013). Based on the previous assumption that LPORs are absent in anoxygenic phototrophs, along with the observed oxygen-sensitivity of the DPOR system, Yamazaki et al. (2006) argued that LPORs first evolved in cyanobacteria at around the time of the great oxygenation event, that is, as a consequence of increased atmospheric oxygen levels. The authors reasoned that the altered environmental conditions would compromise DPOR function and hence provide the selective pressure for the development of the oxygen-insensitive LPOR enzyme system (Yamazaki et al. 2006). Previously, we attributed the presence of LPOR in *D. shibae* to a single horizontal gene transfer (HGT) event from cyanobacteria. The here presented widespread distribution of LPORs outside of cyanobacteria challenges this hypothesis, resulting in the need to reconsider the emergence and evolution of LPORs.

For phylogenetic analysis, we used 33 different AAPB LPORs plus 203 cyanobacterial LPORs (see supplementary section 1.4.1 and table S10, Supplementary Material online). Phylogenetic trees were reconstructed by performing 50 independent runs with RAxML (Stamatakis 2014) and IQ-TREE (Nguyen et al. 2015), resulting in 100 topologically different trees. According to the approximately unbiased tree test (Shimodaira 2002), none of the 100 trees was significantly worse than the others.

**Fig. 5.**
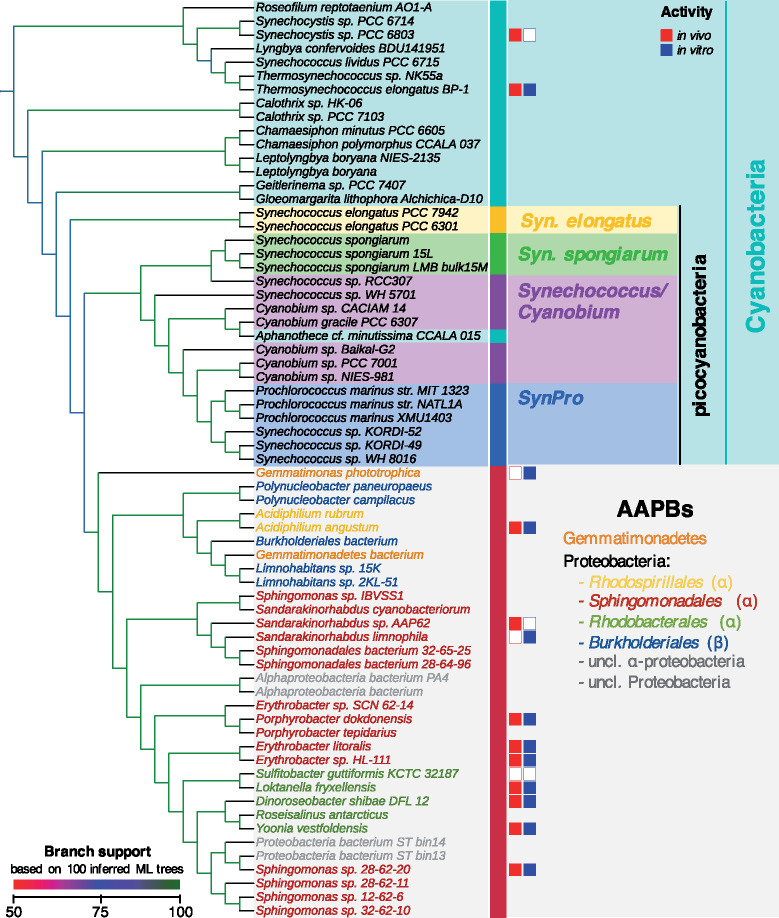
A subtree of the majority rule consensus LPOR tree displaying the position of the AAPB-derived sequences within cyanobacteria. The branches are colored according to the number of occurrences of the corresponding clade in 100 inferred trees. The activity of tested LPORs was mapped onto the subtree. The activity of LPORs is marked with filled/empty squares, that is, active/not active in vivo (in red) and in vitro (in blue).

The majority rule consensus tree (supplementary fig. S14*A*, Supplementary Material online) indicates that the 33 AAPB LPORs constitute a monophyletic group (called AAPB-clade) in all 100 trees (see fig. 5). Moreover, despite multiple multifurcations within cyanobacteria (supplementary fig. S14*A*, Supplementary Material online), the AAPB-clade clusters in 83 out of 100 trees (including the tree with the highest likelihood among 100 trees, supplementary fig. S14*B*, Supplementary Material online) with a group of picocyanobacteria, comprised by *Cyanobium*, *Synechococcus*, and *Prochlorococcus* species (see fig. 5). This placement of the AAPB-clade is quite intriguing, as there is multiple evidence of HGT from proteobacteria to picocyanobacteria (Badger and Price 2003; Beiko et al. 2005; Dvornyk 2006; Zhaxybayeva et al. 2006; Marin et al. 2007; Sousa et al. 2013), implying that the transfer between these groups is easily possible and frequent. The monophyly of AAPB-clade as well as its position within picocyanobacteria were also confirmed by trees inferred using TNT (Goloboff and Catalano 2016; parsimony), MEGA (Kumar et al. 2012; neighbor-joining), and MrBayes (Ronquist et al. 2012; Bayesian phylogeny) (supplementary fig. S17, Supplementary Material online) and additional tree topology tests (supplementary fig. S18, Supplementary Material online); albeit with the latter not providing perfect support (see Supplementary Material section 1.4.5, Supplementary Material online, for details). Thus, we consider LPOR as an example of HGT in the other direction from picocyanobacteria to AAPB proteobacteria. Based on the monophyly of the AAPB-clade within cyanobacteria, we assume that there was one HGT from cyanobacteria to AAPBs.

We now want to trace the sequence of events that led to the distribution of LPORs within AAPBs. The tree in figure 5 suggests three HGT scenarios:

(H1) acquisition of LPOR before the split of α- and β-proteobacteria;
(H2) HGT from cyanobacteria to α-proteobacteria and subsequently from α- to β-proteobacteria; and
(H3) HGT from cyanobacteria to β-proteobacteria and subsequently from β- to α-proteobacteria.

**Fig. 6.**
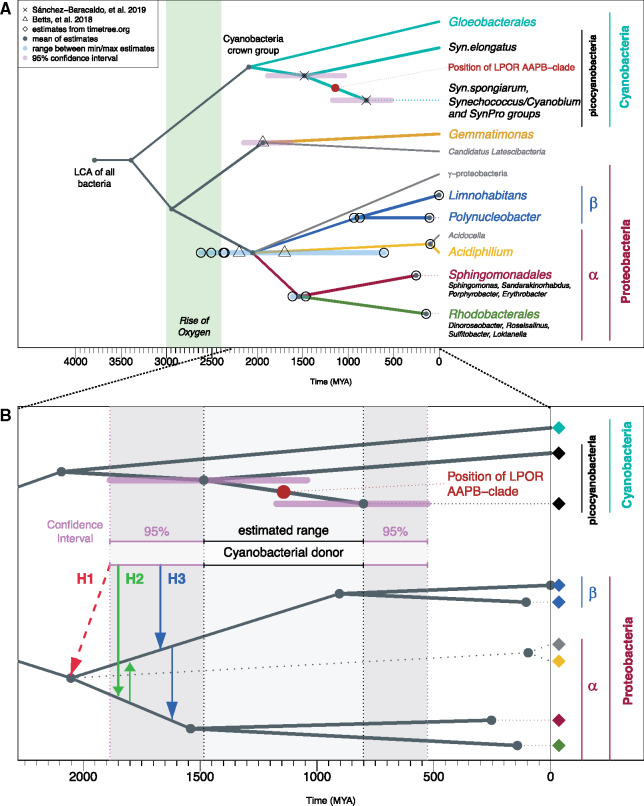
(*A*) The dated tree for the considered taxonomic units. The ranges in blue mark differences between minimum and maximum estimates for the corresponding nodes, when more than one estimate is available. The ranges in pink correspond to 95% confidence intervals as reported in the original article (see supplementary table S11, Supplementary Material online). Taxonomic units in gray do not possess a known LPOR. We mapped the position of AAPB-clade in the LPOR tree onto dated tree (indicated by the red circle). (*B*) Zoomed view of the time frame and lineages relevant for the discussion of HGT from cyanobacteria to AAPB-proteobacteria. The estimated time frame for cyanobacterial donor is between the divergence estimates of *Synechococcus elongatus* from other cyanobacteria and the divergence of other picocyanobacteria (light grey range). The 95% confidence intervals of divergence estimates are marked with the dark gray ranges. The colors of the diamonds correspond to the species in (*A*). Three alternative hypotheses are marked by the red, green, and blue arrows. The earliest emergence of cyanobacterial donor is more recent than the mean divergence estimate of α/β-proteobacteria, ruling out hypothesis of HGT from cyanobacteria to the stem of α/β-proteobacteria (H1, marked with red dashed arrow). According to the time frame, HGT from cyanobacteria to α and subsequently to β (H2, green arrows) is feasible. Also an alternative explanation: HGT from cyanobacteria to β and subsequently to α (H3, blue arrows) is feasible.

To decide which scenario is likely we considered splitting times provided by timetree.org database (Kumar et al. 2017) as well as additional divergence times from four recent studies (Shih et al. 2017; Betts et al. 2018; Magnabosco et al. 2018; Sanchez-Baracaldo et al. 2019) (fig. 6, for all estimates see supplementary table S11, Supplementary Material online).

According to the position of the AAPB-clade within picocyanobacteria, the presumable HGT from cyanobacteria to AAPBs happened between the split of *Synechococcus elongatus* from other cyanobacteria (1,484 Ma; 1 Ma = 1 million years ago) and the divergence of other picocyanobacteria (801 Ma) (the corresponding range with 95% confidence interval is marked in fig. 6*B*).

The mean of estimates for the divergence of α-/β-/γ-proteobacteria suggests that their split is much older than the earliest emergence of the potential cyanobacterial donor (fig. 6*B*, red arrow), which rules out the hypothesis H1 of HGT to the ancestor of α-/β-proteobacteria. The time divergence estimates for α-proteobacteria with LPORs (orders *Rhodobacterales* and *Sphingomonadales*) and β-proteobacteria (*Burkholderiales* order: genera *Limnohabitans* and *Polynucleobacter*) allow for HGT from cyanobacteria to the respective ancestral lineages (fig. 6*B*, green and blue arrows). Therefore, according to the timeline hypotheses H2 and H3 are possible.

Nevertheless, note, that the split of β-proteobacteria genera is much younger than the divergence of α-proteobacteria orders with LPORs. Therefore, if LPORs were transferred from cyanobacteria to β-proteobacteria and then from β to α we would expect more LPORs in β-proteobacteria. However, the opposite is observed, thus, favoring the hypothesis H2 of HGT from cyanobacteria to α-proteobacteria and subsequently from α to β.

The transfer of LPOR from α- to β-proteobacteria also corroborates evolutionary studies on other photosynthetic genes, suggesting that β-proteobacteria obtained their photosynthetic apparatus from α-proteobacteria (Igarashi et al. 2001; Nagashima S and Nagashima KVP 2013; Imhoff et al. 2017, 2019). For example, in the study by Imhoff et al. (2019) on proteins of RC type II (PufHLM) and key enzymes of Bchl biosynthesis (BchXYZ) β-proteobacteria (*Burkholderiales* and *Rhodocyclales*) cluster within α-proteobacteria (*Rhodospirillales* and *Rhizobiales*). In the LPOR tree (fig. 5), two *Rhodospirillales* from *Acidiphilium* genus cluster within *Burkholderiales* (β). It is possible that if additional LPOR sequences are identified in *Rhodospirillales* and/or in *Rhizobiales* the position of *Acidiphilium* species might change to resemble that of the PufHLM and BchXYZ proteins. Taking the findings from photosynthetic genes into account, it seems more plausible that LPOR in β-proteobacteria (*Burkholderiales*) was obtained via HGT from α-proteobacteria; probably together with other photosynthetic genes via a single HGT.

Finally, we point out that LPOR phylogeny for α-proteobacteria resembles the PufHLM–BchXYZ phylogeny and 16S rRNA tree (Imhoff et al. 2019). Therefore, the HGT from cyanobacteria to α-proteobacteria should have occurred before the split of *Rhodobacterales* and *Sphingomonadales*.

The LPOR of *Gemmatimonadetes* bacterium clusters within β-proteobacterial LPORs as a sister of *Limnohabitans*. At the time of revision of the manuscript, we identified an LPOR in *Gemmatimonas* sp. TET16 (GenBank accession number WP_171227737.1), which clustered with *Gemmatimonadetes* bacterium within β-proteobacteria (data not shown). This suggests an HGT from β-proteobacteria to *Gemmatimonadetes*. The basal placement of *G. phototrophica* in the AAPB-clade (outside of α- and β-proteobacteria) seems to complicate the explanation. However, Zeng et al. (2014) showed that the phylogenetic position of *G. phototrophica* is inconsistent. For photosynthetic genes encoding AcsF and BchIDH enzymes, the same basal placement as in the LPOR tree was observed. On the other hand, BchLNB from *G. phototrophica* clusters within proteobacteria. Moreover, also in the PufHLM–BchXYZ phylogeny (Imhoff et al. 2019) *G. phototrophica* clusters basal to *Burkholderiales* (β) within *Rhizobiales* (α). It was also shown that photosynthetic gene cluster of *G. phototrophica* resembles that of β-proteobacteria *Rubrivivax gelatinosus* IL144 (Zeng et al. 2014). Summarizing, LPORs of *Gemmatimonadetes* species show high similarity to β-proteobacterial LPORs. *Gemmatimonadetes* bacterium and the newly identified LPOR of *Gemmatimonas* sp. TET16 clustered within β-proteobacteria. The positioning of *G. phototrophica* remains unclear, until more data become available. The position of *Gemmatimonadetes* species in LPOR tree (fig. 5) suggests β-proteobacteria as donor for the HGT and, in principle, is in concordance with the published phylogenies on other photosynthetic genes.

Overall, our analysis suggests that AAPBs originally acquired LPORs via HGT from cyanobacteria to proteobacteria with the donor likely being an ancestral picocyanobacterium. Given multiple evidence of HGT between picocyanobacteria and proteobacteria (Badger and Price 2003; Beiko et al. 2005; Dvornyk 2006; Zhaxybayeva et al. 2006; Marin et al. 2007; Sousa et al. 2013), this transfer event seems very likely. Taking into account evolutionary studies on other photosynthetic genes and also the evolutionary timeline, we suggest a cascade of HGT: cyanobacteria transferred LPOR genes to α-proteobacteria; then α-proteobacteria transferred it to β-proteobacteria; finally, β-proteobacteria transferred LPOR genes to *Gemmatimonadetes*. This scenario is in accordance with the suggested transfer of photosynthetic apparatus from α- to β-proteobacteria and to *Gemmatimonadetes*. Identifying more LPORs in nonoxygenic phototrophic bacteria is needed to solidify our findings and to support the idea of HGT cascade.

### Concluding Remarks: Potential Origin, Evolution, and Function of AAPB LPORs

From our phylogenetic analysis, we conclude that AAPB LPORs were most likely originally transferred from ancestral picocyanobacteria to α-proteobacteria. Picocyanobacteria are the smallest cyanobacteria, ubiquitous in freshwater, brackish, and marine environments. In freshwater, mainly *Synechococcus*, *Cyanobium*, and *Synechocystis* genera are found, whereas marine habitats are dominated by *Synechococcus* and *Prochlorococcus* (Jasser and Callieri 2016). Numerous examples of HGT between picocyanobacteria and proteobacteria have been previously reported (Badger and Price 2003; Beiko et al. 2005; Dvornyk 2006; Zhaxybayeva et al. 2006; Marin et al. 2007; Sousa et al. 2013), providing conclusive evidence, that HGT between these bacterial groups is feasible and frequent.

Further, for six AAPBs with biochemically characterized LPORs (*El*LPOR, *Pd*LPOR, *Gp*LPOR, *Sp*LPOR, *Lf*LPOR, and *Ds*LPOR) we related LPOR biochemical properties with the growth characteristics of the host and their evolution. Habitat, site of isolation, and growth characteristics with regard to pH and temperature (if known) are summarized in supplementary table S16, Supplementary Material online. All AAPBs were isolated from aquatic habitats such as freshwater lakes and marine habitats. Some of the organism were isolated from cyanobacterial/microbial mats (*E. litoralis*, Loktanella *fryxellensis*) indicating close association with cyanobacteria. The LPOR-containing AAPBs thus seem to dwell in an environment that is commonly rich in picocyanobacteria. It is hence tempting to speculate that aquatic environments, in which environmental conditions such as light availability, oxygen levels, temperature, salinity and pH affect the growth, survival and productivity of the corresponding organisms, have contributed to the transfer of LPOR. The presence of an LPOR would hereby provide its AAPB host with a selective advantage as it would enable the organism to enhance Bchl synthesis under aerobic conditions. This adaptability is also reflected in the broad pH- and temperature-optima ranges observed for AAPB LPORs (table 1, fig. 3, supplementary figs. S6 and S7, Supplementary Material online) as well as their hosts (supplementary table S16, Supplementary Material online). In conclusion, further studies of AAPB LPORs and their hosts are needed to address the intriguing role of this unique enzyme for AAPBs.

## Materials and Methods

### Identification of LPORs

To search for putative LPORs, we analyzed all publicly available bacterial genomes in GenBank (Clark et al. 2016) (as of July 17, 2018) using the HMMER software (http://hmmer.org/, last accessed September 16, 2020, version 3.1b2) together with an LPOR HMModel from TIGRFAMs (Haft et al. 2003) (all HMModels are listed in supplementary table S2, Supplementary Material online). Additionally, LPORs, whose biochemical properties were previously characterized, were included in the analysis for a comparison. Supplementary table S1, Supplementary Material online, displays the taxonomic information, presence/absence analysis for marker genes as well as accession numbers for all putative LPORs in AAPBs and for the known reference LPORs. Accession numbers for all bacterial LPORs used for phylogenetic tree inference are provided in supplementary table S10, Supplementary Material online.

### Bacterial Strains and Culture Conditions

All strains used in this study are listed in supplementary table S3, Supplementary Material online. *Escherichia coli* strains DH5α (Invitrogen) and S17-1 (Simon et al. 1983) used for cloning and conjugation were grown in lysogeny broth (LB) medium (Carl Roth, Arlesheim, Switzerland) at 37 °C, under constant agitation (130 rpm). Heterologous expression of all LPOR encoding genes was performed using *E. coli* BL21(DE3) employing different media (see below). Antibiotics were added to *E. coli* culture medium in the following final concentrations (µg ml^−1^): 100 (Ap, ampicillin), 50 (Km, kanamycin). Plasmid DNA was introduced into *E. coli* using heat-shock transformation (Swords 2003). The *R. capsulatus* wild-type strain B10S, a spontaneous streptomycin-resistant mutant of the strain B10 (Klipp et al. 1988), and recombinant mutant strains were cultivated on Peptone Yeast agar plates (Klipp et al. 1988) containing 2% (w/v) Select Agar (Thermo Fisher Scientific) or in RCV liquid medium (Weaver et al. 1975) supplemented with 15 mM ammonia at 30 °C. Chemoorganotrophic cultivation was carried out in Erlenmeyer flasks filled with 50-ml RCV medium under permanent shaking (130 rpm) in the dark. For photoheterotrophic cultivation, capped air-tight reaction tubes were filled with 15-ml RCV cultivation medium to create an oxygen-free atmosphere. The media were supplemented with 200 µg ml^−1^ streptomycin for the *R. capsulatus* wild-type strain B10S, with 200 µg ml^−1^ streptomycin and 10 µg ml^−1^ spectinomycin for the DPOR-deficient *R. capsulatus* ΔbchB strain (Kaschner et al. 2014) and or with 200 µg ml^−1^ streptomycin and 25 µg ml^−1^ kanamycin for the DPOR-deficient *R. capsulatus* ΔbchB strains containing the LPOR expression plasmids (see below). The respective cultures were constantly illuminated with bulb light (6 × 60 W). Plasmids were introduced into the DPOR-deficient *R. capsulatus* ΔbchB by conjugational transfer using *E. coli* S17-1 as donor strain as described before by Klipp et al. (1988). *Rhodobacter capsulatus* ZY5 (Yang and Bauer 1990), which was used for production and purification of the Pchlide substrates, was cultivated in VN-Medium (10 g/l yeast extract, 5.7 µM K_2_HPO_4_, 2 µM MgSO_4_ pH = 7.0) supplemented with 25 µg ml^−1^ Rifampicin in the dark at 30 °C under constant agitation (130 rpm). Production cultures were inoculated to an OD_660nm_ of 0.01 and grown under microaerobic conditions (culture volume 50% of the flask volume; nonbaffled Erlenmeyer flasks).

### Construction of Expression Plasmids

All putative AAPB LPOR encoding genes, as well as the genes coding for the LPORs with proven activity, were synthesized by Life Technologies (ThermoFisher Scientific GmbH, Bonn, Germany) (*Sp*LPOR, *Eb*LPOR, *Pd*LPOR, *El*LPOR, *Lf*LPOR, *Gp*LPOR, *An*LPOR, *Sa*LPOR, *Te*LPOR), MWG Eurofins (Ebersberg, Germany) (*Hv*LPORA, *Ss*LPOR, *Sg*LPOR), or ATUM (Newark, CA) (*Sl*LPOR, *Yv*LPOR, *Se*SDR, *At*LPORC) with adapted codon-usage for the expression in *E. coli* and *R. capsulatus.* An overview over the cloning strategy is given in supplementary table S3, Supplementary Material online. All synthesized genes were subcloned from the respective synthesis vector into pET28a (Merck, Darmstadt, Germany) and pRhokHi-2 (Katzke et al. 2010) for expression in *E. coli* and *R. capsulatus*, respectively. The gene coding for the LPOR from *D. shibae* (*Ds*LPOR) was PCR amplified from *D. shibae* genomic DNA and cloned as described previously (Kaschner et al. 2014). All LPORs were appended with an N- or C-terminal hexa-histidin-tag encoded by the expression vector (see supplementary table S3, Supplementary Material online). All final constructs were verified by sequencing (SeqLab, Göttingen, Germany).

### Heterologous Production and Purification of LPORs

For the heterologous production and purification of all LPORs, the respective genes were expressed in *E. coli* BL21(DE3). Plasmid containing *E. coli* BL21(DE3) cells were grown in either LB (Carl Roth, Karlsruhe, Germany; Green et al. 2012) (*Ds*LPOR), terrific broth (Carl Roth, Karlsruhe, Germany; Green et al. 2012) (*Hv*LPORA, *Sl*LPOR, *Gp*LPOR), or modified autoinduction media (Studier 2005) (*An*LPOR, *Eb*LPOR, *El*LPOR, *Lf*LPOR, *Yv*LPOR, *Pd*LPOR, *Sa*LPOR, *Se*SDR, *Sg*LPOR, *Sp*LPOR, *At*LPORC, SsLPOR, *Te*LPOR) supplemented with kanamycin (50 µg ml^−1^) for plasmid maintenance (500 ml culture volume in 5-l nonbaffled Erlenmeyer flasks). LB and terrific broth cultures were grown at 37 °C (250 rpm) until an OD_600nm_ of 1 was reached. Subsequently, gene expression was induced by addition of 0.4 mM isopropyl β-d-1-thiogalactopyranoside, the temperature was reduced to 25 °C, and the cultures were grown for additional 2 h under constant agitation (250 rpm). All cultures were grown initially for 2 h at 37 °C under constant agitation (250 rpm), subsequently the temperature was decreased to 15 °C and cells were grown for additional 48 h (*Te*LPOR, *El*LPOR, *An*LPOR, *Ss*LPOR, *Sg*LPOR, *Yv*LPOR, *Sp*LPOR, *Lf*LPOR, *Pd*LPOR, *Sa*LPOR, *Se*SDR) or 72 h (*At*LPORC, *Eb*LPOR).

After heterologous expression, cells were harvested by centrifugation (30 min, 6,750 × g, 4 °C) and resuspended in buffer (20 mM Tris/HCl, 500 mM NaCl, 20% [w/v] glycerol, pH = 7.5). The corresponding cell suspensions (10% [w/v] wet cells) were disrupted by passing the cell suspension five times through an EmulsiFlex-C5 high-pressure homogenizer (AVESTIN, Ottawa, ON, Canada) at a pressure of 1,000 bar. All LPOR proteins were purified by immobilized metal ion affinity chromatography (IMAC) as described previously (Kaschner et al. 2014). After IMAC, samples were desalted by size exclusion chromatography (SEC) using a Sephadex G25 column (560 ml column volume [CV], XK50/30, GE Healthcare Life Science, VWR International GmbH, Langenfeld, Germany). Protein samples were concentrated to a concentration of at least 1 mg ml^−1^ using Nanosep Centrifugal Device concentrator (molecular weight cutoff 10,000 Da) (Pall, Germany) and further purified by preparative size-exclusion chromatography using a Superdex 200 (XK16/60, GE Healthcare Life Science, VWR International GmbH) column with 20 mM Tris/HCl buffer pH = 7.5, supplemented with 500 mM NaCl and 20% glycerol, es eluent. Final samples were concentrated, flash frozen in liquid nitrogen, and stored in the same buffer at −20 °C until further use.

### Sample Preparation for SAXS

Apo-protein samples of *Te*LPOR, *Ds*LPOR, *El*LPOR, and *El*LPOR-cHis (control sample with C-terminal His_6_-tag) were prepared by concentrating a dilute (0.5 mg ml^−1^), freshly prepared IMAC and SEC purified protein sample using a Nanosep centrifugal concentrator (molecular weight cutoff 10,000 Da) (Pall, VWR International GmBH, Darmstadt, Germany). Samples of defined concentration were removed during concentration and the corresponding flow-through was used as buffer reference for SAXS measurements. All SAXS samples were centrifuged at 4 °C for 20 min at 21,000 × g to remove larger aggregates and particulate material. Protein concentrations were determined by measuring protein absorption at 280 nm using a NanoDrop 2000c spectral photometer (ThermoFisher Scientific GmbH). Molar extinction coefficients (ε) were determined based on amino acid composition using the ProtParam web service (https://web.expasy.org/protparam; last accessed September 17, 2020) (Gasteiger et al. 2005). The following extinction coefficients were used: *Ds*LPOR: ε_280nm_ = 36,690 M^−1 ^cm^−1^; *El*LPOR: ε_280nm_ = 42,190 M^−1 ^cm^−1^; *El*LPOR-cHis: ε_280nm_ = 42,190 M^−1 ^cm^−1^; *Te*LPOR: ε_280nm_ = 35,660 M^−1 ^cm^−1^.

### Pchlide Production and Purification

Pchlide was produced using *R. capsulatus* ZY5 (Yang and Bauer 1990) in which the *bchL* gene encoding for one subunit of the DPOR was deleted. This strain therefore accumulates Pchlide, as a mixture of MV- and DV-Pchlide (Heyes et al. 2006). The strain was grown as described above, and the secreted Pchlide was adsorbed to hydrophobic polyurethane cubes (edge length 1 cm), which were added to the cultures during cultivation. After 24–36 h, the cubes were removed and cells washed off with tricine buffer (10 mM tricine pH = 7.5). Subsequently, Pchlide was extracted from the cubes with 100% methanol and filtrated (glass fiber filter and cellulose acetate filter, pore size 0.8 and 0.45 µm). Pchlide was purified by column chromatography using an ÄKTAbasic FPLC system (GE Healthcare, Solingen, Germany) using C-18 solid-phase extraction (SPE) material (Sep-Pak, Waters, Milford, MA) filled into an ECO^PLUS^SR TAC15/500LGO-SR-2 column (75 ml CV) (YMC Europe GmbH, Dinslaken, Germany). To facilitate binding, the filtrated Pchlide extract was diluted to a final concentration of 40% (v/v) methanol with tricine buffer. The SPE column was equilibrated with methanol:tricine buffer (40:60-Vol%), the Pchlide extract was loaded, and the column was washed using the same methanol:tricine buffer mixture. To separate carotenoids and other nonwanted pigments from Pchlide, the methanol concentration was increased stepwise to 50% (after two CV) and 60% (after 25 CV). Finally, Pchlide was eluted using a methanol:tricine buffer ratio of 75:25-Vol%. The obtained purified Pchlide eluate was diluted with tricine buffer to a final methanol concentration of ∼25%. Subsequently, Pchlide was extracted by liquid–liquid extraction using diethyl ether. The resulting Pchlide extract (in diethyl ether) was dried with MgSO_4_, the ether was evaporated using a rotary evaporator (Rotavapor R-100, Büchi, Flawil, Switzerland), and the dried sample was stored under argon atmosphere at −20 °C in the dark.

### HPLC-Photodiode Array Detector and MS Analysis of *R. capsulatus* Purified Pchlide

The Pchlide preparation purified from *R. capsulatus* ZY5, which consists of a mixture of MV- and DV-Pchlide (Heyes et al. 2006), was analyzed by high-performance liquid chromatography (HPLC) in cooperation with Dr Klaus Bollig at the Shimadzu Laborwelt (Duisburg, Germany). The dried Pchlide preparation was dissolved in 100% methanol. MV- and DV-Pchlide were first separated liquid chromatography (LC-10Ai series; Shimadzu Deutschland GmbH, Duisburg, Germany, equipped with a SPDM10Avp photodiode array detector). Chromatographic separation was performed with an analytical C30 column (ISAspher 200-5 C30-CXT, 4.6 mm × 250 mm, ISERA GmbH, Düren, Germany) using a binary mobile phase (A: 5 mM ammonium acetate buffer pH = 6, 30% methanol, B: 100% methanol) in a gradient program (0–2 min: 5% A, 95% B; 25–45 min: 0% A, 100% B; 45–50 min: 5% A, 95% B). A constant flow rate of 1 ml min^−1^ was used. Elution was monitored at 440 nm and absorption spectra were recorded for each elution peak. Identification of the eluting Pchlide species was achieved by mass spectrometry (MS) at the Shimadzu Laborwelt (Duisburg, Germany) using an HPLC-coupled hybrid ion trap-time of flight mass spectrometer (LCMS-IT-TOF, Shimadzu, Duisburg, Germany). Electrospray ionization was used and the resulting ions were further fragmented in the ion trap using Argon as collision gas. The ion accumulation time in the octopole was 20 ms (MS^1^ mode) and 40 ms (MS^2^ mode). Mass spectra were acquired in positive ionization mode in the range of 150–1,000 *m*/*z*.

### Purification of MV- and DV-Pchlide

Pchlide was prepared from *R. capsulatus* ZY5 by solid-state extraction as described above. MV- and DV-Pchlide were separated using a preparative C30 HPLC column (ISAspher 200-5 C30-CXT, 20 mm × 250 mm, ISERA GmbH). An ÄKTAbasic FPLC system (GE Healthcare Life Science, Freiburg, Germany) was adapted for the use of organic solvents by employing polyether ether ketone fittings and tubes. The system was equipped with a column oven (Gynkotek STH 585, Gynkotek, Dionex, Thermo Fisher Scientific GmbH). which was set to 35 °C. Per run 8 ml of the *R. capuslatus* ZY-derived Pchlide, dissolved in 100% methanol, was loaded onto the column. Separation of MV- and DV-Pchlide was achieved using a binary mobile phase (A: 5 mM ammonium acetate buffer pH = 6, 30% methanol, B: 100% methanol) by employing a gradient program (0–2 min: 5% A, 95% B; 25–45 min: 0% A, 100% B; 45–50 min: 5% A, 95% B) at a constant flow rate of 15 ml min^−1^. Elution was monitored by continuously measuring the absorbance of the soret band of MV- and DV-Pchlide at 450 nm. The MV- and DV- Pchlide-containing fractions were pooled, diluted to a final methanol concentration of <25% with 10 mM tricine buffer (pH = 7.5), and extracted by liquid–liquid extraction into dry diethyl ether. The resulting MV- and DV-Pchlide extracts (in diethyl ether) were dried with MgSO_4_. The diethyl ether was subsequently evaporated using a rotary evaporator (Rotavapor R-100, Büchi), and the dried MV- and DV-Pchlide preparations were stored in the dark at −20 °C under argon atmosphere until further use.

### In Vitro LPOR Activity Assays

#### General Assay Setup

All light-dependent activity measurements were performed as previously described (Kaschner et al. 2014). In brief, protein samples (0.17 µM) or cell free lysates were diluted with assay buffer (20 mM Tris/HCl buffer [pH = 7.5] supplemented with 500 mM NaCl and 20% [v/v] glycerol, 160 µM NADPH, 70 mM DTT, and 0.03% [v/v] Triton X-100) in half-micro disposable cuvettes (1 cm light path). The purified and dried Pchlide substrate was dissolved in 100% methanol and added to the protein (lysate) buffer mixture to a final concentration of 3.5 µM (5% [v/v] methanol in the assay). Subsequently, the assay mixture was equilibrated for 5 min at 25 °C. A blue-light emitting LED (450 nm; 2.6 mW cm^−2^) was mounted on top of the cuvette, and light-dependent Pchlide turnover was achieved by illuminating the assay mixture employing cycles of 1-s blue-light illumination followed by 11 s in the dark during which an absorption spectrum from 620 to 720 nm was recorded. Weakly active samples such as those with *At*LPORC were illuminated by cycles of 6 s of illumination followed by 12 s of darkness. Pulsed illumination was achieved by using a microcontroller-controlled LED driver (Arduino UNO, Smart Projects, Italy). Data were analyzed using a home-written shell script, which filters and removes spectra that contain illumination events. LPOR activity was quantified by linear regression on the initial linear rise in absorbance of the Chlide product (672 nm) that corresponds to the initial reaction velocity. Chlide formation was quantified using molar extinction coefficient of ε_672nm_ = 69,950 M^−1 ^cm^−1^ (Klement et al. 1999; Heyes et al. 2000). One unit (U) of LPOR activity was defined as the amount of enzyme which reduces 1 µmol Pchlide to Chlide per minute under the given reaction conditions.

#### pH and Temperature Optima

All assays were carried and analyzed out as described for the general assay setup. Three different buffer systems (pH = 5.5–7.5: 200 mM sodium phosphate buffer; pH = 7.5–9.0: 200 mM Tris/HCl; pH = 9.0–10.0: 200 mM glycine buffer; all three buffers were supplemented with 500 mM NaCl and 20% [v/v] glycerol) were used to cover the pH range between pH = 5.5 and 10. LPOR temperature optima were determined for the temperature range from 10 to 55 °C in 5 °C increments. To avoid temperature-dependent pH changes during the measurement at elevated temperatures, the pH of the assay was adjusted at the respective temperature. Samples were equilibrated at the desired temperature in disposable half-micro cuvettes (1 cm light path) using a cuvette-holder equipped with a Peltier-based thermostat. The accuracy of the temperature provided by the thermostat was determined by recording the actual temperature from a buffer solution.

#### MV-/DV-Pchlide Substrate Preference

MV- and DV-Pchlide were prepared as described in section “Purification of MV- and DV-Pchlide,” and the dried powder was dissolved in 100% methanol to yield a stock solution containing at least 140 µM of the respective Pchlide species. All measurements were performed using the general assay setup as described above by using purified MV- and DV-Pchlide at a fixed concentration of 3.5 (±0.15) µM. The data were analyzed as described above.

### In Vivo LPOR Activity Assay

The in vivo LPOR activity assay was performed as described previously (Kaschner et al. 2014). To comparatively analyze the activity of chosen LPORs in vivo, the *R. capsulatus* LPOR expression strain (i.e., DPOR-deficient *ΔbchB* strain carrying the respective LPOR-encoding pRhokHi-2 derivatives, supplementary table S3, Supplementary Material online) was used to characterize its ability 1) to grow photoheterotrophically and 2) to synthesize BChl *a* (*bacteriochlorophyll a*). Increasing cell densities and BChl *a*-mediated light absorption were used as a measure for LPOR in vivo activity. To ensure that all expression cultures were in the same growth phase for the LPOR in vivo assay, *R. capsulatus* expression strains were precultivated twice under aerobic chemoheterotrophic conditions in RCV liquid medium (second culture starting with OD_660nm_ = 0.1). Cells from second precultures were used after 72 h of cultivation to inoculate the corresponding test cultures in RCV starting with an initial cell density of OD_660nm_ = 0.1. The test cultures were grown under photoheterotrophic conditions (i.e., in the absence of oxygen and under constant illumination). To monitor LPOR activities over time, increase of cell density was analyzed for 10 days at 660 nm and BChl *a* formation was investigated by recording whole-cell absorption spectra of 100-µl samples between 300 and 900 nm (using a TECAN Infinite plate reader) that were normalized to a cell density of OD_660 nm_ = 1. In addition, the color phenotypes of all test cultures were photodocumented after 240 h of cultivation. Images were taken under the same light conditions with identical camera settings including white balance.

### Determination of the Dissociation Constant of the LPOR/NADPH/Pchlide Ternary Holoprotein Complex

The dissociation constant, *K*_d_, of Pchlide binding to the ternary LPOR/NADPH/Pchlide complex was determined using a spectral method similarly to previous studies on cryptochromes (Kutta et al. 2017) and CarH photoreceptors (Kutta et al. 2015). Samples were prepared containing different concentrations of LPOR (0–200 µM) apo-protein, a constant concentration of NADPH (160 µM) and a constant concentration of Pchlide (2 μM) in buffers with and without 70 mM DTT containing 20 mM Tris/HCl, 500 mM NaCl, 20 Vol% Glycerol, and Triton X-100 (0.03 Vol%). An UV-Vis absorption spectrum over 1 cm pathlength was acquired for each concentration of apo-protein used giving the spectral evolution from the unbound Pchlide spectrum into the almost fully bound Pchlide spectrum with the typical redshift of the Q_y_ absorption band when bound to the LPOR protein pocket. This data matrix can be decomposed into its principle species spectra and corresponding mole fraction profiles. The mole fractions were determined by fitting the pure spectra of unbound and bound Pchlide to each single mixed spectrum. As the bound Pchlide spectrum is a priori unknown, it is initially estimated by the last spectrum (highest concentration of apo-protein) in the spectral sequence. Each mixed spectrum also contains an absorptive contribution due to scattered light from the sample (especially at high concentrations of apo-protein), which was accounted for by a general scatter function of the following form:

(1) $$f\left(\lambda \right)=a\lambda^{-n}+y_{0},$$

where λ is the wavelength, $y_{0}\;$accounts for the offset, a scales the curve, and n determines the curvature of the scatter function. n was fixed to 2 representing a reasonable curvature of the scatter contribution. The mole fraction profiles show a typical binding curve of the following chemical equilibrium:

(2) $$A+B\;\begin{array}{c} k_{1}\\ ⇄\\ k_{-1} \end{array}\text{AB},$$

where it is assumed NADPH binding to LPOR-apo has a much higher affinity so that the complex LPOR/NADPH can be seen as one molecule. The dissociation constant is defined as:

(3) $$K_{d}=\frac{k_{-1}}{k_{1}}=\frac{\left[A\right][B]}{\left[\text{AB}\right]}.$$

The amount of complex AB, $x_{\text{AB}}$, is dependent on [A] and [B], the concentration of the two binding partners, and is given by:

(4) $$x_{\text{AB}}([A])=S_{0}+(S_{\text{max}}-S_{0})\frac{K_{d}+\left[B\right]+\left[A\right]-\sqrt{{\left(K_{d}+\left[B\right]+[A]\right)}^{2}-4[B][A]}}{2[B]}.$$

$\left[A\right]$ is the independent variable representing the concentration of the apo-protein, [B] corresponds to the 2 μM fixed Pchlide concentration, and $S_{0}$ and $S_{\text{max}}$ represent the minimal and maximal limits of the binding curve, respectively. The mole fractions for unbound and in LPOR bound Pchlide were globally least squared fitted to a common $K_{d}$ using the Levenberg–Marquardt algorithm. The first global fit was used to determine the contamination of the unbound Pchlide spectrum in the a priori assumed bound Pchlide spectrum. Then, the initial guess spectrum and the mole fractions were corrected correspondingly yielding the pure in LPOR spectrum as well as mole fractions ranging from 0 to 1 as expected.

#### Temperature-dependent Unfolding and Thermostability

The temperature-dependent unfolding and the thermostability of LPORs were determined using differential scanning fluorimetry (DSF), which detects the unfolding of proteins by monitoring the temperature-dependent changes in the fluorescence of the aromatic amino acids of proteins. For Nano-DSF measurements, a Prometheus NT.Flex (NanoTemper Technologies GmbH, Munich, Germany) instrument was used. Purified LPOR samples (10 µl) with a concentration of 0.6 mg ml^−1^ were subjected to a linear unfolding ramp (0.5 °C min^−1^, from 15 to 85 °C). The intrinsic tryptophan fluorescence of the protein was monitored continuously (18 data points per minute) at 350 and 330 nm. Unfolding transition midpoints were determined from the first derivative of the fluorescence ratio (F350/F330) by using the RT.ThermControl Software (NanoTemper Technologies GmbH). Thermostabilities were measured at different temperatures for 30 h by using the RT.TimeControl Software (NanoTemper Technologies GmbH).

#### SAXS Measurements and Data Analysis

SAXS experiments were performed at beamline BM29 (Pernot et al. 2013) at the European Synchrotron Radiation Facility (ESRF, Grenoble, France) using 12.5 keV X-ray radiation with a wavelength of 0.9919 Å. All measurements were carried out at 10 °C. For each LPOR protein, four samples with concentrations of ∼0.5, 1, 3, and 5 mg ml^−1^ were measured in 20 mM Tris/HCl buffer pH = 7.5 supplemented with 500 mM NaCl and 20% (v/v) glycerol. The exact protein concentrations of the measured LPOR samples are listed in supplementary table S14, Supplementary Material online. The samples were continuously purged through a 1-mm quartz capillary at a flow rate of 2.3 µl s^−1^. The buffer reference was measured before and after each protein sample. For each sample/reference ten frames with an exposure time of 3 s each were recorded. Frames without radiation damage were merged. The data were scaled by the protein concentration and extrapolated to infinite dilution. Scattering data were analyzed employing the ATSAS software package (Petoukhov et al. 2012). Data obtained for low and high concentration samples were merged. Lower concentration data were used for the smaller *q*-range, whereas the data at higher concentration were used for the high *q*-range. SAXS data were inspected visually for the presence of aggregation based on the Guinier-Plot. For each sample, a low and high concentration data set was merged (see supplementary table S14, Supplementary Material online, for details). The Porod volume, calculated with the program DATAPOROD, was used to estimate the molecular mass of the scattering particle, by using a division factor of 1.7 (Petoukhov et al. 2012). The distance distribution function *P*(*r*) was determined using the program DATGNOM. Ab initio models were built employing the programs GASBORP (Svergun et al. 2001), DAMMIF (Franke and Svergun 2009), and DAMMIN (Svergun 1999). For each LPOR data set 20 ab initio models were generated, which were subsequently averaged and filtered using the program DAMAVER (Volkov and Svergun 2003). For the corresponding filtered models, the envelope function was determined using the SITUS package (Wriggers 2012). The molecular mass was estimated from the excluded volume of the filtered model by dividing the respective values by 2 (Petoukhov et al. 2012). Theoretical scattering curves for the available LPOR homology models were calculated and fitted to the experimental SAXS data using the program CRYSOL (Svergun et al. 1995). LPOR homology models were built with YASARA Structure Version 16.6.24 (Krieger and Vriend 2014, 2015) using either the previously published model of the LPOR of *Synechocystis* sp. (*Ss*LPOR) (Townley et al. 2001), recently published (*Te*LPOR of *T. elongatus* BP-1, PDB ID: 6RNV; Zhang et al. 2019), or deposited (*Ss*LPOR of *Synechocystis* sp. PCC 6803, PDB-ID: 6R48; Zhang et al. 2019) LPOR X-ray structures as templates.

### Bioinformatic Analyses and Phylogenetic Tree Inference

#### Presence/Absence Analysis

The presence/absence of marker proteins, used for the identification of potentially (aerobic) anoxygenic photosynthetic bacteria, was inferred using the HMMER software (http://hmmer.org/, last accessed September 16, 2020, version 3.1b2) together with an HMM for the corresponding proteins from either TIGRFAMs (Haft et al. 2003) (LPOR, DPOR subunits BchB, BchL, BchN; and BchE, AcsF, PufL, PufM, PsbA, PsbD) or Pfam (Finn et al. 2016) (RuBisCO, PRK, PS I, PS II). All model IDs are given in supplementary table S2, Supplementary Material online. We call a protein absent, if no hit meeting the trusted cutoff for the *E*-value was identified with the corresponding HMM. The cutoffs used are the default ones, either specified in the HMM or the software.

#### Phylogenetic Tree Reconstruction and Visualization

Multiple sequence alignments were built with MAFFT (Katoh and Toh 2008). According to Bayesian information criterion, LG+I+G is the best-fit model for our LPOR alignment (ModelFinder [Kalyaanamoorthy et al. 2017] with option -m TESTONLY). RAxML (version 8.2.10; Stamatakis 2014) and IQ-TREE (version 1.6.12; Nguyen et al. 2015) were employed for tree reconstruction. Bootstrap scores were computed using RapidBootstrap (Stamatakis et al. 2008) and UFBoot2 (Hoang et al. 2018) for the trees inferred with RAxML and IQ-TREE, respectively. To evaluate whether any of the inferred trees is significantly worse than the others, the approximately unbiased tree test (Shimodaira 2002) was used. For tree visualization, iTOL (Letunic and Bork 2016) and FigTree (Rambaut 2012) were employed. We also inferred LPOR trees with alternative methods using TNT (v.1.5 [Goloboff and Catalano 2016]; parsimony), MEGA (MEGA-CC v.10.1.8 [Kumar et al. 2012]; neighbor-joining), and MrBayes (v.3.2.7a [Ronquist et al. 2012]; Bayesian phylogeny). The inspection of MrBayes runs was performed with Tracer (v1.7.1; Rambaut et al. 2018).

## Supplementary Material

Supplementary data are available at *Molecular Biology and Evolution* online.

## Supplementary Material

msaa234_Supplementary_Data
