# Supplementary material for: Complex Evolution of Light-Dependent Protochlorophyllide Oxidoreductases in Aerobic Anoxygenic Phototrophs: Origin, Phylogeny, and Function
Source: Mol Biol Evol. 2020 Sep 15;38(3):819–37. doi: 10.1093/molbev/msaa234 (PMC7947762; doi:10.1093/molbev/msaa234)
Supplement: msaa234_Supplementary_Data [file msaa234_supplementary_data.zip › Schneidewind_et_al_2020_SUPPLEMENT-R3_FINAL_unmarked.pdf]

Supplementary Materials

Complex evolution of light-dependent protochlorophyllide oxidreductases in aerobic anoxygenic phototrophs: origin, phylogeny and function

Olga Chernomor[1#], Lena Peters[2#], Judith Schneidewind[2#], Anita Loeschcke[2,3], Esther Knieps-Grünhagen[2], Fabian Schmitz[2], Eric von Lieres[5], Roger Jan Kutta[6], Vera Svensson[2], Karl-Erich Jaeger[2,5], Thomas Drepper[2], Arndt von Haeseler[1,4], Ulrich Krauss[2,5]*

[#:] **contributed equally, names are in alphabetical order**

[*:] **corresponding author**

Affiliations

[1:] Center for Integrative Bioinformatics Vienna, Max Perutz Labs, University of Vienna, Medical University of Vienna, Dr. Bohr Gasse 9, A-1030 Vienna, Austria.

[2:] Institut für Molekulare Enzymtechnologie, Heinrich-Heine-Universität Düsseldorf, Forschungszentrum Jülich GmbH, D-52425 Jülich, Germany

[3:] Cluster of Excellence on Plant Sciences (CEPLAS)

[4:] Faculty of Computer Science, University of Vienna, Währinger Str. 29, A-1090 Vienna, Austria.

[5:] Institute of Bio- and Geosciences IBG-1: Biotechnology, Forschungszentrum Jülich GmbH, D-52425 Jülich, Germany

[6:] Institut für Physikalische und Theoretische Chemie, Universität Regensburg, Universitätsstr. 31, 93053 Regensburg, Germany

Key words: light-driven enzyme, chlorophyll biosynthesis, evolution, aerobic anoxygenic phototrophic bacteria, photosynthesis

Abbreviations: Pchlide, protochlorophyllide; Chlide, chlorophyllide; BChl *a*, bacteriochlorophyll *a*; POR, protochlorophyllide oxidoreductase; GC content, guanine-cytosine content; AAPB, aerobic anoxygenic phototrophic bacteria; LPOR, light-dependent protochlorophyllide oxidreductase; DPOR, light-independent protochlorophyllide oxidreductase; HGT, horizontal gene transfer; GI, genomic island.

Table of Contents

Entry	Content	Page(s)
**1. Supplementary Results and Discussion**		3-40
**1.1 Presence/absence of marker proteins for aerobic anoxygenic phototrophs (AAPBs)**		3-11
Table S1	Taxonomy, species distribution and AAPB marker gene analysis for non-oxygenic phototrophic host organism containing a putative LPOR	4
Table S2	HMM models used in this study to scan the genomes for the presence/absence of marker proteins for (aerobic) anoxygenic photosynthesis	5
Table S3	Bacterial strains and plasmids used in this study	5
Figure S1	Multiple sequence alignment of all putative LPORs identified outside of oxygenic phototrophic genera	8
**1.2 *In vitro* and *in vivo* LPOR activity tests**		12-16
Figure S2	Light-dependent *in vitro* conversion of Pchlide by purified enzymes	12
Figure S3	Analysis of the light-dependent *in vitro* conversion of Pchlide by cell free lysates	13
Figure S4	*Rhodobacter capsulatus*-based LPOR in vivo assay for all selected AAPB LPORs	14
Figure S5	*Rhodobacter capsulatus*-based LPOR in vivo assay for all selected AAPB LPORs	15
Table S4	Activity comparison of AAPB LPORs by *in vivo* and *in vitro* assays	16
**1.3 Comparative biochemical characterization of AAPB, plant and cyanobacterial LPORs**		17-28
1.3.1 pH activity optima and range		17
Figure S6	pH activity optima determination	18
Table S5	pH activity optima and 80% pH-optimum range determined for the AAPB LPORs, *Te*LPOR and *At*LPORC	19
1.3.2 Temperature-activity optima, optimum temperature range and temperature dependent unfolding		19
Figure S7	Temperature activity optima determination	20
Table S6	Temperature-activity optima and 80% optimum-temperature range determined for AAPB LPORs, *Te*LPOR and *At*LPORC	20
Figure S8	Temperature-dependent unfolding studies	21
Table S7	DSF-derived melting-temperatures determined for AAPB LPORs, *Te*LPOR and *At*LPORC.	21
1.3.3 Dissociation of the NADPH/Pchlide/LPOR ternary complex		22
Figure S9	K_d determination for the ternary NADPH/Pchlide/LPOR complex without DTT	23
Figure S10	K_d determination for the ternary NADPH/Pchlide/LPOR complex with DTT	24
Table S8	K_d of the NADPH/Pchlide/LPOR ternary complex determined for AAPB LPORs, *Te*LPOR and *At*LPORC	25
1.3.4 MV/DV-Pchlide substrate preference		25
Figure S11	Analytical HPLC and LC-MS analysis of *R. capsulatus* ZY5 produced Pchlide	26
Figure S12	Preparative-scale purification of MV- and DV-Pchlide from the *R. capsulatus* ZY5 produced Pchlide	27
Figure S13	MV/DV Pchlide substrate acceptance determined by measuring the activity of the respective enzyme with either MV (black bars) or DV-Pchlide (red bars)	28
Table S9	Specific activities for the conversion of MV- and DV-Pchlide by LPORs.	28
**1.4 Phylogenetic tree reconstruction, evolutionary hypothesis evaluation**		29-36
1.4.1 Sequence selection and phylogenetic tree reconstruction		29
Table S10	Overview over the sequences used for tree inference	29
Figure S14	Consensus LPOR tree and exemplary phylogenetic LPOR tree with mapped activity	30
1.4.2 Analysis of evolutionary timeline		31
Table S11	Divergence times for relevant bacterial lineages.	31-32
1.4.3 Genomic Island (GI) analysis		32
Table S12	Summary of the GI analysis	34
Figure S15	LPOR-containing contigs and scaffolds among selected LPOR containing AAPBs	35
Table S13	Detailed results of the genomic island (GI) analysis for 21 AAPB and 23 cyanobacterial species	35
Figure S16	Visualization of genomic islands (GIs) in the vicinity of the LPOR encoding gene	36
1.4.4. Additional analysis with alternative methods		37
Figure S17	Pruned subtrees display the position of AAPB-clade in LPOR-trees inferred using alternative methods	37
1.4.5. Additional tree topology tests		38
Figure S18	Position of AAPB-clade on alternative trees, which survived the AU-test.	38
**1.5 Small angle X-ray scattering (SAXS) analyses**		39
Table S14	Protein concentration of LPOR samples used for SAXS measurements	40
Table S15	CRYSOL-derived χ values	40
**1.6 Characteristics of selected LPOR-containing AAPBs**		41
Table S16	pH and temperature growth (optima) characteristics of selected LPOR-containing AAPBs	41
**2. Supplementary References**		42-44

1. Supplementary Results and Discussion

1.1 Presence/absence of marker proteins for aerobic anoxygenic phototrophs (AAPBs).

Presence/absence of marker proteins for aerobic anoxygenic photosynthesis are shown in Figure 1A in the main manuscript and supplementary Table S1, which are discussed in the following.

Ribulose-1,5-bisphosphat-carboxylase/-oxygenase (RuBisCO) and phosphoribulokinase (PRK)

RuBisCO, with its absence often considered as a marker for AAPBs, was absent in most organisms. Exceptions here were *Gemmatimonas phototrophica*, *Burkholderiales* bacterium, *Limnohabitans* sp. 2KL-51, *Acidiphilium rubrum* and *Acidiphilium angustum*, which contained a copy of RuBisCo. About 50% of the analysed organisms contained a copy of PRK, whose absence likewise is considered as marker for aerobic anoxygenic photosynthesis.

Bacteriochlorophyll biosynthesis

The LPOR-containing *Gemmatimonadetes* bacterium apparently lacks both the BchN and BchB subunits of the DPOR enzyme complex. Some LPOR-containing *Erythrobacter* species (*Erythrobacteraceae* bacterium HL-111, *Erythrobacter* sp. SCN 62-14) lack only the BchL subunit. All of the analysed organisms contained oxygen-dependent (AcsF) magnesium-protoporphyrin IX monomethyl ester oxidative cyclase, considered as a marker for aerobic and semi-aerobic chlorophototrophs. Moreover, the majority of the analysed AAPBs lacked the oxygen-independent version of the enzyme (BchE). Exceptions from this rule are here *Gemmatimonas phototrophica*, *Dinoroseobacter shibae*, all *Erythrobacter* species and *Porphyrobacter dokdenensis*, which contained both AcsF and BchE.

Photosynthetic reaction center proteins

The LPOR- and DPOR-containing organisms Proteobacteria bacterium ST bin14 and *Sphingomonas* sp. 12-62-6 appear to lack both type I and type II RCs. All other bacteria possess type II RCs (contain both *puf*L and *puf*M).

AAPB classification

19 out of 36 considered bacteria were confirmed to be AAPBs in the previous studies, which also includes exceptions possessing RuBisCO (see Figure 1A). Based on the presence-absence of genetic markers, another 12 species appear to be AAPBs. For some of the analysed organisms, it is unclear whether they are truly phototroph, i.e. lacking key components required for bacteriochlorophyll biosynthesis or the assembly of the photosynthetic apparatus. It is tempting to speculate here that those organisms represent an evolutionary intermediate state, still possessing some of the proteins needed for bacteriochlorophyll synthesis and phototrophy, while apparently having lost others.

Table S1: Taxonomy, species distribution and AAPB marker gene analysis for non-oxygenic phototrophic host organism containing a putative LPOR

Taxonomy		Species	LPOR	BchB	bchN	bchL	RuBisCO	PRK	BchE	AcsF	PS I (PsaA_PsaB)	PS II (Photo_RC)	PS II - PufL	PS II - PufM	PS II - PsbA	PS II - PsbD	Assembly AccNUM	LPOR AccNUM	LPOR ID[d]
Gemmatimonadetes		*Gemmatimonas phototrophica*	1	1	1	1	1	2	1	1	0	2	1	1	0	0	GCA_000695095.2	AMW05784	*Gp*LPOR
Gemmatimonadetes		*Gemmatimonadetes bacterium*	1	0	0	1	0	1	0	1	0	2	1	1	0	0	GCA_002737115.1	PHX65690	
Alphaproteobacteria		*Acidiphilium angustum*	1	1	1	1	1	1	0	1	0	2	1	1	0	0	GCA_000701585.1	WP_029314395	*An*LPOR
Alphaproteobacteria		*Acidiphilium rubrum*	1	1	1	1	1	1	0	1	0	2	1	1	0	0	GCA_900156265.1	SIR58578	
Alphaproteobacteria		*Erythrobacter litoralis*[a]	1	1	1	1	0	0	1	1	0	2	1	1	0	0	GCA_000714795.1	KEO90002	*El*LPOR
Alphaproteobacteria		*Erythrobacter litoralis*[a]	1	1	1	1	0	0	1	1	0	2	1	1	0	0	GCA_001719165.1	AOL22630	
Alphaproteobacteria		*Erythrobacter* sp. HL-111[b]	1	1	1	1	0	0	1	1	0	2	1	1	0	0	GCA_900105095.1	SDS46897	
Alphaproteobacteria		*Erythrobacteraceae* bacterium HL-111[b]	1	1	1	0	0	0	1	1	0	2	1	1	0	0	GCA_001314765.1	KPP91129	*Eb*LPOR
Alphaproteobacteria		*Erythrobacter* sp. SCN 62-14	1	1	1	0	0	0	1	1	0	2	1	1	0	0	GCA_001724215.1	ODS93018	
Alphaproteobacteria		*Porphyrobacter dokdonensis* DSM 17193[c]	1	1	1	1	0	0	1	1	0	2	1	1	0	0	GCA_002155305.1	WP_068863292	
Alphaproteobacteria		*Porphyrobacter dokdonensis* DSW-74[c]	1	1	1	1	0	0	1	1	0	2	1	1	0	0	GCA_001677335.1	OBV11559	*Pd*LPOR
Alphaproteobacteria		*Porphyrobacter tepidarius*	1	1	1	1	0	0	0	1	0	2	1	1	0	0	GCA_002155695.1	WP_086617938	
Alphaproteobacteria		Alphaproteobacteria bacterium	1	1	1	1	0	1	0	1	0	2	1	1	0	0	GCA_003241875.1	PZN94637	
Alphaproteobacteria		Alphaproteobacteria bacterium PA4	1	1	1	1	0	1	0	2	0	2	1	1	0	0	GCA_002256005.1	OYU15985	
Alphaproteobacteria		*Sphingomonadales* bacterium 28-64-96	1	1	1	1	0	1	0	1	0	2	1	1	0	0	GCA_002281615.1	OYZ16473	
Alphaproteobacteria		*Sphingomonadales* bacterium 32-65-25	1	1	1	1	0	1	0	1	0	2	1	1	0	0	GCA_002280855.1	OYX79084	
Alphaproteobacteria		*Dinoroseobacter shibae*	1	1	1	1	0	0	1	1	0	2	1	1	0	0	GCA_000018145.1	ABV95870	*Ds*LPOR
Alphaproteobacteria		*Loktanella fryxellensis*	1	1	1	1	0	1	0	1	0	2	1	1	0	0	GCA_900110065.1	SEN87055	*Lf*LPOR
Alphaproteobacteria		*Yoonia vestfoldensis*	1	1	1	1	0	1	0	1	0	2	1	1	0	0	GCA_000382265.1	WP_026352701	*Yv*LPOR
Alphaproteobacteria		*Roseisalinus antarcticus*	1	1	1	1	0	2	0	1	0	2	1	1	0	0	GCA_900172355.1	SLN77620	
Alphaproteobacteria		*Sulfitobacter guttiformis*	1	1	1	1	0	0	0	1	0	2	1	1	0	0	GCA_000622425.1	KIN73509	*Sg*LPOR
Alphaproteobacteria		*Sandarakinorhabdus limnophila*	1	1	1	1	0	1	0	1	0	2	1	1	0	0	GCA_000420765.1	WP_022680993	*Sl*LPOR
Alphaproteobacteria		*Sandarakinorhabdus* sp. AAP62	1	1	1	1	0	1	0	1	0	2	1	1	0	0	GCA_000331225.1	WP_020480449	*Sa*LPOR
Alphaproteobacteria		*Sandarakinorhabdus* sp. TH057	1	1	1	1	0	1	0	1	0	2	1	1	0	0	GCA_002251755.1	OYQ31188	
Alphaproteobacteria		*Sphingomonas* sp. 12-62-6	1	1	1	1	0	0	0	1	0	2	1	1	0	0	GCA_002279655.1	OYW22568	
Alphaproteobacteria		*Sphingomonas* sp. 28-62-11	1	1	1	1	0	0	0	2	0	2	1	1	0	0	GCA_002278865.1	OYY63160	
Alphaproteobacteria		*Sphingomonas* sp. 28-62-20	1	1	1	1	0	0	0	1	0	2	1	1	0	0	GCA_002281505.1	OYY75650	*Sp*LPOR
Alphaproteobacteria		*Sphingomonas* sp. 32-62-10	1	1	1	1	0	0	0	2	0	2	1	1	0	0	GCA_002280555.1	OYX39646	
Alphaproteobacteria		*Sphingomonas* sp. IBVSS1	1	1	1	1	0	1	0	1	0	2	1	1	0	0	GCA_002127205.1	OSZ70988	
Betaproteobacteria		*Polynucleobacter paneuropaeus*	1	1	1	1	0	0	0	1	0	2	1	1	0	0	GCA_003261235.1	AWW49216	
Betaproteobacteria		*Polynucleobacter campilacus*	1	1	1	1	0	0	0	1	0	2	1	1	0	0	GCA_002206625.1	OWS70994	
Betaproteobacteria		*Limnohabitans* sp. 15K	1	1	1	1	0	0	0	1	0	2	1	1	0	0	GCA_002778285.1	PIT82811	
Betaproteobacteria		*Limnohabitans* sp. 2KL-51	1	1	1	1	2	1	0	1	0	2	1	1	0	0	GCA_003063645.1	PUE50165	
Betaproteobacteria		*Burkholderiales* bacterium	1	1	1	1	1	1	0	1	0	2	1	1	0	0	GCA_003241965.1	PZO15167	
uncl. Proteobacteria		Proteobacteria bacterium ST_bin13	1	1	1	1	0	0	0	1	0	2	1	1	0	0	GCA_002083645.1	OQW77747	
uncl. Proteobacteria		Proteobacteria bacterium ST_bin14	1	1	1	1	0	1	0	1	0	0	0	0	0	0	GCA_002083655.1	OQW73116	
Cyanobacteria		*Thermosynechococcus elongatus* BP-1	1	1	1	1	1	1	0	2	2	5	0	0	3	2	GCA_000011345.1	BAC08127.1	*Te*LPOR[e]
Cyanobacteria		*Synechocystis* sp. PCC 6803	1	1	1	1	1	1	0	2	2	5	0	0	3	2	GCA_001318385.1	ALJ68773.1	*Ss*LPOR[e]
Spermatophyta		*Hordeum vulgare*[f]	-	-	-	-	-	-	-	-	-	-	-	-	-	-	-	P13653	*Hv*LPORA[e]
Tracheophyta		*Arabidopsis thaliana*	6	0	0	0	2	38	0	2	2	2	0	0	1	1	GCF_000001735.4_	AEE27591.1	*At*LPORC[e]
Actinobacteria		*Saccharopolyspora erythreae*	0	0	0	0	0	2	1	0	0	0	0	0	0	0	GCA_000448385.1	EQD83656.1	*Se*SDR[g]

[a,b,c:] represent two identical LPORs each. Therefore only one of each was included in the phylogenetic analysis. [d:] LPOR ID used to identify experimentally characterized enzymes; presence-absence analysis was performed for: LPOR: light-dependent protochlorophyllide oxidoreductase; the BchB, BchN, BchL subunits of the dark-operative protochlorophyllide oxidoreductase; RBC: large (catalytics) subunit of ribulose-1,5-bisphosphat-carboxylase/-oxygenase; PRK: phosphoribulokinase; oxygen-independent (BchE) and oxygen-dependent (AcsF) magnesium-protoporphyrin IX monomethyl ester oxidative cyclase; Photosynthetic reaction center type assessed as: PS I (PsaA/PsaB): as verified by PsaA/PsaB Pfam HMM; PS II (Photo_RC): verified by photosynthetic reaction center protein HMM, which includes the PufL, PufM, PsbA/D1 and PsbD/D2 subfamilies; To address which of the four subfamilies is identified by the Photo_RC HMM, TIGRFAM HMM models for the individual proteins were used.[e:] reference LPORs used in this study. [f:] For *Hordeum vulgare* no assembly with proteome information is available. [g]: putative short-chain dehydrogenase of *Saccharopolyspora erythraea* used as negative control.

Table S2: Hidden Markov Models (HMM) used in this study to scan the genomes for the presence/absence of genes encoding the listed marker proteins for (aerobic) anoxygenic photosynthesis. [a:] large (catalytic) subunit of RuBisCo common to type I-IV RuBisCO (Tabita, et al. 2008)

**Protein**	**HMM**	
	**TIGRFAMs**	**Pfam**
LPOR	TIGR01289	
DPOR_BchB	TIGR01278	
DPOR_BchN	TIGR01279	
DPOR_BchL	TIGR01281	
RuBisCO		RuBisCO_large[a]
PRK		PRK
BchE	TIGR02026	
AcsF	TIGR02029	
PS I		PsaA_PsaB
PS II		Photo_RC
PufL	TIGR01157	
PufM	TIGR01115	
PsbA	TIGR01151	
PsbD	TIGR01152	

Table S3: Bacterial strains and plasmids used in this study.

**Bacterial strain**	**Relevant genotype**	**Reference**
Escherichia coli BL21(DE3)	F^- *ompT gal dcm lon hsd*$S_B(r_B^-, m_B^-)$ λ(DE3[*lac*I *lac*UV5-T7 gene 1 *ind*1 *sam*7 *nin*5])	Invitrogen
Escherichia coli DH5α	F^- Φ80*lac*ZΔM15 Δ(*lac*ZYA-*arg*F) U169 *rec*A1 *end*A1 *hsd*R17 (r_K^-, m_K^+) gal^- *pho*A *sup*E44 λ^- thi^-1 *gyr*A96 *rel*A1	Invitrogen
Escherichia coli S17-1	F^- *rec*A *thi pro hsd*R^-M^+ RP4-2-Tc::Mu-Km::Tn7	(Simon, et al. 1983)
Rhodobacter capsulatus B10S	spontaneous Sm^R mutant of B10	(Klipp, et al. 1988)
Rhodobacter capsulatus ΔbchB	B10S derivative, *ΔbchB,* Sp^R	(Kaschner, et al. 2014)
Rhodobacter capsulatus ZY5	SB1003 derivative, F108::Km^R *rif-10*	(Yang and Bauer 1990)
Dinoroseobacter shibae	DFL12T, wildtype isolate	(Wagner-Döbler, et al. 2010)
**Plasmid name**	**Relevant features**	**Reference**
a) Heterologous over-expression in *E.coli*		
pET28a	Km^R, P_{T7}, N- and C-terminal His-tag, *lacI*	Novagen
pET28a_*An*LPOR	pET28a derivative, 983 bp *AnLPOR* fragment *Nde*I/*Sal*I, P_{T7}>*AnLPOR* N-terminal His-tag	this study
pET28a_*At*LPORC	pET28a derivative, 1205 bp *AtLPORC* fragment *Nco*I/*Xho*I, P_{T7}>*AtLPORC* C-terminal His-tag	this study

pET28a_*Ds*LPOR	pET28a derivative, 989 bp *DsLPOR* PCR product *Nde*I/*Sal*I, P_{T7}>*DsLPOR* N-terminal His-tag	(Kaschner, et al. 2014)
pET28a_*Eb*LPOR	pET28a derivative, 986 bp *EbLPOR* fragment *Nde*I/*Xho*I, P_{T7}>*EbLPOR* N-terminal His-tag	this study
pET28a_*El*LPOR	pET28a derivative, 986 bp *ElLPOR* fragment *Nde*I/*Sal*I, P_{T7}>*ElLPOR* N-terminal His-tag	this study
pET28a_*Gp*LPOR	pET28a derivative, 971 bp *GpLPOR* fragment *Nde*I/*Sal*I, P_{T7}>*GpLPOR* N-terminal His-tag	this study
pET28a_*Hv*LPORA	pET28a derivative, 1169 bp *HvLPORA* fragment *Nde*I/*Sal*I, P_{T7}>*HvLPORA* N-terminal His-tag	this study
pET28a_*Lf*LPOR	pET28a derivative, 983 bp *LfLPOR* fragment *Nde*I/*Sal*I, P_{T7}>*LfLPOR* N-terminal His-tag	this study
pET28a_*Yv*LPOR	pET28a derivative, 965 bp *YvLPOR* fragment *Nde*I/*Sal*I, P_{T7}>*YvLPOR* N-terminal His-tag	this study
pET28a_*Pd*LPOR	pET28a derivative, 1001 bp *PdLPOR* fragment *Nde*I/*Sal*I, P_{T7}>*PdLPOR* N-terminal His-tag	this study
pET28a_*Sa*LPOR	pET28a derivative, 974 bp *SaLPOR* fragment *Nde*I/*Xho*I, P_{T7}>*SaLPOR* N-terminal His-tag	this study
pET28a_*Se*SDR	pET28a derivative, 905 bp *SeSDR* fragment *Nde*I/*Sal*I, P_{T7}>*SehGCLPOR* N-terminal His-tag	this study
pET28a_*Sg*LPOR	pET28a derivative, 962 bp *SgLPOR* fragment *Nde*I/*Sal*I, P_{T7}>*SgLPOR* N-terminal His-tag	this study
pET28a_*Sl*LPOR	pET28a derivative, 986 bp *SlLPOR* fragment *Nde*I/*Sal*I, P_{T7}>*SlLPOR* N-terminal His-tag	this study
pET28a_*Sp*LPOR	pET28a derivative, 1001 bp *SpLPOR* fragment *Nde*I/*Sal*I, P_{T7}>*SpLPOR* N-terminal His-tag	this study
pET28a_*Ss*LPOR	pET28a derivative, 959 bp *SsLPOR* fragment *Nde*I/*Sal*I, P_{T7}>Ss*LPOR* N-terminal His-tag	this study
pET28a_*Te*LPOR	pET28a derivative, 971 bp *TeLPOR* fragment *Nde*I/*Sal*I, P_{T7}>*TeLPOR* N-terminal His-tag	this study
b) Complementation of *R. capsulatus ΔbchB* deletion strain		
pRhokHi-2	Km^R, constitutive $P_{aphII,}$ promoter for heterologous gene expression	(Katzke, et al. 2010)
pRhokHi-2_*An*LPOR	pRhokHi-2 derivative, 1103 bp *Xba*I/*Xho*I *His$_6$-AnLPOR* fragment from pET derivative; P_{aphII}>*AnLPOR* N-terminal His-tag	this study
pRhokHi-2_*At*LPORC	pRhokHi-2 derivative, 1328 bp *Xba*I/*Xho*I *His$_6$-AtLPOR* fragment from pET derivative; P_{aphII}>*AtLPOR* N-terminal His-tag	this study
pRhokHi-2_*Ds*LPOR	pRhokHi-2 derivative, P_{aphII}>*DsLPOR* C-terminal His-Tag	(Kaschner, et al. 2014)

pRhokHi-2_*Eb*LPOR	pRhokHi-2 derivative, 1085 bp *Xba*I/*Xho*I His_6-*EbLPOR* fragment from pET derivative; P_{aphII}>*EbLPOR* N-terminal His-tag	this study
pRhokHi-2_*El*LPOR	pRhokHi-2 derivative, 1106 bp *Xba*I/*Xho*I His_6-El*LPOR* fragment from pET derivative; P_{aphII}>*ElLPOR* N-terminal His-tag	this study
pRhokHi-2_*Gp*LPOR	pRhokHi-2 derivative, 1076 bp *Xba*I/*Hind*III His_6-*GpLPOR* fragment from pET derivative; P_{aphII}>*GpLPOR* N-terminal His-tag	this study
pRhokHi-2_*Hv*LPORA	pRhokHi-2 derivative, 1289 bp *Xba*I/*Xho*I His_6-*HvLPOR* fragment from pET derivative; P_{aphII}>*HvLPOR* N-terminal His-tag	this study
pRhokHi-2_*Lf*LPOR	pRhokHi-2 derivative, 1103 bp *Xba*I/*Xho*I His_6-*LfLPOR* fragment from pET derivative; P_{aphII}>*LfLPOR* N-terminal His-tag	this study
pRhokHi-2_*Yv*LPOR	pRhokHi-2 derivative, 1085 bp *Xba*I/*Xho*I His_6-*YvLPOR* fragment from pET derivative; P_{aphII}>*YvLPOR* N-terminal His-tag	this study
pRhokHi-2_*Pd*LPOR	pRhokHi-2 derivative, 1100 bp *Xba*I/*Sal*I His_6-*PdLPOR* fragment from pET derivative; P_{aphII}>*PdLPOR* N-terminal His-tag	this study
pRhokHi-2_*Sa*LPOR	pRhokHi-2 derivative, 1073 bp *Xba*I/*Xho*I His_6-*SaLPOR* fragment from pET derivative; P_{aphII}>*SaLPOR* N-terminal His-tag	this study
pRhokHi-2_*Se*SDR	pRhokHi-2 derivative, 1025 bp *Xba*I/*Xho*I His_6-*SeSDR* fragment from pET derivative; P_{aphII}>*SeSDR* N-terminal His-tag	this study
pRhokHi-2_*Sg*LPOR	pRhokHi-2 derivative, 1082 bp *Xba*I/*Xho*I His_6-*SgLPOR* fragment from pET derivative; P_{aphII}>*SgLPOR* N-terminal His-tag	this study
pRhokHi-2_*Sl*LPOR	pRhokHi-2 derivative, 1070 bp *Xba*I/*Xho*I His_6-*SlLPOR* fragment from pET derivative ; P_{aphII}>*SlLPOR* N-terminal His-tag	this study
pRhokHi-2_*Sp*LPOR	pRhokHi-2 derivative, 1121 bp *Xba*I/*Xho*I His_6-*SpLPOR* fragment from pET derivative; P_{aphII}>*SpLPOR* N-terminal His-tag	this study
pRhokHi-2_*Ss*LPOR	pRhokHi-2 derivative, 1079 bp *Xba*I/*Xho*I His_6-*SsLPOR* fragment from pET derivative; P_{aphII}>*SsLPOR* N-terminal His-tag	this study
pRhokHi-2_*Te*LPOR	pRhokHi-2 derivative, 1091 bp *Xba*I/*Xho*I His_6-*TeLPOR* fragment from pET derivative; P_{aphII}>*TeLPOR* N-terminal His-tag	this study

```
AnLPOR      : -----------MEATTPPHVIVTGASSGVGLHATKALVDRGWHVVMACRDLAKAARAAANLDINPASITPLEIDLGSQSSVRRFAAD----FAALVKP :  83
DsLPOR      : -----------MTLDPMPRAIVTGASSGVGLHATKSLIDRGWHVTMACRDLAKAEAAARSLDLDPGRYALAHLDLGSLASVRAFHAN----IARDHDS :  83
EbLPOR      : -----------MATGHTPICIITGASSGIGLWATKALVTRGWHVVMACRDTAKAEAAAREIGIAPEGRTIMRLDLGSLAGVRAFAEE----FRAFDRP :  83
ElLPOR      : -----------MIAGQTPVCIITGASSGIGLWATRALMARGWHVVMACRDTAKAEAAAREIGLAPEGRTIMQLDLGTLSGVRGFVSE----FRMLGRP :  83
GpLPOR      : -------------MSTAPTCIITGASSGVGLYGAKSLAARGWHVVMACRDLAKATAAADALGIPKASRTLMPIDLGSQASVRAFVEA----FRATGRA :  81
LfLPOR      : -----------MSVQHHPLCIVTGASSGVGLHSTKALIDKGWHVIMACRDLEKAAAAAQSLNLQRDMFDVMHLDLGSLDSVRAFHAA----YRAQNRA :  83
LvLPOR      : -----------MPQDNQPLAIVTGASSGVGLHTTNALIGRGWHVIMACRDLAKAETAARSLDMASGSYELAHIDLGSLDSVRAFYAG----FRARGVP :  83
PdLPOR      : -----------MTDLPKKTALVTGASSGVGLWSAKALADRGWHVIMACRDLAKAESAAREAGIAPANRTLLHVDLADFASVRALAEAARETVRAGGGA :  87
SaLPOR      : -------------MSIQPVVIITGASSGVGLWAARSLATRGWLVIMACRDLAKAEAAATEIGIPAESRRIMPIDLGDQASVRKFADE----FHATGLP :  81
SgLPOR      : -----------MTRSTKQLAIVTGASSGVGLYSTQALIAKGWQVIMACRNMEKAFQAAHDLGLPTDAYQIMHLDLGSLSSVRAFHAA----FRAQNRA :  83
SlLPOR      : ---------MSVQAAVQPTAIITGASSGVGLWAAKSLADRGWLVIMACRDLAKAEAAAAEVGIPADSRRILHIDLGDQASVRRFADE----FHGLGLP :  85
SpLPOR      : -----------MTTAKLPLAIITGASSGVGLYATAALIGRGWHVIMACRDLDKAAAAATGLGLRTDRYEIAHLDLASQASVRAFAAA----FRASDRP :  83
OWS70994    : ----------MTSTTNQPLAIITGSSSGVGLYAAKALLARKWRLILAVRDPQKMELAAKAHQFDSSQYEIWQLDLGNLDSVRAFVKR----FNDSGQK :  84
AWW49216    : ----------MTSTTTQPLAIITGSSSGVGLYTAKALLARNWRLILAVRDPQKMELVAKAHQFDSSQYEIWQLDLGNLDSVHAFVKR----FNDGGQK :  84
OYQ31188    : --------MSDQPAIDQPVVIITGASSGVGLWAAKSLADRGWMVIMACRDLAKAEAAANEIGVNAANRRILHIDLGDQASVRKFADD----FHALGLP :  86
OSZ70988    : --------MSDQPAIDQPVVIITGASSGVGLWAAKSLADRGWMVIMACRDLAKAEAAANEVGVNAANRRILHIDLGDQASVRKFADD----FHALGLP :  86
OYX79084    : ---------MSVQAAVQPTAIITGASSGVGLWAAKSLADRGWLVIMACRDLAKAEAAAAEVGITGDSRRILHIDLGDQASVRRFADE----FHGLGLP :  85
OYZ16473    : ---------MSVQAAVQPTAIITGASSGVGLWAAKSLADRGWLVIMACRDLAKAEAAAAEVGIPADSRRILHIDLGDQASVRRFADE----FHGLGLP :  85
OQW73116    : -----------MNSSPPSLAIITGASSGVGLYTTAALIERGWHVIMACRDIEKAAVAADEIGLRADRFEIAHLDLASLASVRAFAAA----FRESGRP :  83
OQW77747    : -----------MTTAKLPLAIITGASSGVGLYATAALIGRGWHVIMACRDLEKAAAAATGLGLRTDRYEIAHLDLASQASVRAFAAA----FRASDRP :  83
OYY63160    : -----------MTTANLPLAIITGASSGVGLYATASLIERGWHVIMACRDLNKAAAAATGLGLPKDRYDLVHLDLASLESVRGFAAA----FRASGRP :  83
OYW22568    : -----------MTKANLPLAIITGASSGVGLYATASLIERGWHVIMACRDLNKAAAAATGLGLPKDRYDLVHLDLASLESVRGFAAA----FRASGRP :  83
OYX39646    : -----------MTKANLPLAIITGASSGVGLYATASLIERGWHVIMACRDLNKAAAAATGLGLPKDRYDLVHLDLASLESVRGFAAA----FRASGRP :  83
SLN77620    : -----------MPQDNQPLAIVTGASSGVGLHTTNALIGRGWHVIMACRDLAKAETAARSLDMASGSYELAHIDLASLDSVRAFYAG----FRARGVP :  83
OYU15985    : -----------MAGNKSATVIITGASSGVGLWAAKALADRGWHVVMACRDLAKAEAAAVEVGIPSDRRTLLHIDLGSLASVRQFVSD----FNALKRP :  83
PZN94637    : -----------MDARTVPVVIITGASSGVGLWSAKALADRGWHVVMACRDVAKAEAAAVATGIPATARTIMPIDLGDLASVRAFVAA----FTATGLR :  83
ODS93018    : -----------MNTSAQPTALITGASSGVGLWAAYALTQRGWHVIMACRNLAKAEAAAREIGIDPAKRTVMHLDLADFASVRALAENTREWLNAQGRT :  87
PIT82811    : -----------MNTLRKPHVIVTGASSGVGLYATVALIQQGWHVVMACRDLQKAARVAAEMQLEASKFSLMKLDLGSLQSVREFVKD----FESKGLP :  83
PUE50165    : ---MRLAPSTPTNQPLSPIVIVTGASSGVGLYATASLIQLGWHVVMACRDLAKAQRVAAEMQLPAGHFSLMHLDLGSLQSVRDFASA----YLALDWP :  91
SIR58578    : MQGENLAAVETMEATTPPHVIVTGASSGVGLHATKALVDRGWHVVMACRDLAKAARAAANLDINPASITPLEIDLGSQSSVRRFAAD----FAALVKP :  94
PHX65690    : -------------MPLVSTVIVTGASSGVGLYATKSLITRGWHVVMACRDLAKASRAAESLELPAGQYTLSHLDLGVQASVRAFVAA----FLASSRE :  81
PZO15167    : -------MHQSPTPPVVPTVIVTGASSGVGLHATKSLTERGWFVVMACRDTAKAARVASELAIPASHHAVLEIDLGAQASVRRFVDS----FRALGRP :  87
WP_086617938 : -----------MRTTAQPTALITGASSGVGLWTAQALVARGWQVIMACRDLAKAQDAASAVGIAPGNCTLMHLDLADFDSVRALAIAAREHLRARGGA :  87
```

```
AnLPOR      : LDALVCNAAVYLPLLKTPARSPEGFEISVATNHFGHFLLSHLLLPHLQSPGR--------RAKRLVTLGTVTANSEEFGGKIPIPAPANLGALA : 169
DsLPOR      : LDALVCNAAVYKPRLTQPGRSPDGFEISVATNYFGHFLLANLMLPLLEGAP----------SPRLITLGTVTANSEEFGGKVPIPAPADLGDFA : 167
EbLPOR      : LDALVCNAATYMPRLAEPLRSPEGFELSVATNYFGHFLLANLLLDELVRAR----------DARLVTLGTVTANSEEFGGKIPIPAPADLGDFA : 167
ElLPOR      : LDALVCNAATYMPRLTAPLRSPEGYELSVAINYFGHFLLANLLLEDLGRAR----------DPRLVTLGTVTANSEEFGGRIPIPAPADLGDFE : 167
GpLPOR      : LDALVCNAAVYLPRLTEPMRSPEGYEISVATNHFGHFLLANLLLPDLQRST--------HASRRLIILGTVTANYEEFGGKIPIPAPADLGNLE : 167
LfLPOR      : LDALVCNAATYLPQLKVPLRSPDGYEISVATNYLGHFLLANLMLEDLQKSP----------APRLVTLGTVTANSEEFGGKVPIPAPADLGDFQ : 167
LvLPOR      : LDALVCNAAVYLPLLKTPARSPEGFEISVATNYFGHFLLANLMLADLQKSA----------APRLITLGTVTANSEEFGGKVPIPAPADLGELQ : 167
PdLPOR      : LDALVLNAAVYLPRAEAPQRNADGYEISVATNYLGHFLLANLLLPDLEAAP----------APRLVTLGTVTANSEEFGGKIPIPAPADLGDLE : 171
SaLPOR      : LDALLLTAAVYLPRLEAPMRSAQGYEISVATNYFGHFLLAHLLIDDLKQAKGRL-----ASPPRLITLGTVTANSEEFGGKIPIPAPADLGDLA : 170
SgLPOR      : LDTLISNAATYLPQLKQPARSPEGFEISVATNYLGHFLLANLMLEDLQKSH----------LPRLVTLGTVTANSEEFGGKIPIPAPADLGEFQ : 167
SlLPOR      : LDALLLNAAVYLPRLEAPMRSPEGYEISVATNYFGHFLLAHLLLDDLKKAKGRL-----PSPPRLITLGTVTANSEEFGGKVPIPAPAELGDLA : 174
SpLPOR      : LDALVCNAATYLPLLKEPARSPEGYELNVATNYLGHFLLANLMLDDLQRAP----------APRLVTLGTVTANSEEFGGRIPIPAPADLGDFE : 167
OWS70994    : LNALLCNAATYLPLLKEPARSPQGFEISVATNYFGHYVLSRMLLENLIQTAK------AHEHARLITLGTVTANSEEFGGKVPIPAPADIGALE : 172
AWW49216    : LNALLCNAATYLPLLKEPARSPQGFEISVATNYFGHYVLSRMLLENLKQTAA------KGEHARLITLGTVTANSEEFGGKVPIPAPADIGALE : 172
OYQ31188    : LDALLLNAAVYLPRLEAPMRSPEGYEISVATNYFGHFLLAHLLIGDLKASNGRPKGKLGASPSRLITLGTVTANSEEFGGKIPIPAPADLGDLS : 180
OSZ70988    : LDALLLNAAVYLPRLEAPMRSPEGYEISVATNYFGHFLLAHLLIGDLKASNGRR-----GQPSRLITLGTVTANSEEFGGKIPIPAPADLGDLS : 175
OYX79084    : LDALLLNAAVYLPRLEAPMRSPEGYEISVATNYFGHFLLAHLLLDDLKKAKGRL-----PSPPRLITLGTVTANSEEFGGKVPIPAPAELGDLA : 174
OYZ16473    : LDALLLNAAVYLPRLEAPMRSPEGYEISVATNYFGHFLLAHLLLDDLKKAKGRL-----PSPPRLITLGTVTANSEEFGGKVPIPAPAELGDLA : 174
OQW73116    : LDALVCNAATYLPLLKQPERSPEGYELSVATNYLGHFLLANLMLADLQRAP----------APRLVTLGTVTANSEEFGGRIPIPAPADLGDLE : 167
OQW77747    : LDALVCNAATYLPLLKEPARSPEGYELNVATNYLGHFLLANLMLDDLQRAP----------APRLVTLGTVTANSEEFGGRIPIPAPADLGDFE : 167
OYY63160    : LDALVCNAATYLPLLKEPARSPEGYELSVATNYLGHFLLANLMLDDLKRAP----------APRLVTLGTVTANSEEFGGRIPIPAPADLGNFE : 167
OYW22568    : LDALVCNAATYLPLLKEPARSPEGYELSVATNYLGHFLLANLMLDDLKRAP----------APRLVTLGTVTANSEEFGGRIPIPAPADLGNFE : 167
OYX39646    : LDALVCNAATYLPLLKEPARSPEGYELSVATNYLGHFLLANLMLDDLKRAP----------APRLVTLGTVTANSEEFGGRIPIPAPADLGNFE : 167
SLN77620    : LDALVCNAAVYLPLLKTPARSPEGFEISVATNYFGHFLLANLMLADLQKSA----------APRLITLGTVTANSEEFGGKVPIPAPADLGEFQ : 167
OYU15985    : LDALVCNAAVYLPRLTAPMRSPEGYEISVATNYFGHFLLANLLLPNLERAQ----------APRLITLGTVTANSEEFGGKVPIPAPADIGELQ : 167
PZN94637    : LDALVCNAAVYLPRLAAPLRSPDGYEISVATNYFGHFLLANLLLPRLGKAP----------APRLVTLGTVTANSEEFGGKVPIPAPADLGDLQ : 167
ODS93018    : LDALVLNAAVYLLRAEEPQRNGQGYELSVATNYLGHFLLANLLLGDLAKAP----------APRLITLGTVTANSEEFGGKIPIPAPANLGELA : 171
PIT82811    : LHALLNNAASYQPRLKTPARSPEGYELSVATNHLGHFLLSRLLMPLLIRTQDSERVSHPDFHSRLVTLGTVTANSEEFGGKVPIPAPANLGDLA : 177
PUE50165    : LHALLNNAASYQPRLKTPARSPEGYELSVATNHLGHFLLSRLLMDLLLSTQEQERAQQPAFRARLITLGTVTANSEEFGGKVPIPAPADLGDLA : 185
SIR58578    : LDALVCNAAVYLPLLKTPARSPEGFEISVATNHFGHFLLSHLLLPHLQSPGR--------RAKRLVTLGTVTANSEEFGGKIPIPAPANLGALA : 180
PHX65690    : LNALVNNAAVYKPRLTEPARSIEGYEMSVATNHLGHFLLSRLLMDTLQQT----------KSARMITLGTVTANAEEFGGKVPIPAPADLGELE : 165
PZO15167    : LNVLLNNAAVYLPRLKEPQRSPEGFEISVATNHLGHFLLSRLLMNDLQQAGGA----QQGQPARLITLGTVTANSEEFGGKVPIPAPADLGNLQ : 177
WP_086617938 : LDALVLNAAVYLPRVAAPLRNPDGFELSVATNYLGHFLLANLLLPDLQAAP----------APRLVALGTVTANSEEFGGRVPIPAPADLGDFE : 171
```

```
AnLPOR       : GLEAGFRAPVAMIDGKKFKPGKAYKDSKLCNMIISREFHRRYHQNTGIIFNTLYPGCVADTALFRDAPKLFQTIFPWFQKTITKGYVSQALAGTRVA : 266
DsLPOR       : GLKAGFRAPVAMIDGKPFKAGKAYKDSKLCTMMMSRELHTRHHARTGIVFATLYPGCVADTPLFRDTPKAFQTIFPWFQKNVTKGYVSQALSGERVA : 264
EbLPOR       : GLKAGFTGPHAMIDGKPFKPGKAYKDSKLACMMMSREFHARFHESTGIVFTTLYPGCVADTPLFRHAPRAFQRIFPWFQKNITGGYVSQPLAGERVA : 264
ElLPOR       : GLKAGFKDPVAMIDGNPFKPGKAYKDSKLACMMMSRELHARFHDRTGIVFTTLYPGCVADTPLFRHAPRAFQRIFPWFQKNVTGGYVSQPLAGERVA : 264
GpLPOR       : GLEAGFLAPIAMLNGKAFKPGKAYKDSKLCNMITGRELHRRYHGQTGIVFNTLYPGCVADTPLFRHTPPAFQRIFPWFQKNITKGYVTQELAGDRLA : 264
LfLPOR       : GMKAGFKDPIAMIDGKPFKPGKAYKDSKLCCMMMSRELHARFHDRTGIVFSTLYPGCVADTPLFRNAPRLFQNIFPWFQKKITKGYVSQPLSGERVA : 264
LvLPOR       : GLKAGFKAPVAMIDGKPFKAGKAYKDSKLGTMVMSRELHTRFHDETGIVFSTLYPGCVADTPLFRNAPPLFQKVFPWFQKNITKGYVSQSLSGERVA : 264
PdLPOR       : GLEAGFRDPVAMIDGKPFKPGKAYKDSKLALMIMSRELHARFHDKTGIIFATLYPGCVADTPLFRHAPKAFQTIFPWFQKNITKGYVSQPLSGERVA : 268
SaLPOR       : GLAAGFQAPIAMIDGKPFKPGKAYKDSKLACMIMSREFQKRWG-STGIVFNTLYPGCVAETLLFRHTPPAFQKIFPWFQKNITKGYVSQPLAGERTA : 266
SgLPOR       : GLKDGFQAPVSMIDGKPFKPGKAYKDSKLCCMMMSRELHARYHEDTGIVFSTLYPGCVAETPLFRNAPALFQKVFPWFQKNITKGYVSQPLSGARVA : 264
SlLPOR       : GLAAGFKAPIAMIDGKPFKPGKAYKDSKLACMIMNREFHKRWG-DTGIVFNTLYPGCVADTALFRDTPQAFRTIFPWFQKNITKGYVSQPLAGDRTA : 270
SpLPOR       : GLAAGFKAPIAMIDGKPFKGGKAYKDSKLCTMMMSRELHARYHAQTGIIFSTLYPGCVADTPLFRNAPKLFQKIFPWFQKNITKGYVSQPLSGDRVA : 264
OWS70994     : GLMAGFKAPIAMINGKPFKPGKAYKDSKLCNMVMNRELQKRYHASTGVIFNTLYPGCVAETALFRDTPPLFQKIFPWFQKNITKGYVSQELAGDRVA : 269
AWW49216     : GLMAGFKAPVAMINGKPFKPGKAYKDSKLCNMVMNRELQKRYHASTGIIFNTLYPGCVAETALFRDTPPLFQKIFPWFQKNITKGFVSQELAGERVA : 269
OYQ31188     : GLASGFKAPIAMIDGKDFKPGKAYKDSKLACMIMSREFHKRWA-DTGVVFNTLYPGCVADTPLFRHTPPAFQRIFPWFQKNITKGYVSQPLAGERTA : 276
OSZ70988     : GLASGFKAPIAMIDGKDFKPGKAYKDSKLACMIMSREFHKRWA-NTGVVFNTLYPGCVADTPLFRHTPPAFQRIFPWFQKNITKGYVSQPLAGERTA : 271
OYX79084     : GLEAGFKAPIAMIDGKPFKPGKAYKDSKLACMIMNREFHKRWG-DTGIVFNTLYPGCVADTALFRDTPQAFRTIFPWFQKNVTKGYVSQPLAGDRTA : 270
OYZ16473     : GLAAGFKAPIAMIDGKPFKPGKAYKDSKLACMIMNREFHKRWG-DTGIVFNTLYPGCVADTALFRDTPQAFRTIFPWFQKNVTKGYVSQPLAGDRTA : 270
OQW73116     : GLEAGFKAPISMINGKEFKAGKAYKDSKLCTMMMNRELHARYHAQTGIIFSTLYPGCVAETPLFRHAPPLFQKIFPWFQKNITKGYVSQPLSGDRVA : 264
OQW77747     : GLAAGFKAPIAMIDGKPFKGGKAYKDSKLCTMMMSRELHARYHAQTGIIFSTLYPGCVADTPLFRNAPKLFQKIFPWFQKNITKGYVSQPLSGDRVA : 264
OYY63160     : GFEAGFKAPIAMIDGKPFKAGKAYKDSKLCTMMMSRELHARFHDKSGIIFSTLYPGCVAETPLFRNAPKLFQKIFPWFQKNITKGYVSQPLSGDRVA : 264
OYW22568     : GFEAGFKAPIAMIDGKPFKAGKAYKDSKLCTMMMSREFHARFHDKSGIIFSTLYPGCVAETPLFRNAPKLFQKIFPWFQKNITKGYVSQPLSGDRVA : 264
OYX39646     : GFEAGFKAPIAMIDGKPFKAGKAYKDSKLCTMMMSREFHARFHDKSGIIFSTLYPGCVAETPLFRNAPKLFQKIFPWFQKNITKGYVSQPLSGDRVA : 264
SLN77620     : GLKAGFKAPVAMIDGKPFKAGKAYKDSKLGTMVMSRELHTRFHDETGIVFSTLYPGCVADTPLFRNAPPLFQKVFPWFQKNITKGYVSQSLSGERVA : 264
OYU15985     : GLEAGFQDPIAMIDGKPFKPGKAYKDSKLCTMIMSRELHRRFHAETGIIFSTLYPGCVAETPLFRHAPPLFRKIFPWFQKNITKGYVSQPLSGERVA : 264
PZN94637     : GLAAGFRAPVAMIDGKNFKPGKAYKDSKLCTMIMSRELHRRFHADTGIVFATLYPGCVAETPLFRHAPPLFRKIFPWFQKNITRGYVSQPLSGERVA : 264
ODS93018     : GFEAGFLPPIAMIDGKAFKPGKAYKDSKLALMMMSRELHARYHASTGIIFATLYPGCVADTPLFRHAPKAFRTIFPWFQKNITKGYVSQPLSGERVA : 268
PIT82811     : GLEQGFHEPIAMIDGKAFKAGKAYKDSKLCNMIISREMHRRYHDSTGLIFSTVYPGCVADTALFRDTPQAFQTIFPWFQKNITKGYVTQTLAGQRVA : 274
PUE50165     : GLEQGFLDPVAMIDAKPFKAGKAYKDSKLCNMIISRELHRRFHESTGLVCSTVYPGCVADTALFRDTPLAFQRIFPWFQKNITKGYVSQALAGERVA : 282
SIR58578     : GLEAGFRAPVAMIDGKKFKPGKAYKDSKLCNMIISREFHRRYHQNTGIIFNTLYPGCVADTALFRDAPKLFQTIFPWFQKTITKGYVSQALAGTRVA : 277
PHX65690     : GLEKGFRAPIAMINGKPFKPGKAYKDSKLCNMIISRELHRRFHDRTGIVFNTLYPGCVADTALFRDTPAAFQRIFPWFQKNITKGYVTQELSGDRVA : 262
PZO15167     : GLEAGFKAPVAMIDGKPFKPGKAYKDSKLCNMIMSRELHRRYNADTGIVFNTLYPGCVADTALFRDTPQAFRTIFPWFQKNITKGYVTQALAGERVA : 274
WP_086617938 : GLEAGFRAPIAMIDGKPFKPGKAYKDSKLALMMMSRELHARFHAATGIVFATVYPGCVADTPLFRHAPRAFQAIFPWFQKNITKGYVSQPLSGERVA : 268
```

```
                         *        320         *        340         *        360         *
AnLPOR       : QVVADDAFA-QSGVHWSWGNRQLPSRDAFSQPLSSAANDAARAARLWELTAELVGRTPPTG--------326 : 326
DsLPOR       : MVVADPEFA-QSGVHWSWGNRQREGRSAFAQGLSTKATDAARSAELWELTAALTGLTTPAEPVAA----328 : 328
EbLPOR       : DVVADPAFA-RSGVHWSWGNRQREGRAAFAQSLSPRATDAARARELWDLTARLVGLEERVR-EPA----327 : 327
ElLPOR       : DVVADPAFA-RSGVHWSWGNRQREGREAFAQSLSPKATDAARARELWDLTARLVGLEQRVR-EPA----327 : 327
GpLPOR       : QVVADPAFGSRSGVHWSWGNRQKEGREAFAQQLTARAQDQALGERLWTLTAGCVGMGS-----------322 : 322
LfLPOR       : QVVTDPDFA-RSGVHWSWGNRQEQGRKAFSQSLSSKATDTTRSETLWNLSAALVRIDVSGRPL------326 : 326
LvLPOR       : QVVADPGFS-QSGVHWSWGNRQREGRKAFSQGLSIKATDAERSRELWDLTAGLVGLN------------320 : 320
PdLPOR       : QVVADPDFT-RSGVHWSWGNRQKPGAQAFAQPLSSKAENAHRAARLWDLSAKLVGLEDARIGEPA----332 : 332
SaLPOR       : QVIADPAFASQSGVHWSWGNRQKEGRMPFAQPLSTRGTNEARNERLWDLTMKAVGLA------------323 : 323
SgLPOR       : QVVADPEFT-RSGVHWSWGNRQKVGQVAFAQSMSDKATDAARSKELWDLSAGLVCL-------------319 : 319
SlLPOR       : QVIADPAFASQSGVHWSWGNRQKQGRMPFAQPLSARGTNEARNERLWDLTLKAVGLA------------327 : 327
SpLPOR       : QVVADPGFT-QSGVHWSWGNRQQEGREAFAQSLSSKATDASRSQRLWALSAGLVGLASDKQPARSVVAA332 : 332
OWS70994     : QVVADPHFT-QSGVHWSWGNRNSAIREPFAQELSMKAKSEKLSKDLWEITAKLVGMPTD----------327 : 327
AWW49216     : QVVADLRFT-QSGVHWSWGNRNSAIREPFAQELSMKAKSEKLSKDLWEITAKLVGMPSD----------327 : 327
OYQ31188     : QVVADPAFASQSGVHWSWGNRQKEGRMPFAQPLSARATLEARNERLWDLTMKAVGLA------------333 : 333
OSZ70988     : QVVADPAFASQSGVHWSWGNRQKEGRMPFAQPLSARATLEARNERLWDLTMKAVGLA------------328 : 328
OYX79084     : QVIAEPAFASQSGVHWSWGNRQKQGRMPFAQPLSARGTNEARNERLWDLTLKAVGLA------------327 : 327
OYZ16473     : QVIADPAFASQSGVHWSWGNRQKQGRMPFAQPLSARGTNEARNERLWDLTLKAVGLA------------327 : 327
OQW73116     : QVVADDGFT-QSGVHWSWGNRQQLGRAAFAQGLSSKATDIGRSQRLWALSATLVGLG------------320 : 320
OQW77747     : QVVADPGFT-QSGVHWSWGNRQQEGREAFAQSLSSKATDASRSQRLWALSAGLVGLASDKQPARSVVAA332 : 332
OYY63160     : QVVADPDFT-QSGVHWSWGNRQQEGRAAFAQSLSSKATDTTRSQRLWALSERLVGLA------------320 : 320
OYW22568     : QVVADPDFT-QSGVHWSWGNRQQEGRAAFAQSLSSKATDTTRSQRLWALSERLVGLA------------320 : 320
OYX39646     : QVVADPDFT-QSGVHWSWGNRQQEGRAAFAQSLSSKATDTTRSQRLWALSERLVGLA------------320 : 320
SLN77620     : QVVADPGFS-QSGVHWSWGNRQREGRKAFSQGLSIKATDAERSRELWDLTAGLVGLN------------320 : 320
OYU15985     : QVVADPGFI-RSGVHWSWGNRQKAGRQAFAQPLSAKANDQRRAARLWDLTARLTGLHAQS---------323 : 323
PZN94637     : QVVADPAFT-ASGVHWSWGNRQRAGREAFSQPLSAKANDQARAARLWDLTAKLTGLEA-----------321 : 321
ODS93018     : QVVADPQFT-RSGVHWSWGNRQKPGAEAFAQPLSAKAENARRSARLWDLSLRLVGLDDPAAVQQAA---333 : 333
PIT82811     : QVVVDKDFS-TSGVHWSWGNRQVPGRQAFAQPLSAKATQLQLGIRLWELSNQLVGIAD-----------331 : 331
PUE50165     : QVVVDADFS-TSGVHWSWGNRQRPGRPAFAQKLSAKATQESLGIRLWALSEKLTGLSA-----------339 : 339
SIR58578     : QVVADDAFA-QSGVHWSWGNRQLPSRDAFSQPLSSAANDAARAARLWELTAELVGRTPPTG--------337 : 337
PHX65690     : QVVADDAFA-ESGVHWSWGNRQ-NGRAAFAQPLTAKAQQVTRAARLWTLSEQMVGLPPSE---------320 : 320
PZO15167     : QVVADETFA-QSGVHWSWGNRQQAGRQAFAQPLSAKASDVARASRLWNLSEKLVGLA------------330 : 330
WP_086617938 : QVVADPAFT-ASGVHWSWGNRQKPGAKAFAQPLSAKAENQRRAARLWDLSAALVGLAQPAHREPA----332 : 332
```

Figure S1: Multiple sequence alignment of all putative LPORs identified outside of oxygenic phototrophic genera. Sequences are identified by LPOR ID (see Figure 1 in the main manuscript, supplementary Table S1) or by their UniProt or Genbank ID (see supplementary Table S1). Two identical LPOR sequences were identified for two *Erythrobacter litoralis* strains, *Erythrobacter* sp. HL-111 and *Erythrobacteraceae* bacterium HL-111 as well as for two *Porphyrobacter dokdonensis* strains. For those organisms only one sequence was included in the alignment (see supplementary Table S1). Highlighted positions are: the conserved NADPH-binding motif (shown in red) (Buhr, et al. 2008), four conserved cysteine residues supposed to be involved in substrate binding (in green) (Menon, et al. 2010) and the catalytic Tyr and Lys residues (in blue) (Menon, et al. 2009).

1.2 *In vitro* and *in vivo* LPOR activity tests

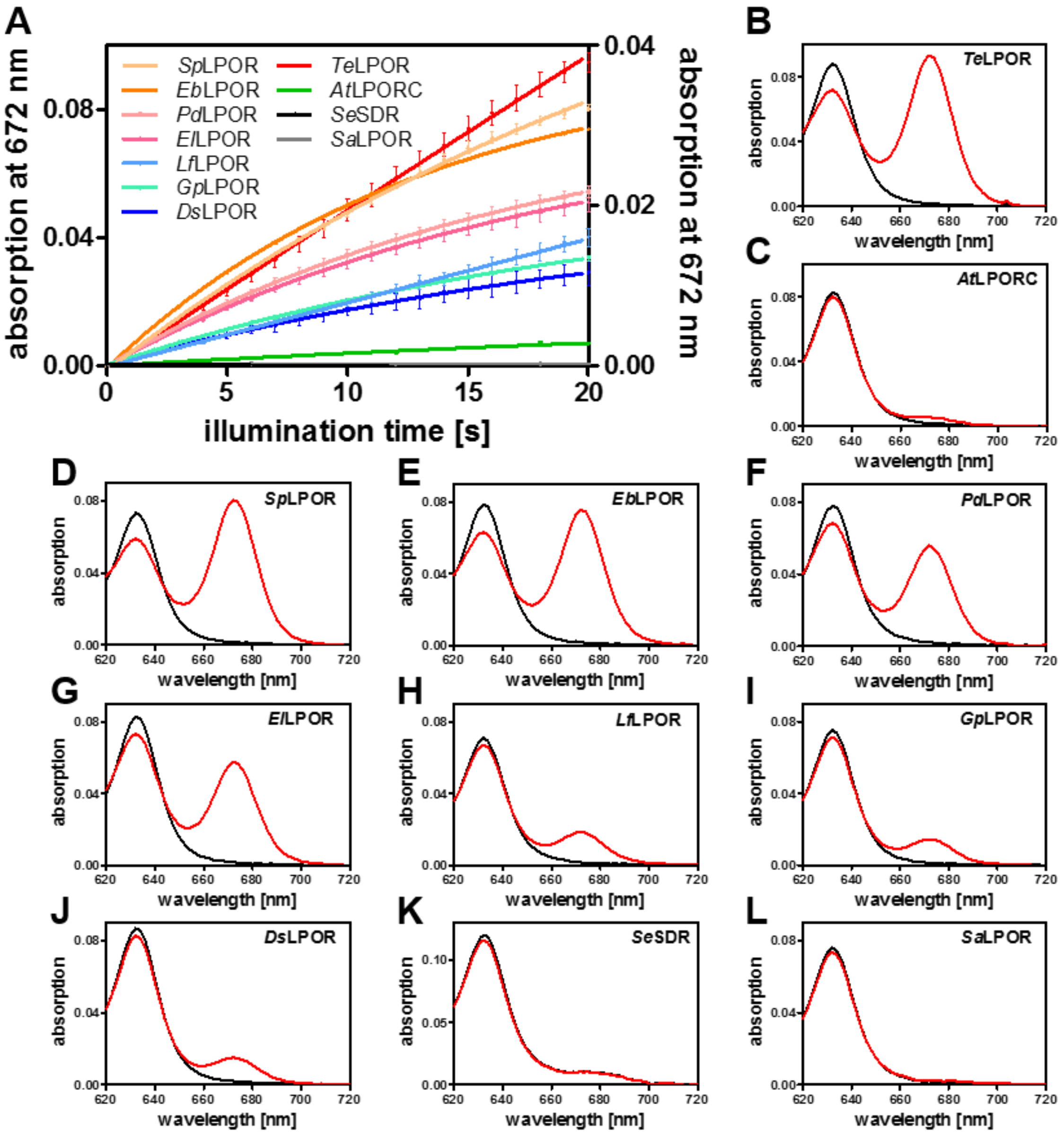

Figure S2: Light-dependent *in vitro* conversion of Pchlide by purified enzymes. Enzymes are identified by LPOR ID (supplementary Table S1, Figure 1 in the main manuscript). (A) Light-dependent Chlide formation catalyzed by LPOR enzymes. To follow the reaction over time, the change in absorption at 672 nm was plotted against the illumination time. Since the different enzymes showed variably high activities, the data is shown on two differently scaled Y-axes (*Te*LPOR, *Sp*LPOR, *Eb*LPOR, *Pd*LPOR, *El*LPOR, left ordinate; *Lf*LPOR, *Gp*LPOR, *Ds*LPOR, SeSDR, *Sa*LPOR, *Se*SDR and *At*LPOR right ordinate). Error bars represent the standard deviation of the mean derived from three independent measurements. (B-L) UV/Vis spectrophotometric proof of the Chlide formation. Depicted is the conversion of Pchlide (black line; without illumination) to Chlide (red line; after 20 s of illumination; *At*LPORC 24 s of illumination).

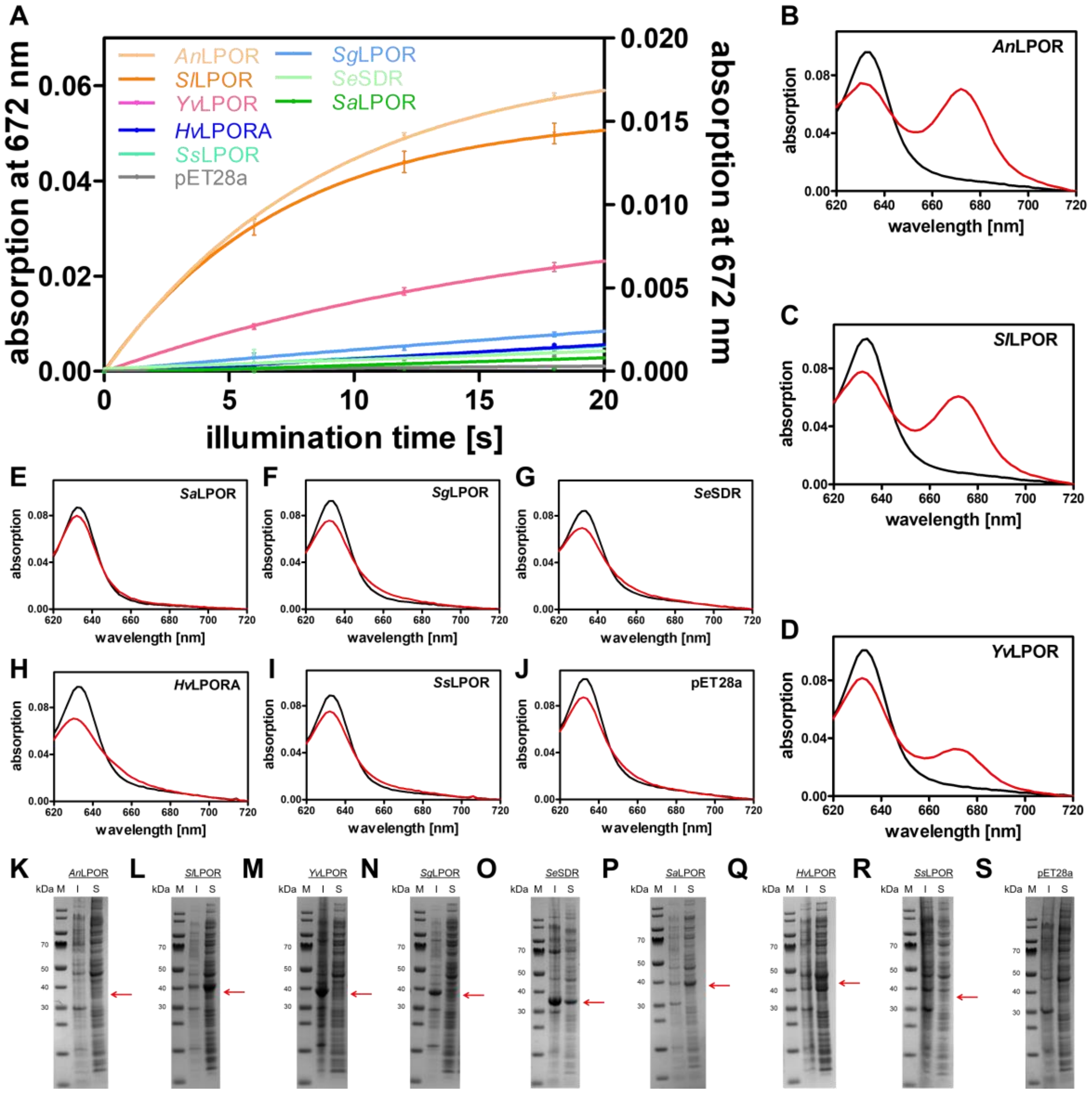

Figure S3: Analysis of the light-dependent *in vitro* conversion of Pchlide by cell free lysates. Enzymes are identified by LPOR ID (supplementary Table S1, Figure 1 in the main manuscript). (A) Absorption changes at 672 nm with illumination time indicate Pchlide to Chlide turnover. For all putative LPORs, which could not be purified by immobilized metal ion affinity chromatography, light-dependent Pchlide turnover was examined using cell-free extracts. 1 g cells (10% (w/v) wet cells in 500mM NaCl, 20% Glycerin, 20 mM Tris, pH = 7.5 buffer were lysed. Unbroken cells and cell debris were removed by centrifugation, and 250 µL of the respective cell-free extract was used for the *in vitro* LPOR activity assay. Since the different cell free lysates showed variably high activities, the data is shown on two differently scaled Y-axes (*An*LPOR, *Sl*LPOR, and *Lv*LPOR, left ordinate; *Hv*LPOR, *Ss*LPOR, *Sg*LPOR, *Sa*SDR, *Sa*LPOR, *Se*SDR and empty vector control (pET28a), right ordinate). Error bars represent the standard deviation of the mean derived from three independent measurements. (B-J) UV/Vis spectrophotometric proof of the Chlide formation. Depicted is the conversion of Pchlide (black line; without illumination) to Chlide (red line; after 20 s of illumination). LPORs identified by LPOR ID (supplementary Table S1) as indicated in the Figure. Panel J, shows the empty vector negative control. (K-S) 12 % SDS-PAGE analysis of the insoluble (I) and the soluble (S) protein fraction obtained by centrifugation after cell lysis of the respective expression cultures. The red arrows mark the expected theoretical molecular mass of the putative LPORs: *An*LPOR: 37,159 Da, *Sl*LPOR: 37,481 Da, *Yv*LPOR: 36,572 Da, *Sg*LPOR: 37,006 Da, *Se*SDR: 34,644 Da, *Sa*LPOR: 37,155 Da, *Hv*LPORA: 43,345 Da, *Ss*LPOR: 37,824 Da. Please note that for *An*LPOR, *Yv*LPOR and *Sg*LPOR no strong overexpression was observed. With the exception of *Sg*LPOR, LPOR activity was nevertheless detected for the corresponding cell free lysates.

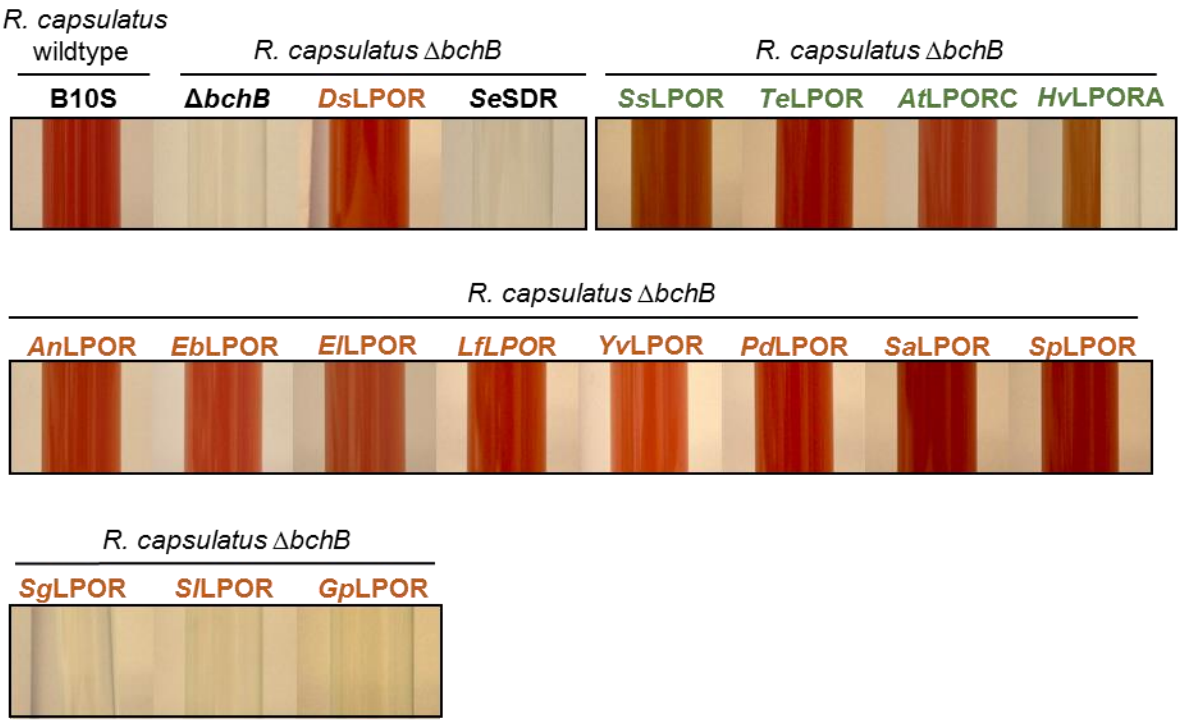

Figure S4: *Rhodobacter capsulatus*-based LPOR *in vivo* assay for all selected AAPB LPORs. Growth phenotype of *R. capsulatus* wildtype and the Δ*bchB* (DPOR deficient) strain complemented with different known and putative LPORs. First panel: The *R. capsualtus* wildtype strain B10S (black) harboring the intrinsic DPOR for BChl *a* formation was used as reference strain for phototrophic growth. The DPOR-deficient *R. capsulatus* mutant strains Δ*bchB* as well as the Δ*bchB* strain complemented with *Se*SDR were used as respective negative controls (black). Known LPORs from AAPB (orange, *Ds*LPOR) and cyanobacteria/plant (green, *Ss*LPOR, *Te*LPOR, *At*LPORC and *Hv*LPORA) have been used as corresponding LPOR positive controls. Second and third panel: For AAPB LPORs (orange), growth of red-colored cultures indicate *in vivo* LPOR activity. LPORs identified by LPOR ID (supplementary Table S1) as indicated in the Figure.

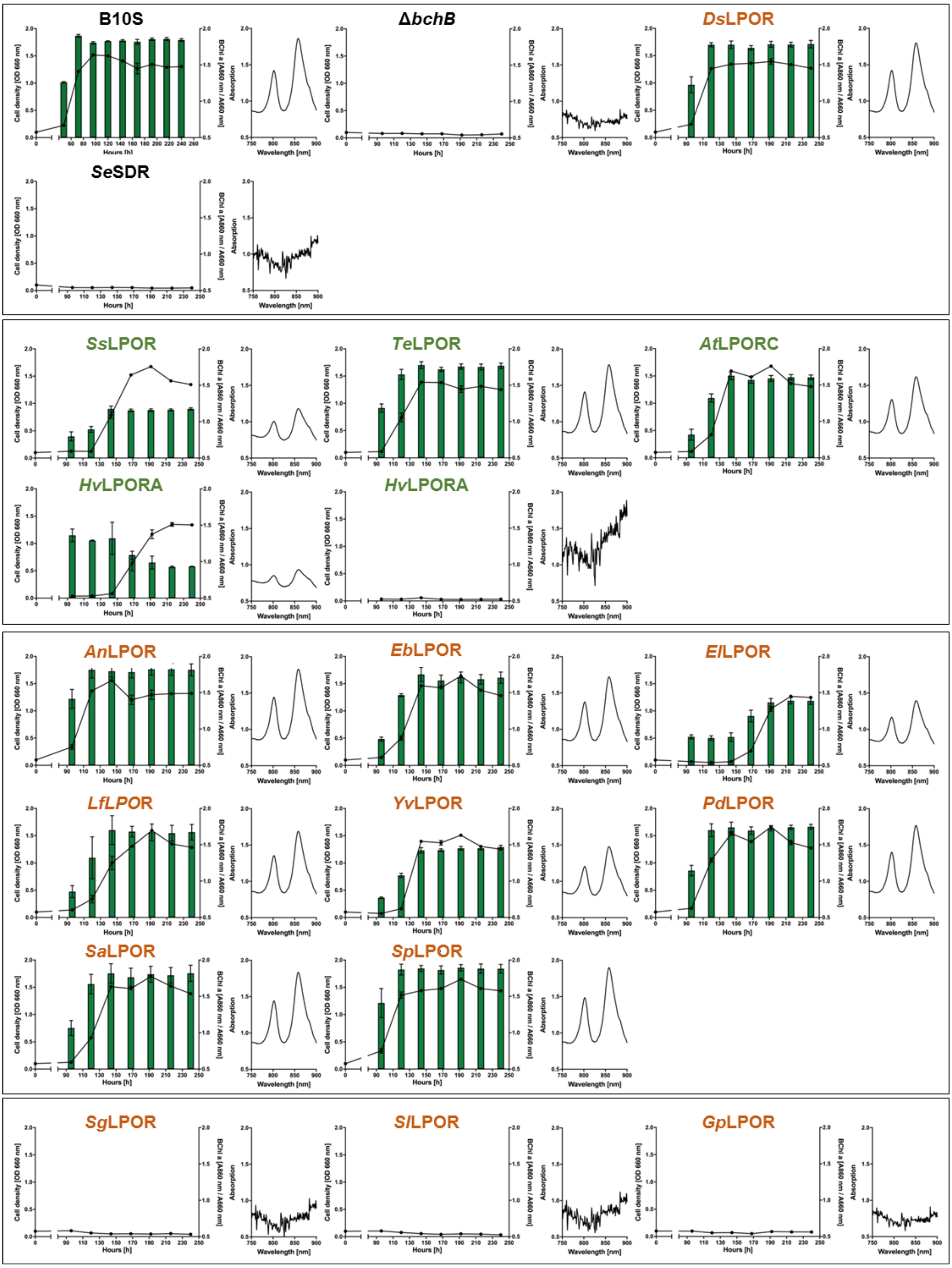

Figure S5: *Rhodobacter capsulatus*-based LPOR *in vivo* assay for all selected AAPB LPORs. Growth curves and BChl*a* accumulation, measured as *in vivo* (whole cell) absorption at 860 nm (green bars) over time of cultivation, normalized BChl ***a***-dependent absorption (black curves; OD_{860}/OD_{660}) as well as absorption spectra of cellular BChl*a* after 240 h of cultivation are shown. Assays were carried out for the same set of LPOR enzymes as shown in supplementary Figure S4. Data represent mean values and respective standard deviations from three independent measurements. LPORs identified by LPOR ID (supplementary Table S1) as indicated in the Figure.

Table S4: Activity comparison of AAPB LPORs by *in vivo* and *in vitro* assays. Activity comparison by color (light red: inactive; shades of green: active) and expressed accordingly as: absent (-) low (+), medium(++) and high activity (+++). LPORs identified by LPOR ID (Figure 1 in the main manuscript). Differential, semi-quantitative assessment relative to the most active LPOR(*Eb*LPOR: 0.69 ± 0.01 U/mg) (*in vitro*) or relative to the BChl *a* levels of *R. capsulatus* B10S wildtype (*in vivo*); *in vitro* assessment: (-) no activity, (+) <50 % activity, (++) 50-90 % activity, (+++) >90 % activity, all crude-cell extract activities assigned as (+); *in vivo* assessment: (-) no BChl *a* accumulation, (+) ≤ 75 % BChl *a* accumulation, (++) 75-90 % BChl *a* accumulation, (+++) ≥ 90 % BChl *a* accumulation.

LPOR protein	class, order	*in vitro* activity	*in vivo* activity
*Eb*LPOR	α-Proteobacteria, *Sphingomonadales*	+++	++
*Sp*LPOR	α-Proteobacteria, *Sphingomonadales*	+++	+++
*Pd*LPOR	α-Proteobacteria, *Sphingomonadales*	++	+++
*El*LPOR	α-Proteobacteria, *Sphingomonadales*	++	+
*Lf*LPOR	α-Proteobacteria, *Rhodobacterales*	+	++
*Gp*LPOR	FCB group bacteria, *Gemmatimonadetes*	+	-
*Sl*LPOR[a]	α-Proteobacteria, *Sphingomonadales*	+	-
*Yv*LPOR[a]	α-Proteobacteria, *Rhodobacterales*	+	++
*An*LPOR[a]	α-Proteobacteria, *Rhodospirillales*	+	+++
*Sa*LPOR	α-Proteobacteria, *Sphingomonadales*	-	+++
*Sg*LPOR[a]	α-Proteobacteria, *Rhodobacterales*	-	-
**Reference enzymes**			
*Ds*LPOR	α-Proteobacteria, *Rhodobacterales*	+	+++
*Te*LPOR (positive)	Cyanobacteria, *Synechococcales*	+++	+++
*Ss*LPOR (positive)[a]	Cyanobacteria, *Synechococcales*	-	+
*At*LPORC (positive)	Magnoliopsida, *Brassicales*	+	++
*Hv*LPORA (positive)[a]	Magnoliopsida, *Poales*	-	+ -
*Se*SDR (negative)	Actinobacteria, *Pseudonocardiales*	-	-

[a]: *in vitro* activity measurements performed using crude cell extracts

1.3 Comparative biochemical characterization of AAPB, plant and cyanobacterial LPORs.

To elucidate whether the identified AAPB LPORs possess biochemical properties distinct from their plant and cyanobacterial counterparts, we biochemically characterized seven of the newly identified AAPB LPORs (*El*LPOR, *Eb*LPOR, *Pd*LPOR, *Gp*LPOR, *Sp*LPOR, *Lf*LPOR and *Ds*LPOR) comparatively to the plant (*At*LPORC) and cyanobacterial (*Te*LPOR) enzyme. This selection was largely dictated by the possibility to obtain the respective enzyme in sufficient quantities and comparable purity. As parameters we determined pH- and temperature activity optima, temperature stabilities (temperature-dependent unfolding), the K_d of the ternary NADPH/Pchlide/LPOR holo-protein complex and the preference for either monovinyl (MV)- and divinyl (DV)-Pchlide substrates.

1.3.1 pH activity optima and range

The pH-dependent activity of the above listed LPORs was measured using three different buffer systems to cover the pH range from pH = 5.5 to 10. For the pH range from pH = 5.5 to 7.5 a sodium phosphate buffer was used. The pH range from pH = 7.5 to 9.0 was covered using Tris buffer, and for the pH range from pH = 9.0 to 10.0 a glycine buffer was employed. At the boundaries of the pH range of the respective buffer, e.g. at pH = 7.5, the activity was measured in both sodium phosphate buffer and Tris buffer, to correct for any pH-independent effect of the employed buffer system (supplementary Figure S6). This allowed for the determination of the pH activity optimum of the different enzymes. As an additional parameter, we determined the 80% pH optimum range, defined as all obtained values around the optimum with values > 80% of the pH optimum. (supplementary Figure S6, supplementary Table S5 and Table 1 of the main manuscript). The 80% pH optimum threshold is indicated by a grey line in supplementary Figure S6.

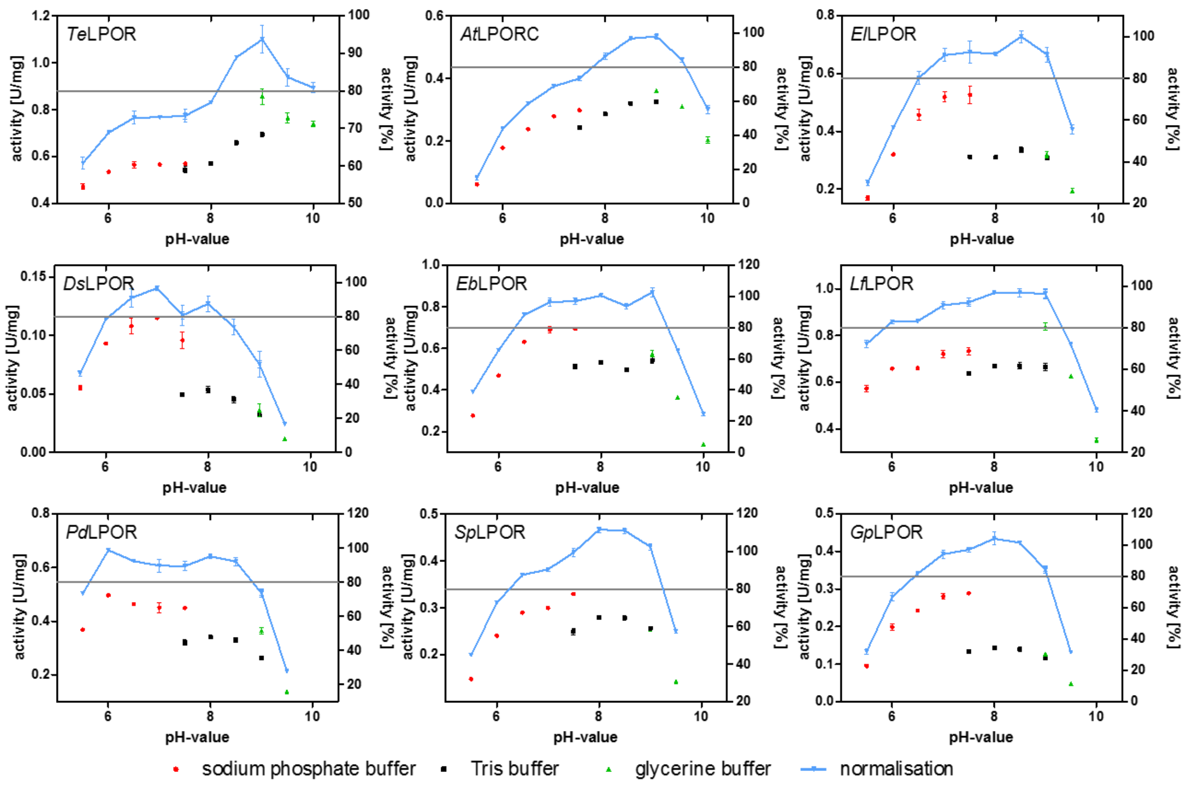

Figure S6: pH activity optima determination. Enzymes are identified by LPOR ID (supplementary Table S1). Activity data obtained using sodium phosphate buffer (pH = 5.5 to 7.5, red data), Tris buffer (pH = 7.5 to 9.0, black data) and glycine buffer (pH = 9.0 to 10.0, green data); scaled on the left ordinate. To compare the activity using different buffers the specific activity was normalized (blue squares and line); scaled on the right ordinate. The grey lines illustrate the 80% normalized activity threshold used to infer the 80% pH-optimum range. All measurements were carried out at least in triplicate with the error bars corresponding to the standard deviation of the mean of independent measurements.

Table S5: pH activity optima and 80% pH-optimum range determined for the AAPB LPORs, *Te*LPOR and *At*LPORC. Enzymes are identified by LPOR ID (supplementary Table S1).

	***El*LPOR**	***Eb*LPOR**	***Pd*LPOR**	***Gp*LPOR**	***Sp*LPOR**	***Lf*LPOR**	***Ds*LPOR**	***Te*LPOR**	***At*LPORC**
$pH_{optimum}$ [pH]	8.5	9.0	6.0/8.0[a]	8.0	8.0	9.0	7.0	8.5	8.5
$pH_{optimum}$ range [pH units]	2.5	2.5	2.5	2.5	2.5	3.0	2.0	1.5	1.5

[a]: the pH optima curve of PdLPOR shows two maxima with very similar values at pH 6.0 and pH 8.0

All of the analysed LPORs possess pH activity optima between pH = 7.0 and 9.0, which is very similar to the pH optimum determined previously for the LPOR of *Chlamydomonas reinhardtii* (optimum at pH = 8.5) isolated from different *C. reinhardtii* mutant strains (Ford, et al. 1983). Compared to the plant and cyanobacterial enzymes (*Te*LPOR and *At*LPORC) all AAPB LPORs show a broader 80% pH optimum range (supplementary Table S5).

1.3.2 Temperature-activity optima, optimum temperature range and temperature dependent unfolding

Temperature-activity optima and optimum temperature range.

To determine the temperature-activity optima of the different AAPB, plant and cyanobacterial LPORs the temperature-dependent activity of the enzymes was measured in the temperature range between 10°C and 55°C. All measurements were performed as described in the Materials and Methods section. The corresponding plots are shown in supplementary Figure S7, with the 80% temperature-optimum threshold marked by a grey line. To verify that the temperature stayed constant during the activity measurement, and is not or only minimally influenced by the LED light source that was used to drive the reaction, the sample temperature was determined before and after the measurement using mock samples without enzyme. The corresponding experimentally determined sample-temperature variance is shown as x-error in supplementary Figure S7. The resulting values are summarized in supplementary Table S6 (also shown in Table 1 of the main manuscript). Among the tested LPORs, the cyanobacterial *Te*LPOR possesses, with an optimum value of ~36°C, the highest temperature-activity optimum, followed by the plant *At*LPORC enzyme, which possesses a temperature optimum at ~27°C. The high optimum temperature of the *Te*LPOR enzyme is not surprising, since the enzyme originates from the thermophilic cyanobacterium *Thermosynechococcus elongatus* (McFarlane, et al. 2005). Previously, an even higher temperature-activity optimum of ~50°C has been reported for this enzyme (McFarlane, et al. 2005). The discrepancy hereby likely originates from the different measuring conditions that were employed here. All analysed AAPB LPORs show significant lower temperature optima between ~16°C (*Gp*LPOR, *Ds*LPOR) and 23.3°C (*El*LPOR, *Sp*LPOR, *Lf*LPOR).

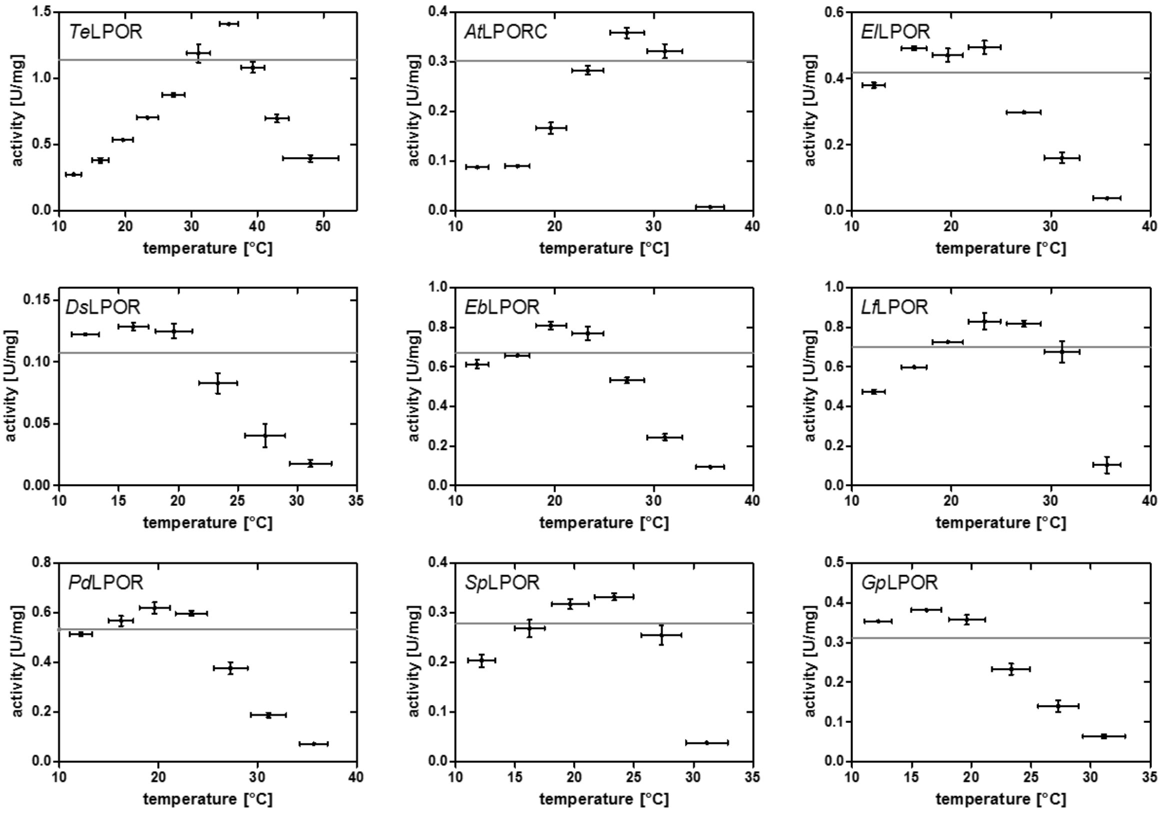

Figure S7: Temperature activity optima. Enzymes identified by LPOR ID (supplementary Table S1). The grey lines illustrate the 80% of activity threshold. All measurements were carried out at least in triplicate with the error bars corresponding to the standard deviation of the mean of independent measurements.

Table S6: Temperature-activity optima and 80% optimum-temperature range determined for AAPB LPORs, *Te*LPOR and *At*LPORC. Enzymes are identified by LPOR ID (supplementary Table S1).

	***El*LPOR**	***Eb*LPOR**	***Pd*LPOR**	***Gp*LPOR**	***Sp*LPOR**	***Lf*LPOR**	***Ds*LPOR**	***Te*LPOR**	***At*LPORC**
$T_{optimum}$ [°C]	23.3 ± 1.6	19.6 ± 1.5	19.6 ± 1.5	16.2 ± 1.3	23.3 ± 1.6	23.3 ± 1.6	16.2 ± 1.3	35.7 ± 1.4	27.3 ± 1.7
$T_{optimum}$ range [°C]	7.1	3.7	7.1	7.4	3.7	7.7	7.4	4.6	3.8

With the exception of *Eb*LPOR and *Sp*LPOR, all AAPB LPORs show a broader 80% temperature-optimum range. This observation, along with the above described broader pH-optimum range of the AAPB LPOR enzymes, could related to the marine habitat in which the host organisms containing the identified AAPB LPORs are found. Marine environments are hereby characterized by a high degree of complexity, i.e. experiencing larger fluctuation with regard to growth-relevant environmental parameters such as temperature, pH, pressure, nutrient availability and intensity of the available light as well as light quality (wavelength) (Zhang and Kim 2010).

Thermal stability as determined by thermal unfolding studies.

As a proxy for thermostability, we also determined the thermal unfolding behavior of the different AAPB, plant and cyanobacterial LPORs, using differential scanning fluorimetry (DSF), employing a NanoTemper Prometheus NT-Plex instrument (NanoTemper GmbH,

Munich, Germany). DSF relies on the change of the intrinsic protein fluorescence (Tyr and Trp residues) due to thermal unfolding of the protein. All measurements were performed as described in the Materials and Methods. When the change in the fluorescence signal is plotted versus the temperature (which is increased incrementally during the measurement using a fixed temperature gradient) typical unfolding-, also called melting-curves are obtained (supplementary Figure S8). From the first derivative of this data, the melting temperature (T_M), defined as the temperature at which 50% of the molecules are unfolded, can be determined (highlighted as dashed line in supplementary Figure S8, supplementary Table S7 and Table 1 of the main manuscript).

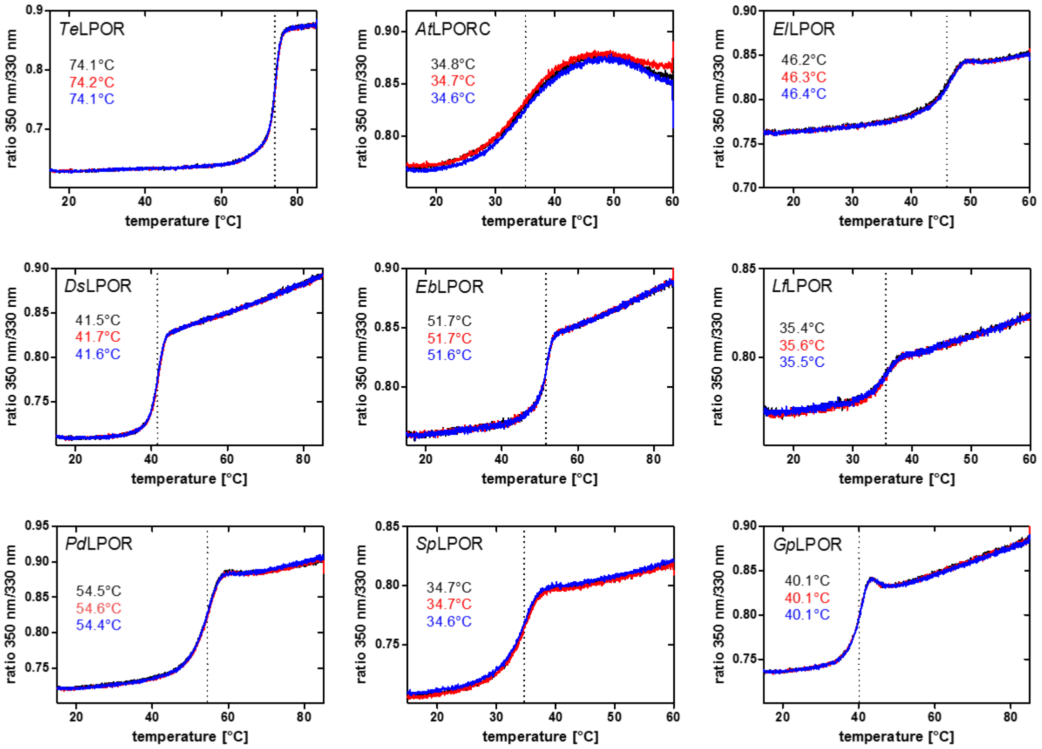

Figure S8: Temperature-dependent unfolding studies. Enzymes are identified by LPOR ID (supplementary Table S1). Temperature-dependent unfolding was determined using DSF by plotting the ratio of the fluorescence emissions of 350 nm to 330 nm against the temperature. Each plot depicts three replicate analyses. The average Melting temperatures, determined for each curve from the first derivative of the data are given in each panel and the mean of the three melting-temperatures is shown as dashed line.

Table S7: DSF-derived melting-temperatures determined for AAPB LPORs, *Te*LPOR and *At*LPORC. Enzymes are identified by LPOR ID (supplementary Table S1).

	***El*LPOR**	***Eb*LPOR**	***Pd*LPOR**	***Gp*LPOR**	***Sp*LPOR**	***Lf*LPOR**	***Ds*LPOR**	***Te*LPOR**	***At*LPORC**
T_M [°C]	46.3 ± 0.09	51.6 ± 0.04	54.4 ± 0.06	40.1 ± 0.09	34.7 ± 0.05	35.6 ± 0.07	41.6 ± 0.07	74.1 ± 0.06	35.1 ± 0.49

As expected, the thermophilic *Te*LPOR enzyme shows, with a value of ~74°C, the highest T_M of all analysed enzymes. All other enzymes display T_M values between ~35°C and ~55°C, with the *Pd*LPOR enzyme possessing the highest melting temperature among all tested AAPB

LPORs. Based on this data no general trend can be identified that differentiates AAPB LPORs from their plant and cyanobacterial counterparts with regard to their thermal stability.

1.3.3 Dissociation of the NADPH/Pchlide/LPOR ternary complex

The dissociation constant (K_d) of the ternary NADPH/Pchlide/LPOR holo-protein complex was determined by following the red shift of the Q_y absorbance band from ≈ 630 nm (free Pchlide) to ≈ 642 nm (ternary complex) (Heyes, et al. 2008; Menon, et al. 2016) as described in the Materials and Method section. Please note that, for all measurements Pchlide preparations were used that were obtained from *R. capsulatus* ZY5, which consist of a mixture of monovinyl (MV)- and divinyl (DV)-Pchlide (see Supplementary Materials section 1.3.4). All measurements were performed with and without 70 mM DTT as reducing agent. The addition of DTT is supposed to maintain LPOR cysteine residues, some of which have been implicated in either Pchlide binding or catalysis (Heyes, et al. 2000; Buhr, et al. 2008), in their reduced form. For the LPOR of *Synechocystis* sp. (*Ss*LPOR), it was shown that the addition of the reducing agent β-mercaptoethanol is necessary for enzyme activity (Heyes, et al. 2000). For all analysed LPORs, a clear red shift of the Q_y band due to ternary complex formation was observed (supplementary Figure S9/S10; A panels), which however appears to be of different magnitude depending on the analysed LPOR. This already suggests crossly different K_d values for some of the LPORs. These sequences of spectra were decomposed into species spectra of free and bound Pchlide and the corresponding mole-fraction vs. protein concentration profiles. The latter profiles were globally fitted by a simple one to one binding model allowing the determination of the K_d value of the NADPH/Pchlide/LPOR ternary complex (supplementary Figure S9/S10; B panels). Please note that, for most of the AAPB LPORs no complete saturation was reached (supplementary Figure S9/S10; B panels; compare e.g. to *Te*LPOR and *At*LPORC). All obtained K_d values are summarized in supplementary Table S8 and are given in Table 1 of the main manuscript. From the data it is directly apparent, that irrespective of the presence of the reducing agent DTT, all AAPB LPORs possess higher K_d values (between ~6 µM and ~130 µM) as compared to the cyanobacterial *Te*LPOR and the plant *At*LPORC (~2 µM for both enzymes). Among the AAPB LPORs, *Sp*LPOR and *Lf*LPOR are exceptions, showing K_d values below 10 µM, while all other AAPB LPORs possess K_d values between ~40 µM (*Pd*LPOR) and ~130 µM (*Gp*LPOR). Moreover, DTT seems to play different roles for the assembly/dissociation of the ternary complex for the AAPB LPOR enzymes and their cyanobacterial and plant counterparts. While for both *Te*LPOR and *At*LPORC no influence of DTT was observed (Table 1 of the main manuscript, supplementary Table S8, K_d (+DTT/-DTT)), most AAPB LPORs either show higher (*Gp*LPOR, *Ds*LPOR) or lower (*El*LPOR, *Eb*LPOR) K_d values in the presence of DTT indicating that in some of the analysed LPORs cysteine residues might be involved in substrate binding, as suggested before for *Ss*LPOR (Heyes, et al. 2000). For *Pd*LPOR, *Sp*LPOR and *Lf*LPOR no, or only a negligible influence of DTT was observed.

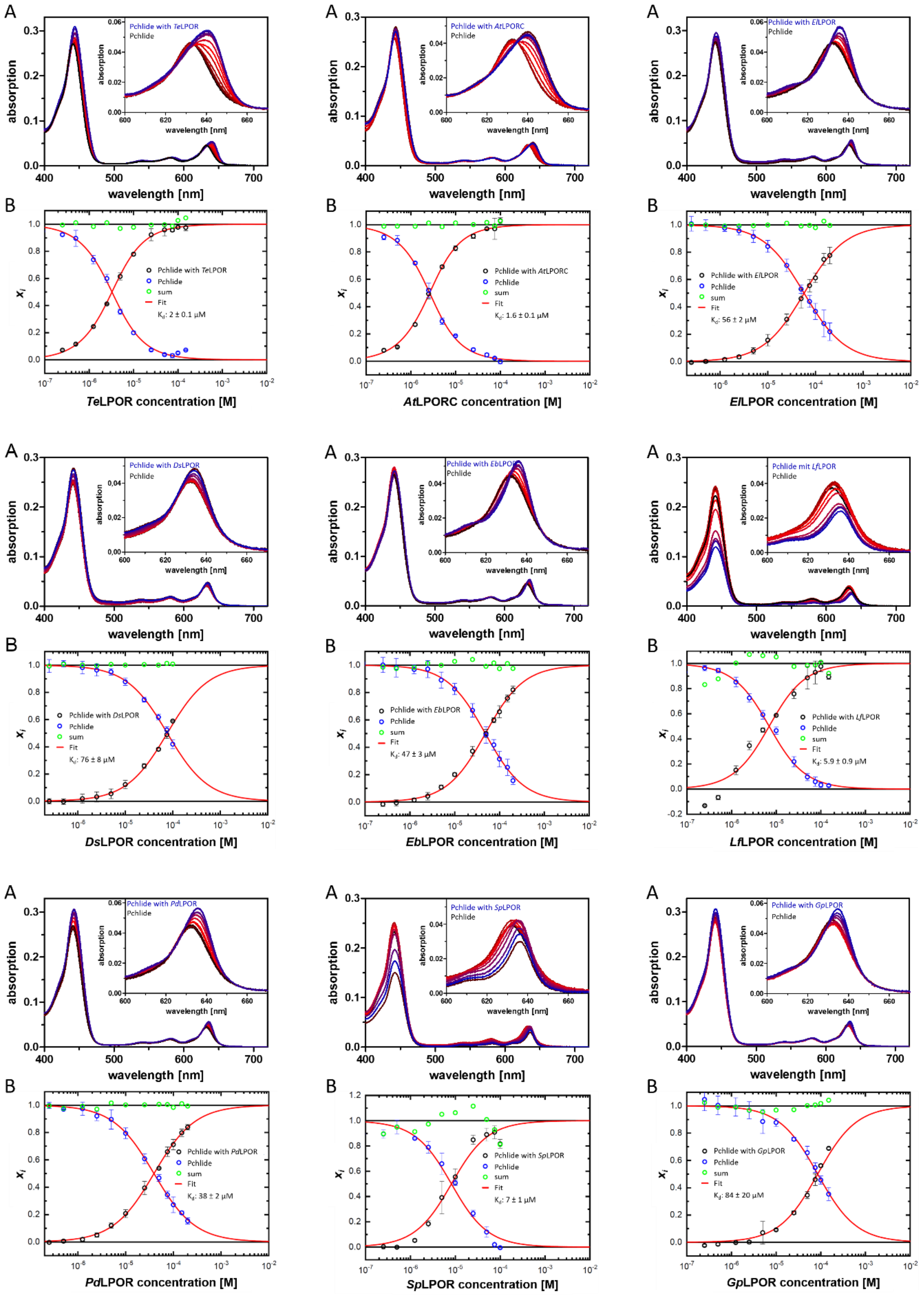

Figure S9: K_d determination for the ternary NADPH/Pchlide/LPOR complex without DTT. Absorption spectra (A) and data analysis (B) used to determine K_d. Enzymes are identified by LPOR ID supplementary Table S1). Error bars correspond to the standard deviation of the mean of three independent measurements.

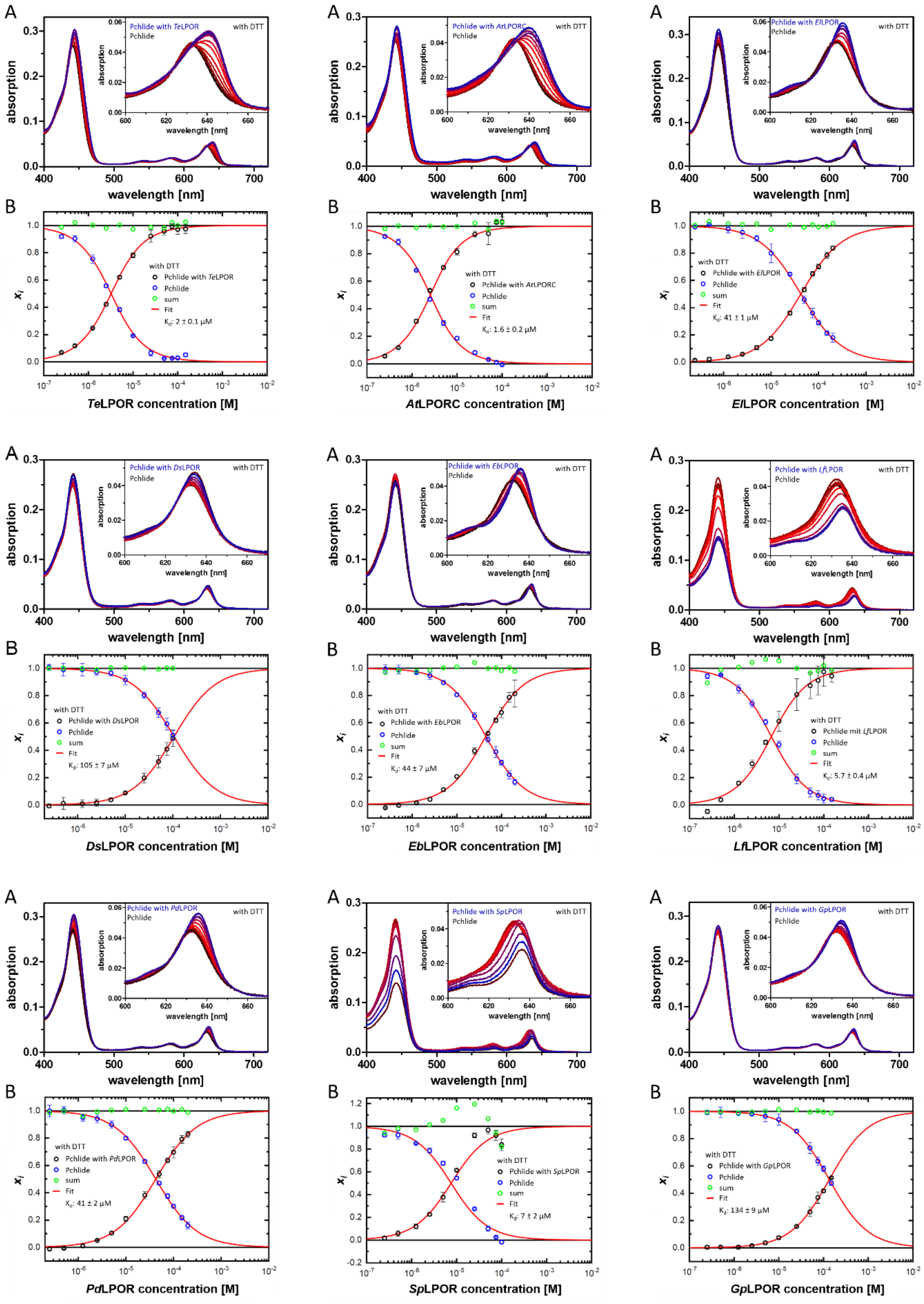

Figure S10: K_d determination for the ternary NADPH/Pchlide/LPOR complex with DTT. Absorption spectra (A) and data analysis (B) used to determine K_d. Enzymes are identified by LPOR ID (supplementary Table S1). Error bars correspond to the standard deviation of the mean of three independent measurements.

Table S8: K_d of the NADPH/Pchlide/LPOR ternary complex determined for AAPB LPORs, *Te*LPOR and *At*LPORC. Enzymes are identified by LPOR ID (supplementary Table S1).

	***El*LPOR**	***Eb*LPOR**	***Pd*LPOR**	***Gp*LPOR**	***Sp*LPOR**	***Lf*LPOR**	***Ds*LPOR**	***Te*LPOR**	***At*LPORC**
K_d (+ DTT)	41 ± 1	44 ± 7	41 ± 2	134 ± 9	7 ± 2	5.7 ± 0.4	105 ± 7	2.0 ± 0.1	1.6 ± 0.2
K_d (- DTT)	56 ± 2	47 ± 3	38 ± 2	84 ± 20	7 ± 1	5.9 ± 0.9	76 ± 8	2.0 ± 0.1	1.6 ± 0.2
K_d (+DTT/-DTT)	0.73	0.94	1.0	1.6	1.0	1.0	1.38	1.0	1.0

1.3.4 MV/DV-Pchlide substrate preference

It is known from literature that the *R. capsulatus* ZY5 strain, that is commonly used for the production of the commercially not available LPOR substrate Pchlide, produces a mixture of monovinyl(MV)- and divinyl(DV)-Pchlide (supplementary Figure S11) (Heyes, et al. 2006). To the best of our knowledge, the acceptance of MV/DV-Pchlide has only been probed for the cyanobacterial *Te*LPOR enzyme, for which no clear preference for either MV- or DV-Pchlide has been reported (Heyes, et al. 2006). Nonetheless, given the different origin of the AAPB LPORs, we analysed the MV/DV-Pchlide substrate preference of the AAPB LPORs comparatively to their plant and cyanobacterial counterparts (*At*LPORC and *Te*LPOR). Since neither MV- nor DV-Pchlide are commercially available, we purified both Pchlide forms from the mixed MV/DV-Pchlide preparations produced by *R. capsulatus* ZY5.

Identification of MV- and DV-Pchlide in R. capsulatus ZY5 Pchlide preparations.

When the Pchlide substrate is purified from *R. capsulatus* ZY5 by solid-phase extraction using C18 material (see Materials and Methods for details), MV- and DV-Pchlide co-purify as a single peak (Heyes, et al. 2006). This mixed Pchlide preparation is then commonly used as substrate for LPORs. MV- and DV-Pchlide can be separated by reverse-phase High Pressure Liquid Chromatography (HPLC) using a C30 column (Heyes, et al. 2006). To verify the presence of MV- and DV-Pchlide in our *R. capsulatus* ZY5 produced Pchlide preparations, we initially performed an analytical HPLC run using an analytical C30 HPLC column (supplementary Figure S11, A)(for experimental details see Materials and Methods). Two species, with a typical Pchlide absorption spectrum, eluted at 22.6 and 27.3 min, respectively (supplementary Figure S11, B). The species eluting at 22.6 min hereby possesses red-shifted Soret and Q_y-bands, compared to the later eluting species (27.3 min)(supplementary Figure S11, B). Liquid chromatography-mass spectrometry (LC-MS) was used to unequivocally identify the two Pchlide forms (supplementary Figure S11, C, D). For the species eluting at 22.6 min, we observe dominant m/z peaks of 589, 611 and 643, while for the species eluting at 23.7 min m/z peaks at 591, 613 and 645 were found, yielding a constant mass difference of 2 Da. In comparison MV- and DV-Pchlide have a theoretical mass of 613 Da and 611 Da, respectively. This allows the clear assignment of the 611/613 m/z peaks to DV/MV Pchlide. The m/z peaks at 589/591 and 643/645 are assigned to DV- and MV-Pchlide without its central Mg atom, and the addition of two hydrogens (-24 Da) and to the methanol adducts of DV- and MV-Pchlide (+32 Da), respectively. In all cases, the observed mass difference accounts for the difference in mass between MV- and DV-Pchlide (supplementary Figure S11, C, D). Based on this data we therefore unequivocally assign the two Pchlide species eluting at 22.6 min and 27.3 min as DV-Pchlide and MV-Pchlide, respectively.

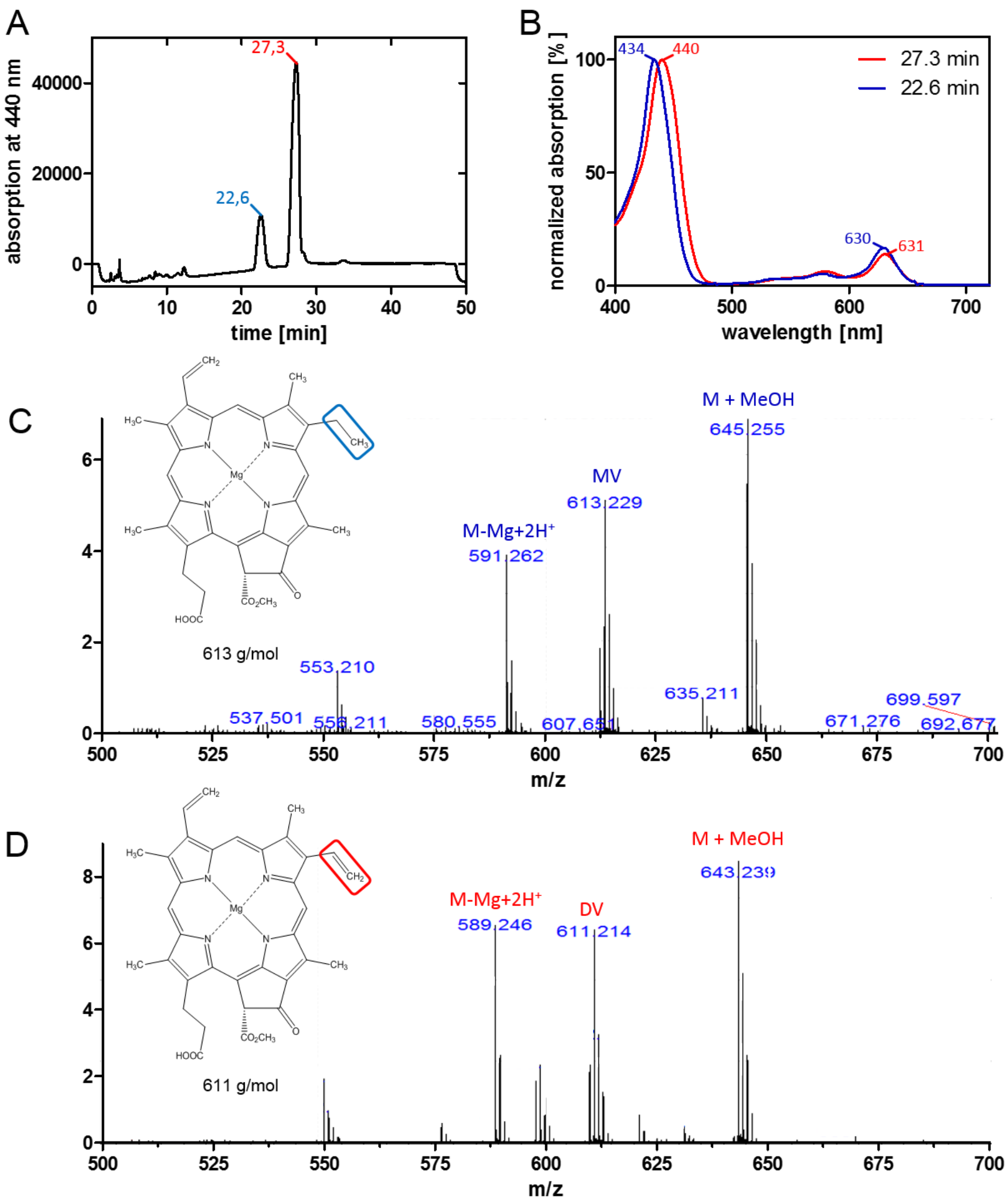

Figure S11: Analytical HPLC and LC-MS analysis of *R. capsulatus* ZY5 produced Pchlide. (A) Reversed-phase HPLC chromatogram illustrating the separation of the MV- and DV-Pchlide on an analytical C30 column. (B) Normalized absorption spectra of the two eluents (22.6 min and 27.3 min) diluted in 20 mM Tris/HCl buffer (pH = 7.5) supplemented with 500 mM NaCl and 20% (v/v) glycerol, and 0.03% (v/v) Triton X-100. MS analysis of the (C) first eluent (22.6 min) and (D) second eluent (27.3 min). The structure of MV- and DV-Pchlide is shown as inset in C and D.

Preparative separation of MV/DV Pchlide

To enable the detailed analysis of the MV/DV-Pchlide acceptance of different LPORs, e.g. by comparative enzyme kinetic studies, both Pchlide forms had to be prepared at larger scale. This was achieved by preparative-scale LC (for details see Materials and Methods). Scale-up of the separation conditions optimized for analytical HPLC to preparative scale, yielded two baseline separated dominant peaks (supplementary Figure S12, A). The resulting DV-Pchlide preparation showed the same red-shift of the Soret and Q_y bands relative to purified MV-Pchlide preparation (supplementary Figure S12, B), verifying successful purification of MV- and DV-

Pchlide. To be able the use of the same MV- and DV-Pchlide preparation in all assays, multiple purification runs were performed and the resulting MV- and DV-Pchlide preparations were pooled before use.

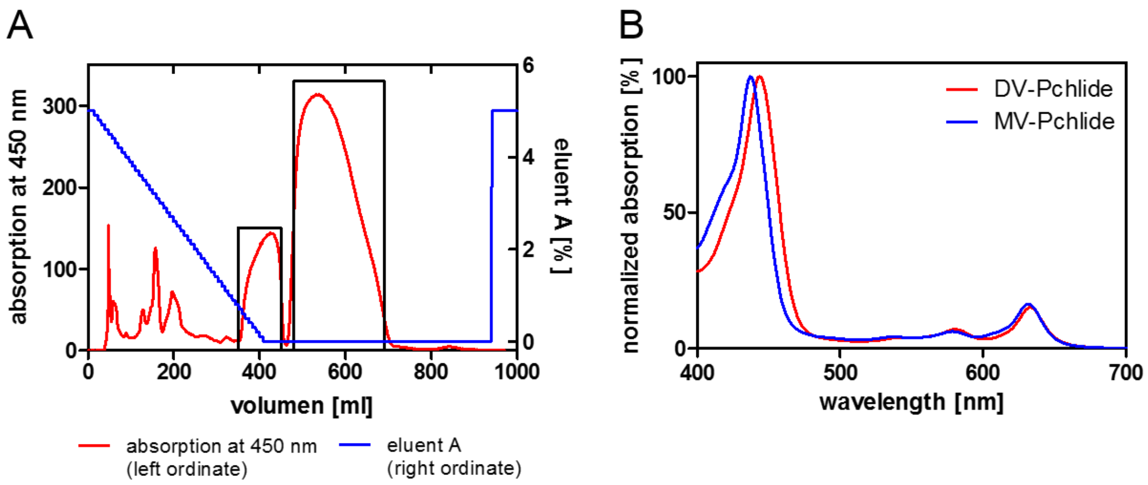

Figure S12: Preparative-scale purification of MV- and DV-Pchlide from the *R. capsulatus* ZY5 produced Pchlide. C30 chromatogram (A) and absorption spectra (B) of purified MV/DV Pchlide diluted in 20 mM Tris/HCl buffer (pH = 7.5) supplemented with 500 mM NaCl and 20% (v/v) glycerol, and 0.03% (v/v) Triton X-100.

MV/DV Pchlide substrate acceptance

The acceptance of MV- and DV-Pchlide by the different LPORs was tested by determining the specific activity of the purified enzymes at a fixed substrate concentration of 3.5(± 0.15) µM MV- or DV-Pchlide, respectively. For each substrate the initial rate velocity was obtained from the linear regime of the conversion curves below 10% conversion. The resulting values are expressed as U/mg (supplementary Figure S13, supplementary Table S9). We consider a variant to show a preference if the fold-difference between the respective activities is > 1.5 (supplementary Table S9).

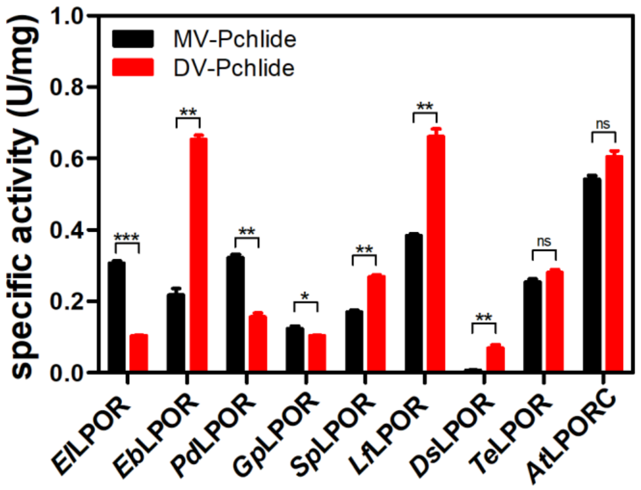

Figure S13: MV/DV Pchlide substrate acceptance determined by measuring the activity of the respective enzyme with either MV (black bars) or DV-Pchlide (red bars). Enzymes are identified by LPOR ID (Table S2). All measurements were carried out in triplicate (n=3) with the error bars corresponding to the standard deviation of the mean. Statistical significance (two-tailed, paired t-test, $P<0.05$)

In terms of specific activity, no clear MV- or DV-Pchlide substrate preference was observed for the plant and cyanobacterial enzyme (*Te*LPOR and *At*LPORC, see supplementary Table S9; Table 1 of the main manuscript), which for *Te*LPOR has also been reported before (Heyes, et al. 2006). In contrast, as also apparent from supplementary Figure S13, all AAPB LPORs, with the exception of *Gp*LPOR clearly show different MV/DV-Pchlide substrate preferences, with *El*LPOR, *Pd*LPOR apparently favoring MV- over DV-Pchlide, while *Eb*LPOR, *Sp*LPOR, *Lf*LPOR and *Ds*LPOR seem to favor DV-Pchlide (supplementary Table S9, Table 1 of the main manuscript, supplementary Figure S13).

Table S9: Specific activities for the conversion of MV- and DV-Pchlide by LPORs.

	***El*LPOR**	***Eb*LPOR**	***Pd*LPOR**	***Gp*LPOR**	***Sp*LPOR**	***Lf*LPOR**	***Ds*LPOR**	***Te*LPOR**	***At*LPORC**
MV (U/mg)	0.31 ± 0.01	0.22 ±0.02	0.32 ± 0.01	0.12 ± 0.01	0.17 ± 0.01	0.38 ± 0.01	0.01 ± 0.001	0.54 ± 0.01	0.25 ± 0.01
DV (U/mg)	0.10 ± 0.01	0.65 ± 0.01	0.16 ± 0.01	0.10 ± 0.01	0.27 ± 0.01	0.66 ± 0.02	0.07 ± 0.009	0.61 ± 0.02	0.28 ± 0.01
fold-difference[a]	3.0	3.0	2.1	1.2	1.6	1.7	9.8	1.1	1.1

[a:] fold-difference between the activity determined for MV- and DV-Pchlide

In conclusion, Pchlide substrate binding, the formation and stability of the NADPH/Pchlide/LPOR ternary complex (see Supplementary Materials section 1.3.3), as well as MV/DV-Pchlide substrate preferences seem to be different between AAPB LPORs and their plant and cyanobacterial counterparts.

1.4 Phylogenetic tree reconstruction, evolutionary hypothesis evaluation

1.4.1 Sequence selection and phylogenetic tree reconstruction

In the 116,919 bacterial genomes that were searched for LPOR homologs, we detected 609 putative LPOR sequences: 573 cyanobacterial and 36 AAPB LPORs.

The HMMER search with an LPOR model also identified an LPOR sequence in the PVC group *Planctomycetaceae* bacterium TMED241 (accession number RPG07162.1) and in the genome of actinobacterium *Propionibacteriaceae* bacterium (accession number MAR53084.1).

LPOR sequence of *Planctomycetaceae* bacterium TMED241 is identical to the LPOR sequence of *Synechococcus* sp. KORDI-49. There are only four synonymous substitutions in the corresponding protein coding sequence. Taking this into account and the fact that the genome of *Planctomycetaceae* bacterium TMED241 is derived from a metagenome source, we attribute the occurrence of a LPOR in this bacterium to contamination.

The best blastp hit for actinobacterium LPOR is the LPOR of *Synechococcus* sp. NP17 with 99.68% of sequence identity and full sequence coverage. Moreover, other photosynthetic proteins, which were identified in the genome of *Propionibacteriaceae* bacterium also have best blastp hits among *Synechococcus* species with high percentage of sequence identity (data not shown). Since the genome was also derived from a metagenome source, it seems again plausible to assume a contamination.

From the set of identified LPORs 236 sequences (see supplementary Table S10) were selected for tree inference. This included most AAPB LPORs and a reduced set of cyanobacterial LPORs as outlined in the following. From the list of AAPB LPORs (Figure 1 in the main manuscript; supplementary Table S1) 3 out of 36 were excluded from phylogenetic analysis. *Erythrobacteraceae* bacterium HL-111 and *Erythrobacter* sp. HL-111 possess identical LPOR sequences. We only considered one in the phylogeny. In addition, two genomes of *Porphyrobacter dokdonensis* have identical LPOR sequences and only one was included in the analysis. Similarly, two genomes of *Erythrobacter litoralis* have identical LPORs and only one was used in phylogenetic inference. We also selected only a subset of cyanobacterial LPORs. We excluded sequences, if there were more than three to four LPORs for the same species or if there were more than four species per genus and only if these sequences clustered in monophyletic clades on preliminary trees. In summary, our selection of LPORs covers a wide range of cyanobacterial genera and retains broad sequence diversity. This resulted in a set of 33 AAPB LPORs (including sequences from α- and β-proteobacteria, and *Gemmatimonadetes* FCB group bacteria) and 203 cyanobacteria (from various genera).

Table S10: Overview over the sequences used for tree inference. The complete list of sequences, including accession numbers and species information (species, order, phylum) is provided as separate Excel spread sheet (Table_S10.xlsx)

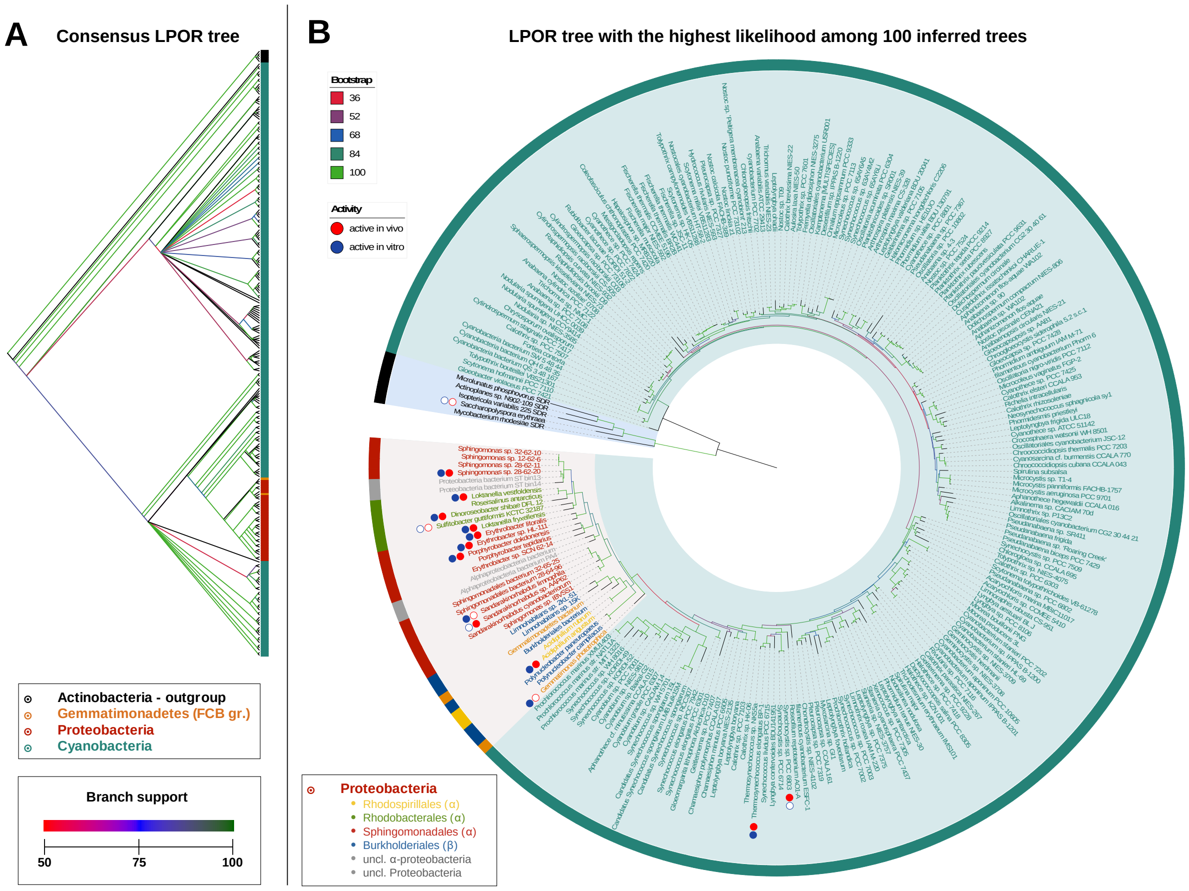

Figure S14: (A) Consensus tree of 100 inferred LPOR trees. The tree was rooted using five SDRs from actinobacteria as outgroups (supplementary Table S10). The branch support is the number of times the branch is observed in 100 trees (denoted by color code). All branches with the support of less than or equal to 50 are collapsed. The multifurcating nodes reflecting unstable parts are only observed within cyanobacteria species (marked by cyan color strip). The AAPB LPORs form a monophyletic clade (marked with red and orange color strip) within a stable cyanobacteria clade (without multifurcating nodes and as denoted by green branches has high support based on 100 trees). (B) Exemplary phylogenetic LPOR tree with mapped activity. The depicted maximum-likelihood tree corresponds to a tree with the highest likelihood among the 100 inferred trees. Branches are colored with regard to branch support (UFBoot support). Leaf labels are colored according to taxonomy as indicated in the figure legend. LPOR activity, as inferred from *in vitro* and *in vivo* assays, was mapped to the tree with filled/empty blue (*in vitro* assay) and red (*in vivo* assay) circles.

1.4.2 Analysis of evolutionary timeline

Table S11: Divergence times for relevant bacterial lineages.

time (MYA)	mean	reference
Node 1 - last common bacterial ancestor (LCBA)		
3460.0	3792,53	(Sheridan, et al. 2003)
3977.0		(Battistuzzi, et al. 2004)
4128.0		(Battistuzzi and Hedges 2009)
4180.2		(Marin, et al. 2017)
3410, 3600		(Betts, et al. 2018)[1]
Node 2 - divergence of cyano-/actinobacteria and proteobacteria/FCB group		
3100.0	3389,16	(Sheridan, et al. 2003)
3152.3		(Battistuzzi and Hedges 2009)
3186.0		(Battistuzzi, et al. 2004)
3254.3		(Marin, et al. 2017)
3591, 3624, 3692, 3692		(Magnabosco, et al. 2018)[2]
3225, 3375		(Betts, et al. 2018)
Node 3 - divergence of FCB group from proteo-/acidobacteria lineage		
3017.8	2943,62	(Marin, et al. 2017)
3030.3		(Battistuzzi and Hedges 2009)
3070.0		(Battistuzzi, et al. 2004)
2650, 2950		(Betts, et al. 2018)
Node 4 - α-/β-/γ-proteobacterial and Rhodospirillales (α-proteobacteria) radiation		
606.7	2052,81	(Chriki-Adeeb and Chriki 2016)
2360.0		(Sheridan, et al. 2003)
2373.6		(Battistuzzi and Hedges 2009)
2508.0		(Battistuzzi, et al. 2004)
2621.4		(Marin, et al. 2017)
1700, 2200		(Betts, et al. 2018)
Node 5 - divergence of genus Acidiphilium from Acidocella		
96.2		(Marin, et al. 2017)
Node 6 - divergence of Rhodobacterales and Sphingomonadales (α-proteobacteria)		
1467.5	1540.45	(Marin, et al. 2017)
1613.4		(Battistuzzi and Hedges 2009)
Node 7 - divergence of Sphingomonadales genera with LPORs: Sphingomonas, Sandarakinorhabdus, Porphyrobacter, Erythrobacter		
253.2		(Marin, et al. 2017)
Node 8 - divergence of Rhodobacterales genera with LPORs: Dinoroseobacter, Roseisalinus, Sulfitobacter, Loktanella		
142.6		(Marin, et al. 2017)
Node 9 - divergence of Polynucleobacter and Limnohabitans genera (β-proteobacteria)		
872.1	904	(Battistuzzi and Hedges 2009)
935.9		(Marin, et al. 2017)
Node 10 - divergence of Limnohabitans		
0.4		(Marin, et al. 2017)
Node 11 - divergence of Polynucleobacter		
105.2		(Marin, et al. 2017)
Node 12 - divergence of Gemmatimonas and Candidatus Latescibacteria		
1940 (2150, 1625)		(Betts, et al. 2018), Fig. 3

[1] Two values are mentioned, one corresponding to the split-time from Fig. 3 from (Betts et al. 2018) and one for the increased sample (supplementary figure S4.3.c)
[2] Four values are mentioned, corresponding to the entries from Table 3 from (Magnabosco et al. 2018)

Node 13 - crown-group cyanobacteria radiation		
1179.5	2093,51	(Sjöstrand, et al. 2014)
2104.3		(Marin, et al. 2017)
2576.9		(Blank 2013)
2666.0		(Cornejo-Castillo, et al. 2016)
2700.0		(Sanchez-Baracaldo, et al. 2014)
2305, 2244, 2677, 2515		(Magnabosco, et al. 2018)
2071, 1909, 2024, 1741		(Shih, et al. 2017)
1290, 1400		(Betts, et al. 2018)
Node 14 - divergence of Synechococcus elongatus from SynPro + Synechococcus spongiarum		
1484 (1886,1042)[3]		(Sanchez-Baracaldo, et al. 2019)
Node 15 - divergence of SynPro + Synechococcus spongiarum		
801 (1173,527)		(Sánchez-Baracaldo, et al. 2019)

1.4.3 Genomic Island (GI) analysis

To detect if there are any genomic signatures of recent HGTs, we performed an exhaustive search for *genomic islands (GIs)*. GIs are clusters of consecutive genes likely obtained via HGT and often provide traits that help adaptation of bacteria and archaea within a niche (Dobrindt, et al. 2004; Aminov 2011).

Two state of the art approaches were applied for GI identification, namely IslandViewer 4 (Bertelli, et al. 2017) and SIGI-CRF (Waack, et al. 2006). According to a recent review (Bertelli, et al. 2018), IslandViewer 4 as an integrated approach, while IslandPath-DIMOB**,** SIGI-HMM and SIGI-CRF as standalone methods show very high precision. IslandViewer 4 is a webserver and comprises four different methods: Islander (Hudson, et al. 2015), IslandPick (Langille, et al. 2008), IslandPath-DIMOB (Bertelli and Brinkman 2018) and SIGI-HMM (Waack, et al. 2006). Using the SIGI-CRF-AUTO software, which is a package within a COLOMBO pipeline (Waack, et al. 2006), an extensive search for GIs using all possible parameter values (Minimum Dissimilarity (MD) values from 3 to 40; and Minimum Region Length (MRL) values from 5K to 40K with a step of 5K) was performed. SIGI-CRF accepts only one sequence/contig file. Therefore, for draft genomes only the contigs and scaffolds containing LPORs were used for the analysis. MD parameters in SIGI-CRF reflect how much an identified GI is different compared to the rest of the genome. As a general rule SIGI-CRF-AUTO (Waack, et al. 2006) MD values of < 3 are obtained for different organisms of the same species; while species within the same genus have mostly MD values of less than 6; MD values for species from the same class, but different order result in 9 out of 10 cases in MD values < 17.

We looked for GIs that cover genomic regions, where an LPOR-encoding gene is located. Typically, the search for GIs is performed on completely assembled genomes. Unfortunately, only five out of 36corresponding AAPB genomes were completely assembled at the time of the current study. Additionally, to these five AAPB genomes, we also included 16 other AAPB genomes with relatively long scaffolds covering the LPOR gene (supplementary Figure S15). For eight out of 21 AAPBs indeed GIs covering an LPOR gene were identified (supplementary

[3] The times in the brackets are the 95% confidence interval reported by the original article for the corresponding time estimate.

Table S12, supplementary Figure S16). That is, the corresponding genomic regions are found to be “alien” compared to the rest of the genome/scaffold.

We did not detect cyanobacteria among the potential donors for the identified GIs (supplementary Table S13). This might be due to genome amelioration, which indirectly supports the idea of an ancient HGT. Genome amelioration is the process, which over time adapts the composition of the acquired DNA to the one of the host genome, and as a result, might lead to false-negatives using composition bias based methods (Langille, et al. 2010) for GI detection. Instead of cyanobacteria, many potential donors were from α-proteobacteria (supplementary Table S13).

In fact, only three bacteria (*Erythrobacter* sp. HL-111, *P. dokdonensis* DSM 17193, *Sphingomonas* sp. IBVSS1) had among their potential donors, bacteria with LPORs. Out of the three only *Erythrobacter* sp. HL-111 clusters with its suggested donor *E. litoralis* (Figure 5B) in the phylogenetic tree. *Sphingomonas* sp. IBVSS1 clusters in the vicinity of the suggested donors (*S. limnophila* and *Sandarakinorhabdus* sp. AAP62) and far from other *Sphingomonas* species (Figure 5A). This indeed supports potential HGT from the suggested donors, however, from the phylogeny it is not clear, which bacterium is actually the donor. *P. dokdonensis* DSM 17193 does not cluster with its suggested donor *E. litoralis*. Moreover, GI analysis of a different strain *P. dokdonensis* DSW-74 with identical LPOR sequence did not confirm *E. litoralis* as donor. Instead, the only suggested donor was *Variovorax sp.* URHB0020 (β-proteobacteria), which does not possess a known LPOR.

Note, that no GI that covers an LPOR gene was identified for *G. phototrophica*. This, however, might be due to genome amelioration (i.e. false-negative) and indirectly support the idea of an ancient HGT to *G. phototrophica.*

Overall, eight out of 21 AAPBs showed “alien” signatures in the regions covering the LPOR gene, suggesting intra-clade HGTs. No cyanobacterium was identified as a potential donor for HGT to AAPBs (possibly due to ancient HGT and genome amelioration). In similar analyses performed for cyanobacteria, only one cyanobacterium out of 23 tested showed a potential GI covering an LPOR gene (supplementary Table S13) and, moreover, the suggested donor (γ-proteobacterium) does not possess a known LPOR (i.e. possibly false-positive donor). The absence of GIs supporting HGT between cyanobacteria corroborates an ancient origin of LPOR in cyanobacteria, e.g. with the enzyme having emerged within the cyanobacterial stem.

Table S12: Summary of the GI analysis. Table entries indicate if the corresponding method identified an LPOR-containing GI. Dashes denote that no results were obtained with the corresponding method. Note, that no results are available from Islander (included in IslandViewer 4). Therefore, it is not mentioned in the table.

Taxonomy	Species	IslandViewer 4			SIGI-CRF
		IslandPick	IslandPath-DIMOB	SIGI-HMM	
Gemmatimonadetes	*Gemmatimonas phototrophica*	-	no	no	no
Alphaproteobacteria	*Erythrobacter* sp. HL-111	no	yes	-	yes
Alphaproteobacteria	*Erythrobacter litoralis*	-	no	no	no
Betaproteobacteria	*Polynucleobacter paneuropaeus*	yes	no	no	yes
Alphaproteobacteria	*Dinoroseobacter shibae*	-	no	no	no
Alphaproteobacteria	*Sulfitobacter guttiformis*	-	no	no	yes
Alphaproteobacteria	*Porphyrobacter dokdonensis* DSM 17193	-	no	no	yes
Betaproteobacteria	*Limnohabitans* sp. 15K	-	no	no	no
Alphaproteobacteria	*Sphingomonadales* bacterium 28-64-96	-	no	no	no
Alphaproteobacteria	*Sphingomonas* sp. IBVSS1	-	no	no	yes
Alphaproteobacteria	*Sphingomonadales* bacterium 32-65-25	-	no	no	no
Betaproteobacteria	*Polynucleobacter campilacus*	-	no	no	no
Alphaproteobacteria	*Porphyrobacter dokdonensis* DSW-74	-	yes	no	yes
uncl. Proteobacteria	Proteobacteria bacterium ST_bin13	-	no	no	yes
Betaproteobacteria	*Limnohabitans* sp. 2KL-51	-	no	no	no
Alphaproteobacteria	*Porphyrobacter tepidarius*	-	no	no	no
Alphaproteobacteria	*Erythrobacter litoralis*	-	no	no	no
Alphaproteobacteria	Alphaproteobacteria bacterium PA4	-	no	no	no
Alphaproteobacteria	*Sphingomonas* sp. 32-62-10	-	no	no	yes
Alphaproteobacteria	*Sandarakinorhabdus limnophila*	-	no	no	no
Alphaproteobacteria	*Sandarakinorhabdus* sp. AAP62	-	no	no	no

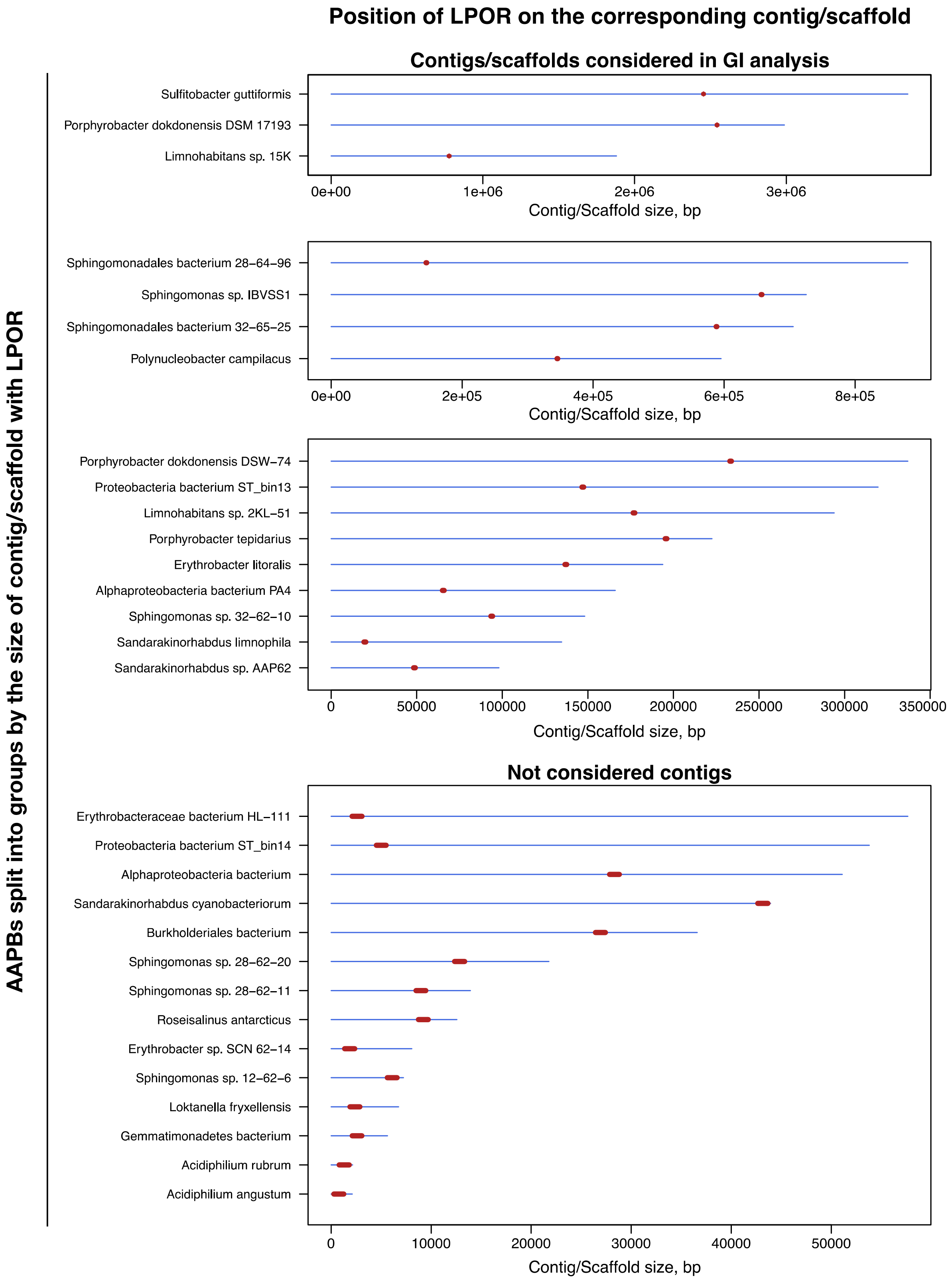

Figure S15: LPOR-containing contigs and scaffolds among selected LPOR containing AAPBs. The position of the LPORs are marked in red on each contig/scaffold. GI - genomic islands.

Table S13: Detailed results of the genomic island (GI) analysis for 21 AAPB and 23 cyanobacterial species. Results available as separate Excel spread sheet (supplementary Table_S13.xlsx)

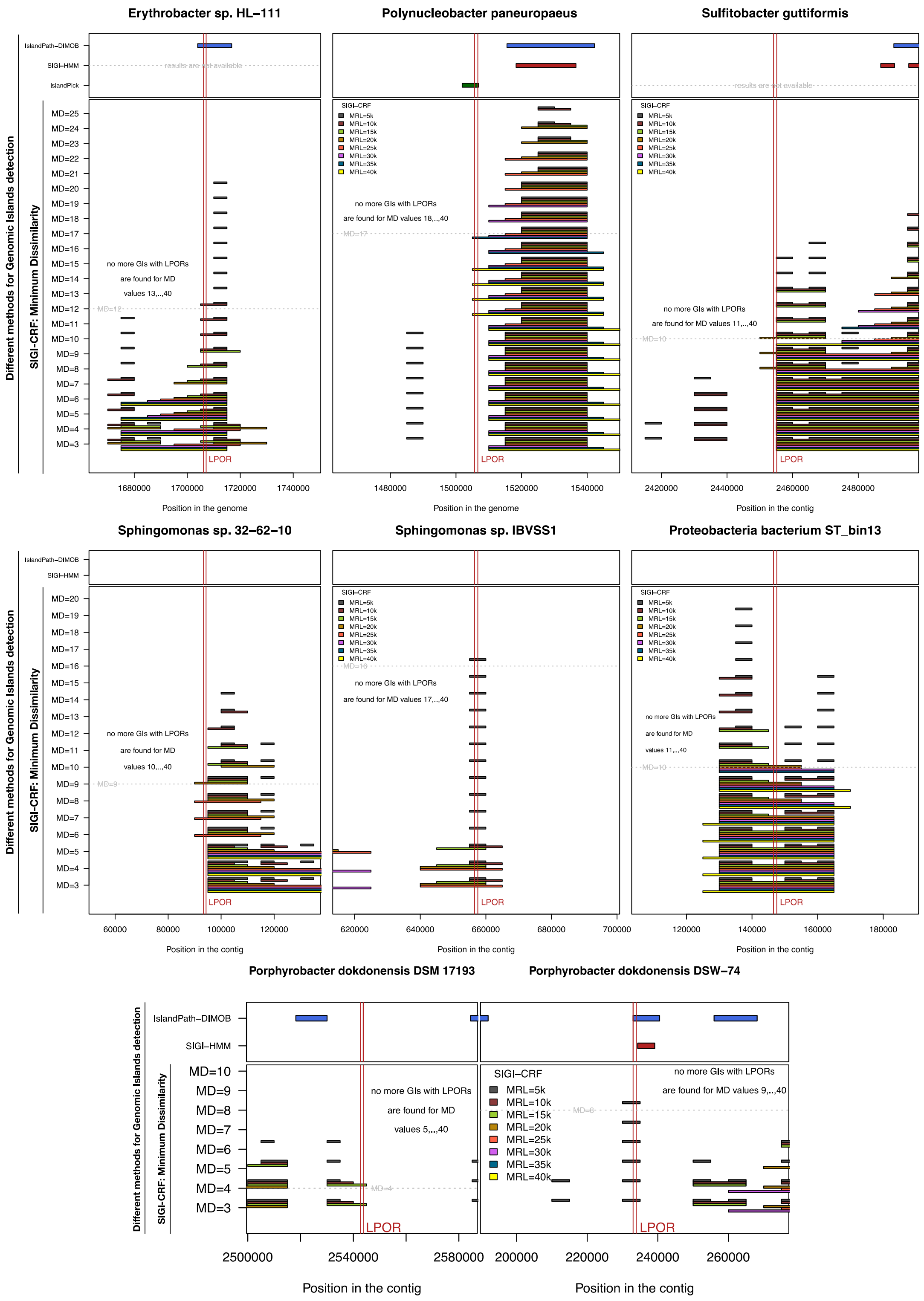

Figure S16: Visualization of genomic islands (GIs) in the vicinity of the LPOR encoding gene for 8 species, where at least one method identified an LPOR-containing GI. The location of the LPOR encoding gene is marked by red vertical lines. The GIs are represented by colored blocks. The upper plots on each subfigure show GIs identified with IslandViewer 4. The lower plots on each subfigure show the GI identified by SIGI-CRF using different parameters. For each species the highest value for the Minimum Dissimilarity (MD) parameter is given for which an LPOR-containing GI could be identified (marked by grey dashed lines). The y-labels are the same for all sub-figures in the corresponding row.

1.4.4. Additional analysis with alternative methods

Additionally to maximum likelihood trees, we also inferred trees using TNT ((Goloboff and Catalano 2016); parsimony), MEGA ((Kumar, et al. 2012); neighbour-joining) and MrBayes ((Ronquist, et al. 2012); Bayesian phylogeny). TNT reported 865 equally parsimonious trees. A subtree of the majority rule consensus tree is provided in Figure S17B. All parsimonious trees support the monophyletic AAPB-clade within picocyanobacteria. The same is true for the NJ tree (Figure S17C). We ran MrBayes with two runs and 4 chains for 6 million generations. As inspected using Tracer (v1.7.1, (Rambaut, et al. 2018)) one of the runs converged. We built a majority rule consensus tree of 9002 trees (after burn-in of 25%) from this run. The tree also supports monophyletic AAPB-LPOR-clade and its position within picocyanobacteria (Figure S17D).

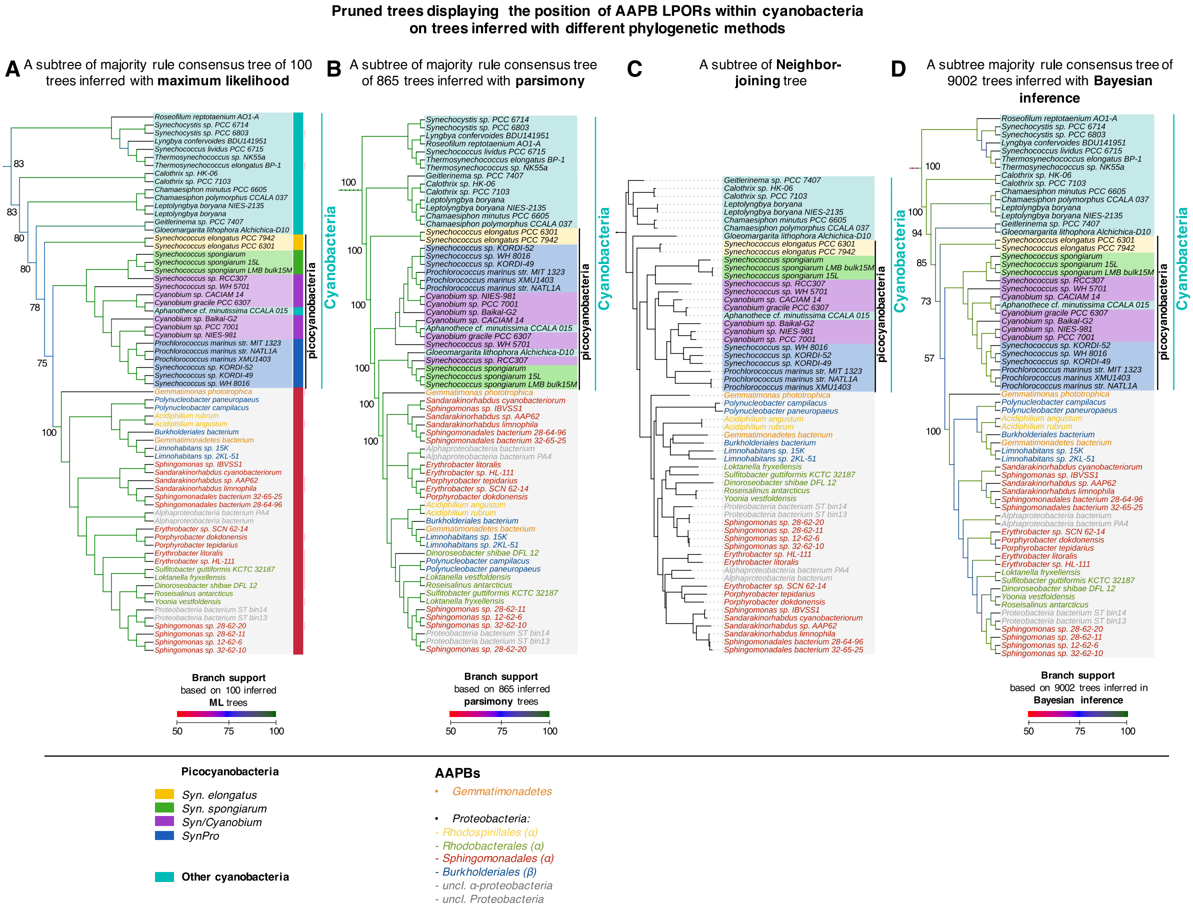

Figure S17: Pruned subtrees display the position of AAPB-clade in LPOR-trees inferred using alternative methods. The branch support is based on the trees used to infer the majority consensus trees for the respective method. The numbers correspond to support values for the relevant branches. For the support of remaining branches refer to the color-code.

1.4.5. Additional tree topology tests

Since there are no specific alternative trees to be tested, we varied the position of the AAPB-clade on the tree with the highest likelihood among 100 inferred ML trees. To this end, we moved the AAPB-clade along the backbone of the tree both towards the root and towards its tips within picocyanobacteria clade. The AAPB-clade was either attached to the backbone itself or as a sister clade to the clades attached to the backbone. This resulted in 52 alternative trees plus the initial ML tree. We then applied the approximately unbiased (AU) tree topology test. According to the AU-test 5 trees were not rejected at the 5% level of significance and the ML tree has with p=0.723 the highest p-value, followed by the tree where the AAPB-clade is sister group of the picocyanobacteria (p-value = 0.651). The remaining three trees have much lower p-values, namely: 0.199, 0.136, 0.0571. Figure S18 displays the alternative placements of the AAPB-clade in the phylogeny together with the p-values. Overall, AAPB-LPORs grouping within picocyanobacteria (ML-tree) or as sister to all picocyanobacteria support our suggestion that AAPBs originally obtained their LPOR from picocyanobacteria, although not all alternative tree topologies could be rejected (see above).

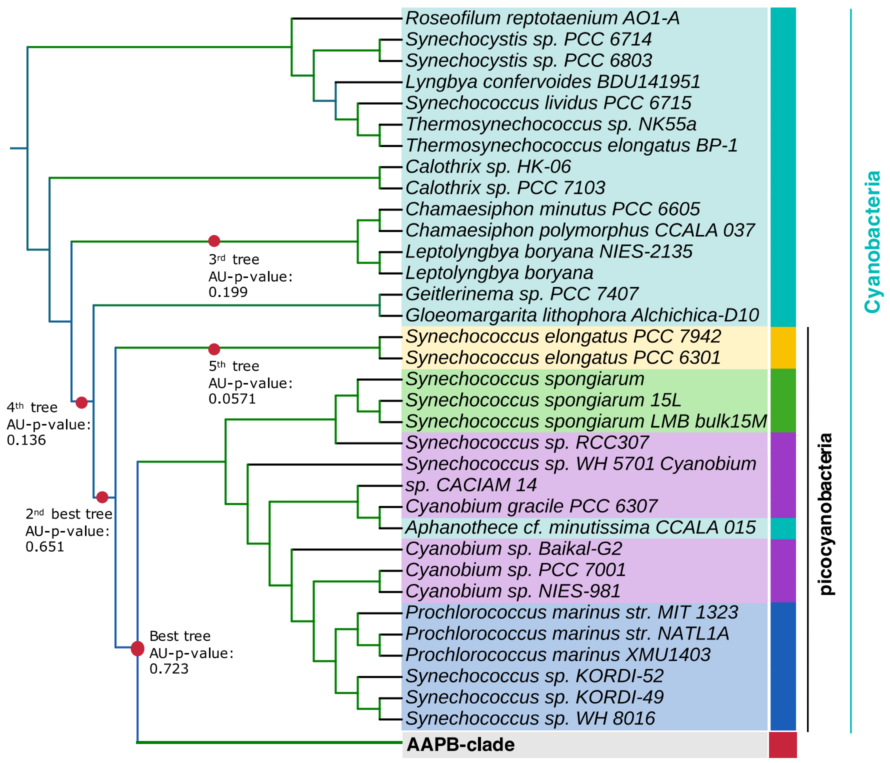

Figure S18: Position of AAPB-clade on alternative trees, which survived the AU-test. The positions are marked by red circles with the corresponding AU-p-value. The best tree (highest AU-p-value) corresponds to the tree with the highest likelihood among 100 inferred ML trees.

1.5 Small angle X-ray scattering (SAXS) analyses

To identify the best fitting model, we compared our experimental SAXS data against theoretical scattering curves computed for a variety of different *Te*LPOR, *Ds*LPOR and *El*LPOR models. Those were generated either based on the previously published model of the LPOR of *Synechocystis* sp. (*Ss*LPOR) (Townley, et al. 2001; Schneidewind, et al. 2019), recently published (*Te*LPOR of *Thermosynechococcus elongatus* BP-1, PDB ID: 6RNV (Zhang, et al. 2019), or deposited (*Ss*LPOR of *Synechocystis* sp. PCC 6803, PDB-ID: 6R48; unpublished) LPOR X-ray structures as templates. Due to the inherent flexibility of LPORs, all present X-ray structures lack certain sequence stretches, which could not be traced in electron density. Therefore, to fill in those gaps and generate full-length models, those missing loops were modelled by YASARA loop modelling. Best-fitting models were identified based on CRYSOL-derived χ values for the fit of the respective theoretical scattering curve against the experimental SAXS data, with lower χ values indicative of a better fitting model (Table S15). Interestingly, all models built based on the most complete LPOR X-ray structure (*Ss*LPOR, PDB: 6R48; unpublished), did show higher χ values (worse fit) as the models built based on either the *Ss*LPOR homology model of Townley and co-workers (Townley, et al. 2001) or the recent TeLPOR X-ray structure (PDB: 6RNV; (Zhang, et al. 2019)). The overall rather large χ values (in most cases $\chi > 2$) suggest that all models to a certain degree fit the experimental data, but none does significantly better account for the data. The only significant improvement in χ is seen, when the C-terminal extension, which is missing in all models built based on the *Ss*LPOR homology model of Townley and co-workers (Townley, et al. 2001), is modeled as protruding α-helix as described recently (Schneidewind, et al. 2019). To account for these uncertainties we decided to only depict the core domain data and models in the main manuscript to stress only the observation that the here newly identified AAPB LPORs possess a solution structure very similar to the cyanobacterial LPOR enzyme. This is also corroborated by the observation of highly similar *ab initio* models (Figure 4, lower panels) derived from SAXS data using the programs DAMMIF, DAMMIN and GASBORP of the ATSAS package (Petoukhov, et al. 2012). Superimposition of the Rossman-fold core domain homology models to the corresponding low-resolution envelopes suggests a placement of the conserved LPOR Rossman-fold within the larger subdomain. The theoretical scattering curves calculated based on the respective homology model agree reasonably well with the experimental data with χ values between 2.535 (*El*LPOR-cHis) and 3.198 (*Ds*LPOR), with smaller χ values corresponding to a better fit. As suggested recently for *Te*LPOR (Schneidewind, et al. 2019), a flexible C-terminal helical extension or other flexible loop regions within the protein might fit into the small subdomain. Since for *El*LPOR and *El*LPOR-cHis essentially identical scattering curves and *ab initio* models were obtained (compare Figure 4, panel B and C), the smaller subdomain seen in all envelopes is likely constituted by C-terminal structural part not covered by homology modelling. This is also corroborated by the smaller theoretical radius of gyration (*R*g) calculated for the respective homology models compared to the SAXS determined value (Table 2).

Table S14: Protein concentra tion of LPOR samples used for SAXS measurements.

LPOR	concentration [mg/ml]			
*Te*LPOR	0.40	0.91*	3.17	6.83*
*Ds*LPOR	0.56	1.21	2.94*	5.21*
*El*LPOR	0.52	0.90*	3.11	5.96*
*El*LPOR-cHis	0.45	0.97*	3.53	7.48*

*datasets combined for analysis

Table S15: CRYSOL-derived χ values for the fit of the theoretical scattering curve, computed for a variety of LPOR models, against the experimental SAXS data for the corresponding protein.

Protein	χ [core domain][a]	χ [full-length][b]	χ [*Te*LPOR, PDB: 6RNV][c]	χ [*Ss*LPOR, PDB: 6R48][d]
	template: homology model		template: X-ray structures	
*Te*LPOR	2.864	1.654[e]	2.546	3.483
*Ds*LPOR	3.198	1.403	2.904	3.743
*El*LPOR	2.649	1.564	2.050	3.092
*El*LPOR-cHis	2.535	2.434	1.535	2.646

[a:] core domain models (residues 1-280 of *Te*LPOR) built based on the *Ss*LPOR homology model by (Townley, et al. 2001); [b:] C-terminally extended, full-length homology models built in accordance with (Schneidewind, et al. 2019); [c:] full-length models built based on the recently published *Te*LPOR X-ray structure (PDB-ID: 6RNV; (Zhang, et al. 2019)); [d:] full-length models built based on the recently deposited *Ss*LPOR X-ray structure (PDB-ID: 6R48; unpublished); e: according to (Schneidewind, et al. 2019)

Table S16: pH and temperature growth (optima) characteristics of selected LPOR-containing AAPBs. LPOR biochemical properties are shown in blue, growth characteristics in red. The reference from which the growth information was obtained is shown in bold.

Species	AAPB classification main source	Additional articles and strain info	pH range LPOR / growth	temp range LPOR / growth
Gemmatimonas phototrophica *Gp*LPOR	**(Zeng, et al. 2014**), semiaerobic conditions	habitat: isolated from near-shore lake water at a depth of 0.5 m from the freshwater Swan Lake, which is located at the northern margin of the western Gobi Desert (Zeng, et al. 2014)	8.0 ± 2.5 / 6.0-9.0 (opt. 7.5-8)	16.2 ± 7.4°C / 16-30°C(opt.25-30°C)
Erythrobacter litoralis *El*POR	**(Wang, et al. 2014)**	Shown for strain T4: (**Yurkov, et al. 1994**) The type strain is T4 (= DSM 8509) habitat: marine cyanobacterial mat in a supralitoral zone. This organism is able to grow in a very wide salinity range, from 5‰ (freshwater) to 96‰	8.5 ± 2.5 / -	23.3 ± 7.1°C / (25-30°C)
Porphyrobacter dokdonensis DSM 17193 *Pd*LPOR	(**Yoon, et al. 2006**)	habitat: isolated from Seawater off Dokdo in the East Sea (Sea of Korea); shows optimal growth at slightly halophilic conditions (Song, et al. 2016).	8.0 ± 2.5 / 5.5-8.0 (opt. 7.0-8.0)	19.6 ± 7.1°C / 10-43°C(opt.35-37°C)
Dinoroseobacter shibae DFL12^{T} *Ds*LPOR	**(Biebl, et al. 2005)**	A member of the globally important marine *Roseobacter* clade, symbiont of cosmopolitan marine microalgae, including toxic dinoflagellates (Wagner-Döbler, et al. 2010)	7.0 ± 2.0 / 6.5-8.8	16.2 ± 7.4°C / 15-38°C (opt. 33°C)
Loktanella fryxellensis *Lf*LPOR	**(Van Trappen, et al. 2004)** different strains were shown to be aerobic:	habitat: isolated from microbial mats in the Antarctic Lakes Fryxell,; tolerance towards high salinity (Van Trappen, et al. 2004)	9.0 ± 3.0 / -	23.3 ± 7.7°C / 5-25°C (opt. 25°C, but weak at 30°C)
Sphingomonas sp. 28-62-20	putative AAPB	related to *Sphingomonas* sp. S17: whose habitat is: alkaline, hyperarsenic, and hypersaline volcano-associated lake at high altitude in the Argentinean Puna (Farias, et al. 2011) "drastic temperature changes"	8.0 ± 2.5 / -	23.3 ± 3.7°C / -

2. Supplementary References

Aminov RI. 2011. Horizontal gene exchange in environmental microbiota. Front Microbiol 2:158.

Battistuzzi FU, Feijao A, Hedges SB. 2004. A genomic timescale of prokaryote evolution: insights into the origin of methanogenesis, phototrophy, and the colonization of land. BMC Evol Biol 4:44.

Battistuzzi FU, Hedges SB. 2009. A major clade of prokaryotes with ancient adaptations to life on land. Molecular Biology and Evolution 26:335-343.

Bertelli C, Brinkman FSL. 2018. Improved genomic island predictions with IslandPath-DIMOB. Bioinformatics 34:2161-2167.

Bertelli C, Laird MR, Williams KP, Simon Fraser University Research Computing G, Lau BY, Hoad G, Winsor GL, Brinkman FSL. 2017. IslandViewer 4: expanded prediction of genomic islands for larger-scale datasets. Nucleic Acids Res 45:W30-W35.

Bertelli C, Tilley KE, Brinkman FSL. 2018. Microbial genomic island discovery, visualization and analysis. Brief Bioinform.

Betts HC, Puttick MN, Clark JW, Williams TA, Donoghue PCJ, Pisani D. 2018. Integrated genomic and fossil evidence illuminates life's early evolution and eukaryote origin. Nature Ecology & Evolution 2:1556-1562.

Biebl H, Allgaier M, Tindall BJ, Koblizek M, Lunsdorf H, Pukall R, Wagner-Dobler I. 2005. *Dinoroseobacter shibae* gen. nov., sp. nov., a new aerobic phototrophic bacterium isolated from dinoflagellates. Int J Syst Evol Microbiol 55:1089-1096.

Blank CE. 2013. Origin and early evolution of photosynthetic eukaryotes in freshwater environments: reinterpreting proterozoic paleobiology and biogeochemical processes in light of trait evolution. J Phycol 49:1040-1055.

Buhr F, El Bakkouri M, Valdez O, Pollmann S, Lebedev N, Reinbothe S, Reinbothe C. 2008. Photoprotective role of NADPH:protochlorophyllide oxidoreductase A. Proc Natl Acad Sci U S A 105:12629-12634.

Chriki-Adeeb R, Chriki A. 2016. Estimating divergence times and substitution rates in rhizobia. Evol Bioinform Online 12:87-97.

Cornejo-Castillo FM, Cabello AM, Salazar G, Sanchez-Baracaldo P, Lima-Mendez G, Hingamp P, Alberti A, Sunagawa S, Bork P, de Vargas C, et al. 2016. Cyanobacterial symbionts diverged in the late Cretaceous towards lineage-specific nitrogen fixation factories in single-celled phytoplankton. Nat Commun 7:11071.

Dobrindt U, Hochhut B, Hentschel U, Hacker J. 2004. Genomic islands in pathogenic and environmental microorganisms. Nat Rev Microbiol 2:414-424.

Farias ME, Revale S, Mancini E, Ordonez O, Turjanski A, Cortez N, Vazquez MP. 2011. Genome sequence of *Sphingomonas* sp. S17, isolated from an alkaline, hyperarsenic, and hypersaline volcano-associated lake at high altitude in the Argentinean Puna. J Bacteriol 193:3686-3687.

Ford C, Mitchell S, Wang WY. 1983. Characterization of NADPH - Protochlorophyllide Oxidoreductase in the Y-7 and Pc-1y-7 Mutants of *Chlamydomonas-reinhardtii*. Molecular & General Genetics 192:290-292.

Goloboff PA, Catalano SA. 2016. TNT version 1.5, including a full implementation of phylogenetic morphometrics. Cladistics 32:221-238.

Heyes DJ, Kruk J, Hunter CN. 2006. Spectroscopic and kinetic characterization of the light-dependent enzyme protochlorophyllide oxidoreductase (POR) using monovinyl and divinyl substrates. Biochem J 394:243-248.

Heyes DJ, Martin GE, Reid RJ, Hunter CN, Wilks HM. 2000. NADPH:protochlorophyllide oxidoreductase from *Synechocystis*: overexpression, purification and preliminary characterisation. FEBS Lett 483:47-51.

Heyes DJ, Menon BR, Sakuma M, Scrutton NS. 2008. Conformational events during ternary enzyme-substrate complex formation are rate limiting in the catalytic cycle of the light-driven enzyme protochlorophyllide oxidoreductase. Biochemistry 47:10991-10998.

Hudson CM, Lau BY, Williams KP. 2015. Islander: a database of precisely mapped genomic islands in tRNA and tmRNA genes. Nucleic Acids Res 43:D48-53.
Kaschner M, Loeschcke A, Krause J, Minh BQ, Heck A, Endres S, Svensson V, Wirtz A, von Haeseler A, Jaeger KE, et al. 2014. Discovery of the first light-dependent protochlorophyllide oxidoreductase in anoxygenic phototrophic bacteria. Mol Microbiol 93:1066-1078.
Katzke N, Arvani S, Bergmann R, Circolone F, Markert A, Svensson V, Jaeger KE, Heck A, Drepper T. 2010. A novel T7 RNA polymerase dependent expression system for high-level protein production in the phototrophic bacterium *Rhodobacter capsulatus*. Protein Expr Purif 69:137-146.
Klipp W, Masepohl B, Pühler A. 1988. Identification and mapping of nitrogen fixation genes of *Rhodobacter capsulatus*: duplication of a nifA-nifB region. J Bacteriol 170:693-699.
Kumar S, Stecher G, Peterson D, Tamura K. 2012. MEGA-CC: computing core of molecular evolutionary genetics analysis program for automated and iterative data analysis. Bioinformatics 28:2685-2686.
Langille MG, Hsiao WW, Brinkman FS. 2008. Evaluation of genomic island predictors using a comparative genomics approach. Bmc Bioinformatics 9:329.
Langille MGI, Hsiao WWL, Brinkman FSL. 2010. Detecting genomic islands using bioinformatics approaches. Nat Rev Microbiol 8:372-382.
Magnabosco C, Moore KR, Wolfe JM, Fournier GP. 2018. Dating phototrophic microbial lineages with reticulate gene histories. Geobiology 16:179-189.
Marin J, Battistuzzi FU, Brown AC, Hedges SB. 2017. The Timetree of Prokaryotes: New Insights into Their Evolution and Speciation. Molecular Biology and Evolution 34:437-446.
McFarlane MJ, Hunter CN, Heyes DJ. 2005. Kinetic characterisation of the light-driven protochlorophyllide oxidoreductase (POR) from *Thermosynechococcus elongatus*. Photochem Photobiol Sci 4:1055-1059.
Menon BR, Davison PA, Hunter CN, Scrutton NS, Heyes DJ. 2010. Mutagenesis alters the catalytic mechanism of the light-driven enzyme protochlorophyllide oxidoreductase. Journal of Biological Chemistry 285:2113-2119.
Menon BR, Hardman SJ, Scrutton NS, Heyes DJ. 2016. Multiple active site residues are important for photochemical efficiency in the light-activated enzyme protochlorophyllide oxidoreductase (POR). J Photochem Photobiol B 161:236-243.
Menon BR, Waltho JP, Scrutton NS, Heyes DJ. 2009. Cryogenic and laser photoexcitation studies identify multiple roles for active site residues in the light-driven enzyme protochlorophyllide oxidoreductase. Journal of Biological Chemistry 284:18160-18166.
Petoukhov MV, Franke D, Shkumatov AV, Tria G, Kikhney AG, Gajda M, Gorba C, Mertens HDT, Konarev PV, Svergun DI. 2012. New developments in the ATSAS program package for small-angle scattering data analysis. Journal of Applied Crystallography 45:342-350.
Rambaut A, Drummond AJ, Xie D, Baele G, Suchard MA. 2018. Posterior Summarization in Bayesian Phylogenetics Using Tracer 1.7. Syst Biol 67:901-904.
Ronquist F, Teslenko M, van der Mark P, Ayres DL, Darling A, Hohna S, Larget B, Liu L, Suchard MA, Huelsenbeck JP. 2012. MrBayes 3.2: efficient Bayesian phylogenetic inference and model choice across a large model space. Syst Biol 61:539-542.
Sanchez-Baracaldo P, Bianchini G, Di Cesare A, Callieri C, Chrismas NAM. 2019. Insights Into the Evolution of Picocyanobacteria and Phycoerythrin Genes (mpeBA and cpeBA). Front Microbiol 10:45.
Sanchez-Baracaldo P, Ridgwell A, Raven JA. 2014. A neoproterozoic transition in the marine nitrogen cycle. Curr Biol 24:652-657.
Schneidewind J, Krause F, Bocola M, Stadler AM, Davari MD, Schwaneberg U, Jaeger KE, Krauss U. 2019. Consensus model of a cyanobacterial light-dependent protochlorophyllide oxidoreductase in its pigment-free apo-form and photoactive ternary complex. Commun Biol 2:351.
Sheridan PP, Freeman KH, Brenchley JE. 2003. Estimated minimal divergence times of the major bacterial and archaeal phyla. Geomicrobiology Journal 20:1-14.
Shih PM, Hemp J, Ward LM, Matzke NJ, Fischer WW. 2017. Crown group Oxyphotobacteria postdate the rise of oxygen. Geobiology 15:19-29.

Simon R, Priefer U, Pühler A. 1983. A Broad Host Range Mobilization System for Invivo Genetic-Engineering - Transposon Mutagenesis in Gram-Negative Bacteria. Bio-Technology 1:784-791.
Sjöstrand J, Tofigh A, Daubin V, Arvestad L, Sennblad B, Lagergren J. 2014. A Bayesian Method for Analyzing Lateral Gene Transfer. Syst Biol 63:409-420.
Song JY, Hong J, Kwak MJ, Kwon SK, Kim JF. 2016. Genome sequence of *Porphyrobacter dokdonensis* DSW-74T, isolated from seawater off Dokdo in the East Sea (Sea of Korea). Genome Announc 4.
Tabita FR, Satagopan S, Hanson TE, Kreel NE, Scott SS. 2008. Distinct form I, II, III, and IV Rubisco proteins from the three kingdoms of life provide clues about Rubisco evolution and structure/function relationships. J Exp Bot 59:1515-1524.
Townley HE, Sessions RB, Clarke AR, Dafforn TR, Griffiths WT. 2001. Protochlorophyllide oxidoreductase: a homology model examined by site-directed mutagenesis. Proteins 44:329-335.
Van Trappen S, Mergaert J, Swings J. 2004. *Loktanella salsilacus* gen. nov., sp. nov., *Loktanella fryxellensis* sp. nov. and *Loktanella vestfoldensis* sp. nov., new members of the *Rhodobacter* group, isolated from microbial mats in Antarctic lakes. Int J Syst Evol Microbiol 54:1263-1269.
Waack S, Keller O, Asper R, Brodag T, Damm C, Fricke WF, Surovcik K, Meinicke P, Merkl R. 2006. Score-based prediction of genomic islands in prokaryotic genomes using hidden Markov models. Bmc Bioinformatics 7.
Wagner-Döbler I, Ballhausen B, Berger M, Brinkhoff T, Buchholz I, Bunk B, Cypionka H, Daniel R, Drepper T, Gerdts G, et al. 2010. The complete genome sequence of the algal symbiont *Dinoroseobacter shibae*: a hitchhiker's guide to life in the sea. ISME J 4:61-77.
Wang Y, Zhang R, Zheng Q, Jiao N. 2014. Draft genome sequences of two marine phototrophic bacteria, *Erythrobacter longus* strain DSM 6997 and *Erythrobacter litoralis* strain DSM 8509. Genome Announc 2.
Yang ZM, Bauer CE. 1990. Rhodobacter capsulatus genes involved in early steps of the bacteriochlorophyll biosynthetic pathway. J Bacteriol 172:5001-5010.
Yoon JH, Kang SJ, Lee MH, Oh HW, Oh TK. 2006. *Porphyrobacter dokdonensis* sp. nov., isolated from sea water. Int J Syst Evol Microbiol 56:1079-1083.
Yurkov V, Stackebrandt E, Holmes A, Fuerst JA, Hugenholtz P, Golecki J, Gad'on N, Gorlenko VM, Kompantseva EI, Drews G. 1994. Phylogenetic positions of novel aerobic, bacteriochlorophyll a-containing bacteria and description of *Roseococcus thiosulfatophilus* gen. nov., sp. nov., *Erythromicrobium ramosum* gen. nov., sp. nov., and *Erythrobacter litoralis* sp. nov. Int J Syst Bacteriol 44:427-434.
Zeng YH, Feng FY, Medova H, Dean J, Koblizek M. 2014. Functional type 2 photosynthetic reaction centers found in the rare bacterial phylum *Gemmatimonadetes*. Proc Natl Acad Sci U S A 111:7795-7800.
Zhang C, Kim SK. 2010. Research and application of marine microbial enzymes: status and prospects. Mar Drugs 8:1920-1934.
Zhang S, Heyes DJ, Feng L, Sun W, Johannissen LO, Liu H, Levy CW, Li X, Yang J, Yu X, et al. 2019. Structural basis for enzymatic photocatalysis in chlorophyll biosynthesis. Nature 574:722-725.
